# Interference of metal ions on the bioluminescent signal of firefly, Renilla, and NanoLuc luciferases in high-throughput screening assays

**DOI:** 10.3389/fchem.2024.1436389

**Published:** 2024-11-26

**Authors:** Francesca Canyelles i Font, Krzysztof Żukowski, Masroor A. Khan, Dorota Kwiatek, Jacek L. Kolanowski

**Affiliations:** Institute of Bioorganic Chemistry, Polish Academy of Sciences, Poznań, Poland

**Keywords:** HTS, luciferase, assay interference, bioluminescence, metal ions, screening, NanoLuc, biochemical assay

## Abstract

Bioluminescent high-throughput screening (HTS) assays, based largely on the activity of firefly (FLuc), Renilla (RLuc), and/or NanoLuc (NLuc) luciferases, are widely utilised in research and drug discovery. In this study, we quantify the luciferase-based real-life HTS assay interference from biologically and environmentally relevant metal ions ubiquitously present in buffers, environmental and biological matrices, and as contaminants in plastics and compound libraries. We also provide insights into the cross-effects of metal ions and other key experimental and biological reagents (e.g., buffer types, EDTA, and glutathione) to inform HTS assay design, validation, and data interpretation. A total of 21 ions were screened in three robust HTS assays (“SC” assays) based on the luminescence of FLuc, RLuc, and NLuc luciferases. Three newly optimised HEPES buffer variants (“H” assays) were developed for direct luciferase comparison. Interference in bioluminescent signal generation was quantified by calculating the IC_50_ values from concentration-dependent experiments for selected highly active and relevant metal ions. Metal ion inhibition mechanisms were probed by variations in specific reagents, EDTA, GSH, and the sequence of addition and buffer composition. In this study, we revealed a significant impact of metal ions’ salts on luciferase-mediated bioluminescence, even at biologically and environmentally relevant concentrations. The extent of signal interference largely aligned with the Irving–Williams series of metal ion–ligand affinities (Cu > Zn > Fe > Mn > Ca > Mg), supporting previous reports on metal ion-dependent FLuc inhibition. However, the absolute magnitude and relative extent of signal reduction by metal ions’ salts differed between SC and H assays and between luciferases, suggesting a complex network of metal ions’ interactions with enzymes, substrates, reactants, and buffer elements. The diversity of the tested conditions and variability of responses provided insights into potential interference mechanisms and synergies that may exacerbate or alleviate interference. The beneficial influence of EDTA and the impact of glutathione, present natively in cells, on bioluminescence readout were pinpointed. Given the ubiquity of metal ions in analysed samples, the causative role in false-positive generation in drug discovery, and the wide breadth of luciferase-based assays used in screening, awareness and quantification of metal influence are crucial for developing assay validation protocols and ensuring reliable screening data, ultimately increasing the critical robustness of bioluminescence-based HTS assays.

## 1 Introduction

High-throughput screening (HTS) is a critical element of early drug discovery. It allows for the rapid assessment of compound libraries against specific biological targets or pathological conditions, thus increasing the likelihood of identifying novel drug candidates or molecular interactions. The main objective of HTS campaigns is to identify hits with promising interactions or activities, which warrant further investigation as potential drugs. HTS is utilised in both pharmaceutical and academic settings to expedite drug discovery and target identification and to develop tools for the investigation of mechanisms of disease (Thorne et al., 2010; Roy, 2018). The global HTS market is substantial, with an estimated value of $15.3 billion in 2020 and a projected reach of $25.6 billion by 2025 (Rees, 2021). There are a variety of HTS assays depending on the desired detectable signal, including but not limited to radioactivity, absorption, and/or emission of near-visible light. Among these options, assays relying on UV-vis photon emission (broadly described as “luminescence”) stand out as the most widely used due to their sensitivity and possibility of using responsive probes that produce distinct signals when interacting with a target as proxies for target presence and/or activity. Luminescence’s extensive adaptability to biological targets and ease of automation make it a valuable tool for HTS campaigns that require speed, accuracy, sensitivity, and reliability (Fan and Wood, 2007; Thorne et al., 2010).

**Scheme 1:**
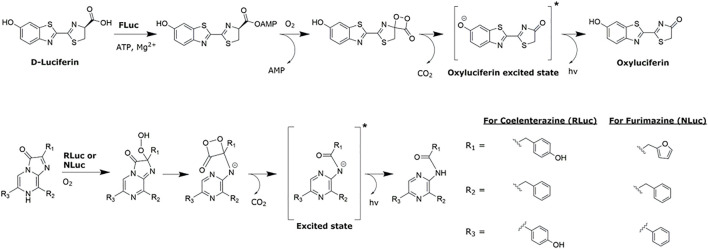
Mechanism of bioluminescence for D-luciferin (FLuc substrate), coelenterazine (RLuc substrate), and furimazine (NLuc substrate). Adopted from the study by Kaskova et al. (2016).

Bioluminescence-based assays are one of the key examples of luminescent assays broadly used in HTS due to their high sensitivity and adaptability to detect a wide range of processes and targets. The most notable examples range from common cell viability assays based on ATP detection with firefly luciferase to assays for gene expression, protein interactions, and target engagement (e.g., nanoBRET methods using NanoLuc) and to a wide range of bio-analyte-specific assays *via* masked-substrate strategies (Yang et al., 2020; Li et al., 2021; Gleneadie et al., 2023; Dunuweera et al., 2024). Bioluminescence is a phenomenon in which photons are emitted due to a biochemical reaction of a luciferase enzyme with its small molecule substrate, luciferin. This catalytic oxygenation leads to the formation of an excited-state intermediate that emits light when returning to the ground state (Scheme 1). The three most commonly used luciferases in HTS are derived from natural sources, namely, firefly luciferase (FLuc), Renilla luciferase (RLuc), and its genetically optimised version NanoLuc (NLuc, Promega Corp. Inc.) (Fan and Wood, 2007; Boute et al., 2016; Kaskova et al., 2016; Li et al., 2021). As bioluminescence relies on enzymes that do not naturally occur in mammalian cells, it ensures bio-orthogonality of the reaction, leading to a very low background and high sensitivity, which, together with high-quantum yields and relative non-toxicity of luciferins, make this system highly attractive for both *in vitro* and *in vivo* applications. The progress in the methods of introduction of genetic modifications and genetic tags to complex biological samples (e.g., through CRISPR/Cas) has contributed to further increases in the number of new bioluminescence-based assays in disease modelling and drug discovery (Ondra et al., 2024).

When running bioluminescence-based HTS assays, it is of utmost importance to reduce interference from non-targets. Such interferents (i.e., “interfering substances/parameters”) can significantly alter the bioluminescence signal emission through direct inhibition of luciferase activity or through interaction with the substrate/product and/or other reagents and reactants, including oxygen, leading to potentially critical artefacts. Among those, certain metal ions and anions have been known to impact the bioluminescent signal. Early on, kinetic studies revealed that the Mg–ATP complex was the preferred substrate in the firefly luciferase reaction, and that the ATP salts of larger group-2 metal ions, such as Ca-ATP or Sr-ATP, showed a reduced bioluminescent output. This was mainly attributed to the interaction of Ca^2+^ or Sr^2+^ with the adenine ring of ATP (Lee et al., 1970). Another observation was that metal ions such as Zn^2+^, Cd^2+^, Hg^2+^, Fe^2+^, Cu^2+^, Co^2+^, and Ni^2+^ have been found to result in a reduction in bioluminescent activity. The authors suggest that the reduction in activity resulting from FeSO_4_ could be attributed to the precipitation of D-luciferin, which is possibly due to the interaction of Fe^2+^ with D-luciferin, rather than the direct influence of Fe^2+^ on luciferase. The reduced activity caused by Cu^2+^, and to some extent by Co^2+^ and Ni^2+^, is attributed to either the direct impact of metal ions on luciferase or competition for ATP, which leads to a decrease in the Mg–ATP concentration (Lee et al., 1970). The inhibitory effect of the group 12 metals on firefly luciferase, on the other hand, was presumed to be due to their reaction with the SH groups of the enzyme (Lee et al., 1970). Gilles et al. (1976) showed that certain anions present in salts and buffers were found to significantly inhibit peak light emission in firefly extracts. They observed that perchlorate, acetate, iodide, and chloride anions induced structural changes in the enzyme. Another study performed by Wen et al. (2001) showed that co-extracted ions with ATP from soils can interfere with firefly bioluminescence. They observed that quenching occurred with several metal ions, such as Cu^2+^ and Zn^2+^, at a concentration of 0.1 mM, and that other heavy metal ions such as Cr^3+^, Ti^3+^, and Hg^2+^ were also able to quench the firefly luciferase-mediated bioluminescent emission. Another quantitative study of metal ions quenching of firefly luciferase bioluminescence revealed that divalent metal ions such as Hg^2+^, Zn^2+^, Cd^2+^, Ni^2+^, Co^2+^, and Fe^2+^ can impact the bioluminescent reaction (Wang et al., 2011). It is also worth mentioning that the bioluminescent reaction may be affected by the given ionic strength and the pH of the tested metal salt solution (Viviani et al., 2018; Altamash et al., 2021). Despite some work on the ionic interference with FLuc-mediated bioluminescence, for RLuc and NLuc, the studies regarding the impact of metal ions on their bioluminescence are limited. In addition, no comprehensive bioluminescence interference studies in HTS-format assays in the context of screening interference were performed for any of the three enzymatic detection systems.

The impact of metal ions on bioluminescence-based HTS assays remains, therefore, a critical gap in research. Failure to address this gap could lead to wasted time, effort, and resources by pursuing compounds that may appear to be hits (false positives) over biologically relevant hits or by discarding compounds (false negatives) due to the absence of activity, all due to potential interference from metal ions. It is essential to understand the potential sources of false-positives and false-negatives in screening assays and to implement rigorous assay validation protocols to ensure the reliability of the screening data. Potential sources of metal ions, such as those found in assay solutions, materials, or reagents, could interfere with bioluminescence-based HTS assays, leading to false-positive/false-negative readouts. Metal ion impurities have been found in many compounds that constitute chemical libraries and/or have come out as hits in screening campaigns, leading to significant loss of resources failure of drug development campaigns (Hermann et al., 2013; Molyneux et al., 2022). Some metal ions (especially heavy metals such as cadmium or lead) could also be present in plastics as additives (Salmela and Vuori, 1979; Olivieri et al., 2012). Therefore, it is crucial to determine the influence of metal ions on bioluminescence-based assays using FLuc, RLuc, or NLuc.

In this work, various metal ion salts were tested in specific concentration ranges and underwent experimental bioluminescence-based HTS assay conditions established previously (https://www.eu-openscreen.eu/services/bioprofiling-assays.html); three new HEPES buffer variants of those assays were also included to enable more direct comparison between luciferases. The main objective of this research was to pinpoint possible interfering metal ion salts that could impair bioluminescence-based HTS assays involving FLuc, RLuc, or NLuc as sources of potential artefacts. Identifying these interfering metal ions will enable research workers to prevent compromised interpretations and optimise the design of bioluminescence-based HTS assays, ultimately increasing their robustness and saving time and resources by minimising false-positives in early stages of drug discovery.

## 2 Materials and methods

### 2.1 Materials, reagents, and instrumentation

Milli-Q water was used to prepare all aqueous solutions. All salts available from commercial sources were of analytical grade. Iron(III) chloride hexahydrate (# 236489), iron(II) sulphate heptahydrate (#215422), lead(II) nitrate (# 228621), manganese(II) sulphate monohydrate (#M7899), magnesium sulphate (# 746452), potassium nitrate (# 221295), potassium phosphate (# P5379), silver nitrate (# 209139), tetrakis (acetonitrile), copper(I) tetrafluoroborate (# 677892), tin(II) chloride (# 208256), and Trizma base (# T6066) were obtained from Sigma-Aldrich. Sodium chloride (# 54447) was from Warchem. Calcium nitrate tetrahydrate was from Chmes. Caesium carbonate (# C2160) was from TCI. Potassium tetrachloroplatinate(II) (# 11396315), zinc nitrate hexahydrate (# 10036103), aluminium nitrate nonahydrate (# 15269686), copper(II) nitrate trihydrate (#10124790), nickel(II) nitrate hexahydrate (# 10401651), iron(II) chloride (# 10401251), and sodium nitrate (# 11904281) were from ThermoScientific Acros. Potassium carbonate (# 427465708) and sodium sulphate (# 118078707) were from Chempur. Ammonium iron(II) sulphate hexahydrate (# 10087291), cobalt(II) nitrate hexahydrate (# 10529380), and lithium nitrate (# 10568620) were from Fisher Chemical. Cadmium nitrate tetrahydrate (# 11351579) and gallium nitrate monohydrate (# ALF-032116-14) were from Alfa Aesar. DMSO (# 83673.290) was from VWR. PBS (# 10010-015) and EDTA (# AM9260G) were from Thermo Fisher Scientific. Glycine (# GLN001) and ATP (# ATP007) were from BioShop Canada. BSA (# 160069) was from MP Biomedicals. NaOH (# 810981424) was obtained from POCH.

Positive controls for the luciferases activity assays are as follows: PT-C124 {3-[5-(2-fluorophenyl)-1,2,4-oxadiazol-3-yl]benzoic acid} (VWR # BIOV9421-10), isradipine (Tocris #2004), and N-benzyl-p-toluenesulfonamide BTS (VWR # J64910. X0). Luciferase enzymes for the activity assays were as follows: the firefly enzyme (Promega # E1701), NanoLuc enzyme (Promega # E499A), and Renilla enzyme (RayBiotech # RB-15-0003P-50). The substrates for the activity assays were beetle luciferin (Promega # E1603), furimazine (Aobious # AOB36539), and coelenterazine (Promega # S2001).

All bioluminescent signal generation experiments were performed in 384-well plate formats. The assay plates used were the LUMITRAC 384 (#781075) white from Greiner Bio-One. Multidrop Combi (Thermo Fisher) non-contact dispenser was used to add solutions of enzymes and substrate to the assay multi-well plates. Measurements of bioluminescent signal generation in the corresponding buffers were recorded with a CLARIOStar (BMG Labtech) plate reader. The pH measurements were carried out in vials with a Mettler Toledo™ FiveEasy FP20 pH metre.

### 2.2 Abbreviations

Abbreviations used throughout the text are listed alphabetically: N-benzyl-p-toluenesulfonamide (**BTS**), firefly luciferase enzyme (**FLuc**), glutathione (**GSH**), half maximal inhibitory concentration (IC_50_), HEPES buffer conditions (**H**), NanoLuc luciferase enzyme (**NLuc**), Ataluren (3-[5-(2-fluorophenyl)-1,2,4-oxadiazol-3-yl]benzoic acid (**PTC-124**), Renilla luciferase enzyme (**RLuc**), room temperature (**RT**), and screening conditions (**SC**).

All metal salts were abbreviated to their corresponding metal ion (e.g., Sn(II) for SnCl_2_). For several salts containing both the same metal ion and oxidation state (e.g., Fe(II), K(I), and Na(I)), differentiation was done by numeration. The following abbreviations have been used for the corresponding salts: AgNO_3_, **Ag**; Al(NO_3_)_3_·9H_2_O, **Al**; Ca(NO_3_)_2_·4H_2_O, **Ca**; Cd(NO_3_)_2_·4H_2_O, **Cd**; Co(NO_3_)_2_·3H_2_O, Co**(II)**; Cs_2_CO_3_, **Cs**; [Cu(CH_3_CN)_4_]BF_4_, **Cu(I)**; Cu(NO_3_)_2_·3H_2_O, **Cu(II)**; (NH_4_)_2_Fe(SO_4_)_2_·6H_2_O, **Fe(II)-1**; FeCl_2_, **Fe(II)-2**; FeSO_4_·7 H_2_O, **Fe(II)-3**; FeCl_3_·6H_2_O, **Fe(III)**; Ga(NO_3_)·H_2_O, **Ga**; KNO_3_, **K-1**; K_2_CO_3_, **K-2**; LiNO_3_, **Li**; MgSO_4_, **Mg**; MnSO_4_·H_2_O, **Mn(II)**; NaNO_3_, **Na-1**; NaCl, **Na-2**; Na_2_SO_4_, **Na-3**; Ni(NO_3_)_2_·6H_2_O, **Ni(II)**; Pb(NO_3_)_2_, **Pb(II)**; K_2_PtCl_4_, **Pt(II)**; SnCl_2_, **Sn(II)**; and Zn(NO_3_)_2_·6H_2_O, **Zn(II)**.

### 2.3 General procedure for luciferases activity assays in screening conditions (SC) and corresponding HEPES buffer conditions (H)

Screening assays were adapted from the established bioprofiling assay protocols from the European Chemical Biology Database (ECBD) (**European Chemical Biology Database**, https://ecbd.eu/; https://www.eu-openscreen.eu/services/bioprofiling-assays.html). All metal ion salt solutions as well as enzyme and substrate solutions were prepared freshly right before their transfer into 384-well plates. Positive controls used were PTC-124 (1 µM), isradipine (10 µM), and BTS (10 µM) for FLuc, NLuc, and RLuc, respectively, as reported in the literature (Cheng and Inglese, 2012; Auld and Inglese, 2016). The negative controls had the same %DMSO as the corresponding positive controls (0.04% for FLuc, 0.4% for NLuc, and 0.08% for RLuc).

#### 2.3.1 Firefly luciferase (FLuc) activity assay

Metal ion solutions in MilliQ water were transferred (5 µL) into 384-well plates. DMSO and PTC-124 were added as negative and positive controls to the corresponding columns of each assay plate to yield 0.1% and 1 µM final concentration, respectively. A Multidrop Combi dispenser was used to add 5 µL per well of 40 nM of stock solution of firefly luciferase enzyme in 1 mM Trizma base pH 7.6 buffer with 0.1 M glycine, 1 mM EDTA, and 10 mM magnesium sulphate (for SC) or in 200 mM HEPES (for H). Metal ion salts were pre-incubated with the enzyme for 10 min at RT. Then, the enzymatic reaction was initiated by the addition, with a Multidrop Combi dispenser, of 10 µL per well of 20 µM of the solution of beetle luciferin in either 0.5 mM Trizma base buffer pH 7.6 with 0.05 M glycine, 0.5 mM EDTA, 5 mM magnesium sulphate, 20 µM ATP, and 0.2% BSA (for SC) or 100 mM HEPES with 5 mM magnesium sulphate and 20 µM ATP (for H). For final concentrations and conditions, see Table 1. After 10 mins, luminescent signal was measured using a CLARIOStar plate reader.

**TABLE 1:** Final experimental conditions for each luciferase activity assay.

Enzyme	Substrate	Final buffer conditions		Positive controls
		Screening conditions (SC)	HEPES conditions (H)	
FLuc (10 nM)	D-luciferin (10 µM)	0.5 mM Trizma base, 0.05 M glycine, 0.5 mM EDTA, 5 mM MgSO_4_, 10 µM ATP, and pH 7.6	100 mM HEPES, 5 mM MgSO_4_, 10 µM ATP, and pH 7.5	PTC-124 (1 µM)
NLuc (10 pM)	Furimazine (10 µM)	PBS 0.75% × 0.1% BSA, and pH 7.4	100 mM HEPES, 0.1% BSA, and pH 7.5	Isradipine (10 µM)
RLuc (50 pM)	Coelentera-zine (2 µM)	50 mM Tris-HCl, 12.5 mM KH_2_PO_4_, 125 mM NaCl, 0.25 mM EDTA, 0.1% BSA, 0.3 M sodium ascorbate, and pH 7.4	100 mM HEPES, 0.05% BSA, 0.3 M sodium ascorbate, and pH 7.5	BTS (10 µM)

#### 2.3.2 Nano-luciferase (NLuc) activity assay

Metal ion solutions in MilliQ water were transferred (5 µL) into 384-well plates. DMSO and isradipine were added as negative and positive controls to the chosen corresponding columns of each assay plate to yield 0.1% and 10 µM final concentration, respectively. A Multidrop Combi dispenser was used to add 5 µL per well of 40 pM NanoLuc enzyme stock solution in either PBS 1x with 0.2% BSA (for SC) or in 200 mM HEPES with 0.2% BSA (for H). Metal ion salts were pre-incubated with the enzyme for 10 min at RT. Then, the enzymatic reaction was initiated by the addition of 10 µL per well of 20 µM of the solution of furimazine in either PBS with 0.1% BSA (for SC) or in 100 mM HEPES with 0.1% BSA (for H). For final concentrations and conditions, see Table 1. After 10 min, luminescent signal was measured using a CLARIOStar plate reader.

#### 2.3.3 Renilla luciferase (RLuc) activity assay

Metal ion solutions in MilliQ water were transferred (5 µL) into 384-well plates. DMSO and BTS were added as negative and positive controls chosen for the corresponding columns for each assay plate to yield 0.1% and 10 µM final concentration, respectively. A Multidrop Combi dispenser was used to add 5 µL solution of 200 pM Renilla enzyme in enzyme buffer of 50 mM potassium phosphate, 500 mM NaCl, and 1 mM EDTA at pH 7.4 with 0.2% BSA per well (for RLuc SC) or in 200 mM HEPES with 0.2% BSA per well (for RLuc H conditions). Metal ion salts were pre-incubated with the enzyme for 10 min at RT. Then, the enzymatic reaction was initiated by the addition of 10 µL coelenterazine 4 µM solution in the substrate buffer specific for standard screening conditions (SC substrate buffer: 0.1 M Tris-HCl pH 7.4 with 0.6 M sodium ascorbate per well) or in HEPES (H substrate buffer: 100 mM HEPES pH 7.5 with 0.6 M sodium ascorbate per well) with a Multidrop Combi dispenser. For final concentrations and conditions, see Table 1. After 10 min, the luminescent signal was measured using a CLARIOStar plate reader.

### 2.4 Experiment-specific variations in reagents concentrations and sequence of addition

#### 2.4.1 Initial screening experiments with metal ion salts in different buffer systems

FLuc, NLuc, and RLuc activity assays were carried out in screening conditions (SC) and in HEPES conditions (H) for 0.01, 1, and 5 mM of the final metal ion salt concentrations. The data were analysed with KNIME software.

#### 2.4.2 IC_50_ determination

FLuc, NLuc, and RLuc activity assays were carried out both in SC and in H with at least 11 concentrations of metal ion salts in the range 0.16–20 mM as the final concentrations. Every concentration of each metal was tested in triplicates on the same plate. In addition, if no top or bottom plateau was observed in the IC50 curve, additional experiments were performed for extended concentration ranges, and relative %INHIBITION values were combined on one figure for IC50 curve fitting. The data were analysed with KNIME software (see below).

#### 2.4.3 Reagents’ pre-incubation test

For the selected metal ion salts, namely, Ag, Cu(II), Fe(II)-1, Fe(II)-2, Fe(III), and Zn, three concentrations around the IC_50_ (top, middle, and bottom of plateau) and 1 mM were chosen as the final concentrations. Several pre-incubation tests were carried out with the same SC final conditions but with different orders of addition and longer incubation time (30 min instead of 10 min). **ATP + M then S:** FLuc assay with the subsequent addition of substrate and enzyme to a 30-min pre-incubated solution of ATP + metal. **E + M then S**: addition of a solution of substrate (+ATP in the case of FLuc) to a 30-min pre-incubated solution of metal + enzyme. **S + M then E**: subsequent addition of enzyme (and ATP in the case of FLuc assay) to a 30-min pre-incubated solution of substrate + metal (grey columns). The data were analysed with KNIME software.

#### 2.4.4 EDTA test

For the selected metal ion salts, namely, Ag, Cu(II), Fe(II)-1, Fe(II)-2, Fe(III), and Zn, three concentrations around the IC_50_ (top, middle, and bottom of plateau) and 1 mM were chosen as the final concentrations. EDTA testing was carried out in SC for NLuc with the final concentrations 0 and 0.5 mM and for RLuc with 0.25 and 0.5 mM. Corresponding EDTA concentrations for the EDTA–enzyme and EDTA–substrate solutions to be added to the well for NLuc were 4x (5 µL) and 2x (10 µL), respectively, whereas the corresponding EDTA concentrations for the EDTA–enzyme and EDTA–substrate solutions to be added to the well for RLuc were 2x (5 µL) and 1x (10 µL), respectively. The data were analysed with KNIME software.

#### 2.4.5 GSH test

For the selected metal ion salts, namely, Ag, Cu(II), Fe(II)-1, Fe(II)-2, Fe(III), and Zn, three concentrations around the IC_50_ (top, middle, and bottom of plateau) and 1 mM were chosen as the final concentrations. GSH testing was carried out with the final concentrations of 0, 0.2, and 2 mM, respectively, for FLuc, NLuc, and RLuc activity assays in SC. Corresponding GSH concentrations for the GSH–enzyme and GSH–substrate solutions to be added to the well were 4x (5 µL) and 2x (10 µL), respectively. The data were analysed with KNIME software.

### 2.5 Data analysis of luciferase activity assays

KNIME pipeline was generated and applied for the analysis of the results. The raw data files (one txt file per plate) were read and matched with plate template followed by calculation of averages and standard deviation of controls and compound wells, and Z′ factor values for each plate. The plates were proceeded in the loop, generating one dataset for all the tested plates. Automatic outlier removal was used for detecting control outliers for plates with Z′ factor values below 0.5. The plates for which removal of the outliers did not lead to improvement of the Z′ factor (higher than 0.5) or the algorithm was detecting more than two outliers were retested. The raw data values were normalised to positive and negative controls for each plate to calculate percent inhibition (% INHIBITION = (1-(x- μp)/(μn–μp))* 100%), Z' = 1- (3*(**SDp** + **SDn**)/(**μn** - **μp**)), signal to background ratio (**S/B** = **μn**/**μp)**, variance coefficient (CV = SD of population/mean *100%), percent normalised bioluminescent signal (**%** N_VALUE = (**x**/**μn**)*100), and normalised bioluminescence signal change (% N_SCh = (x - μn)/μn*100), where **x** represents the readout **VALUE** of the well, **μp** represents the mean of the positive controls, **μn** represents the mean of the negative controls, **SDn** represents the standard deviation of the negative controls, and **SDp** represents the standard deviation of the positive controls for each tested assay plate. The heatmap of % INHIBITION was then generated for each plate in order to identify plate effects or signal distribution patterns. Additionally, to further test for plate effects, a validation plate (no metal ions added) was run for each assay under the screening conditions.

From the data table, the compound’s concentration, % inhibition, and compound ID were selected, and using a pipeline in KNIME and a node that utilises R programming, dose–response curves were generated for each compound. All curves were generated using the four-parameter logistic (LL.4) regression model. The IC50 values were determined from the inflection points of the generated curves. The quality control of curve fitting was performed by 1) a visual assessment of the plotted curves; 2) an evaluation of the coefficient of the determination parameter, **R-squared**, calculated according to equation **R** ^**2**^ **= 1 - SS**_**res**_ **/SS**_**total**_, where **SS**_**res**_ is the residual sum of squares (Y_i_ measured - Y_i_ predicted)^2^ and **SS**_**total**_ is the total sum of squares (Y_i_ measured - Y mean)^2^; and 3) the residual standard error parameter, **RSE**, calculated according to equation **RSE = sqrt(SS**_**res**_ **/df**_**res**_ **)**, where **SS**_**res**_ is defined as above and **df**_**res**_ are residual degrees of freedom **df**_**res**_ **= n - k** (**n** is the number of observations and **k** is the number of parameters estimated). These are reported in Supplementary Figure S4 and Supplementary Table S1.

All raw data as well as platemaps and calculated QC parameters are provided in Supplementary Tables 1, 2 spreadsheet files attached in the Supplementary Material.

### 2.6 pH measurements

#### 2.6.1 General procedure for measuring the pH of metal ions in SC conditions for FLuc, RLuc, or NLuc

The pH of the screening conditions’ buffer (SC, 1 mL) of FLuc (0.5 mM Trizma base, 0.05 M glycine, 0.5 mM EDTA, 5 mM MgSO_4_, 10 µM ATP, and pH 7.6), NLuc (PBS 0.75% × 0.1% BSA, and pH 7.4), or RLuc (50 mM Tris-HCl, 12.5 mM KH_2_PO_4_, 125 mM NaCl, 0.25 mM EDTA, 0.1% BSA, 0.3 M sodium ascorbate, and pH 7.4) was measured, followed by the addition of appropriate volumes of 0.2 M aqueous stock solutions of selected metal ion salts (Ag, Cu(II), Fe(II)-1, Fe(II)-2, Fe(III), and Zn) to achieve the final concentration as indicated by x, y, and z in Table 2. The obtained solutions were stirred, and the pH was measured. Finally, second sets of aliquots were added to the same vials to reach a final concentration of 5 mM for all salts (except Zn in SC RLuc buffer, to 10 mM), and the pH was measured again. These experiments were performed in triplicates.

**TABLE 2:** Concentrations (mM) of each metal ion at their respective addition steps (x, y, z, z1, x2, and x3).

Buffer	Variable	Ag	Cu(II)	Fe(II)-1	Fe(II)-2	Fe(III)	Zn
SC FLuc	x	0.0005	0.2	1	0.02	0.5	2.5
SC NLuc	y	0.05	0.02	n/a	2.5	2.5	n/a
SC RLuc	z	0.2	0.2	0.5	n/a	0.5	5
HEPES	x1	0.0001	0.0001	0.1	0.025	0.1	0.025
HEPES	x2	0.025	0.025	0.5	0.5	1	1
HEPES	x3	0.1	0.1	2.5	n/a	2.5	2.5

Concentration of metal ions before addition was 0 mM, and after a final addition, it was 5 mM for all metal ions and buffers, except Zn in SC RLuc buffer, where the final concentration was 10 mM.

#### 2.6.2 General procedure for measuring pH during the titration experiment of metal ions into 100 mM HEPES buffer

To 5 mL of 100 mM HEPES buffer, aqueous stock solutions of metal ion salts (0.2 M, Ag, Cu(II), Fe(II)-1, Fe(II)-2, Fe(III), and Zn) were added in four incremental steps into the same vial. The volume added at each step was equal to the volume required to achieve the concentration as indicated in Table 2 (x1, x2, and x3), with the final addition leading to the concentration of 5 mM. The pH was measured upon stirring before and after each addition, and the experiment was repeated in triplicates.

## 3 Results

### 3.1 HTS screening for metal ions’ interference in luciferase-mediated bioluminescent signal

#### 3.1.1 Assay development and quality control parameters

Initial screening experiments of FLuc, NLuc, and RLuc activities were performed according to the established protocols used in screening, in buffers of the same pH (see Table 1, labelled as SC—screening conditions), but based on glycine/TRIS, phosphate, and a combination of Tris and phosphate, respectively. Each of the buffers also contained additional elements that are either critical (e.g., Mg and ATP that act as key co-factors and reagents in bioluminescence production by FLuc assay) or recommended additives (e.g., EDTA for FLuc and RLuc; BSA and sodium ascorbate for NLuc and RLuc). To eliminate some of the variability in buffer systems, all three assays were also transformed into their HEPES buffer-based variants (Table 1; labelled as H) of the same near-neutral pH suitable for optimal luciferases’ activity and high enough buffer concentration (100 mM) to ensure sufficient buffering capacity, even for high concentrations of metal ions. The aim of this unified buffer system was to create a possibility of a more direct comparison between luciferases. However, the removal of some of the additives led to a lack or very little signal. Therefore, both ATP and Mg still need to be present in the FLuc reaction buffer based on HEPES, whereas both NLuc and RLuc required the presence of BSA, and RLuc also needed sodium ascorbate (Table 1).

**FIGURE 1:**
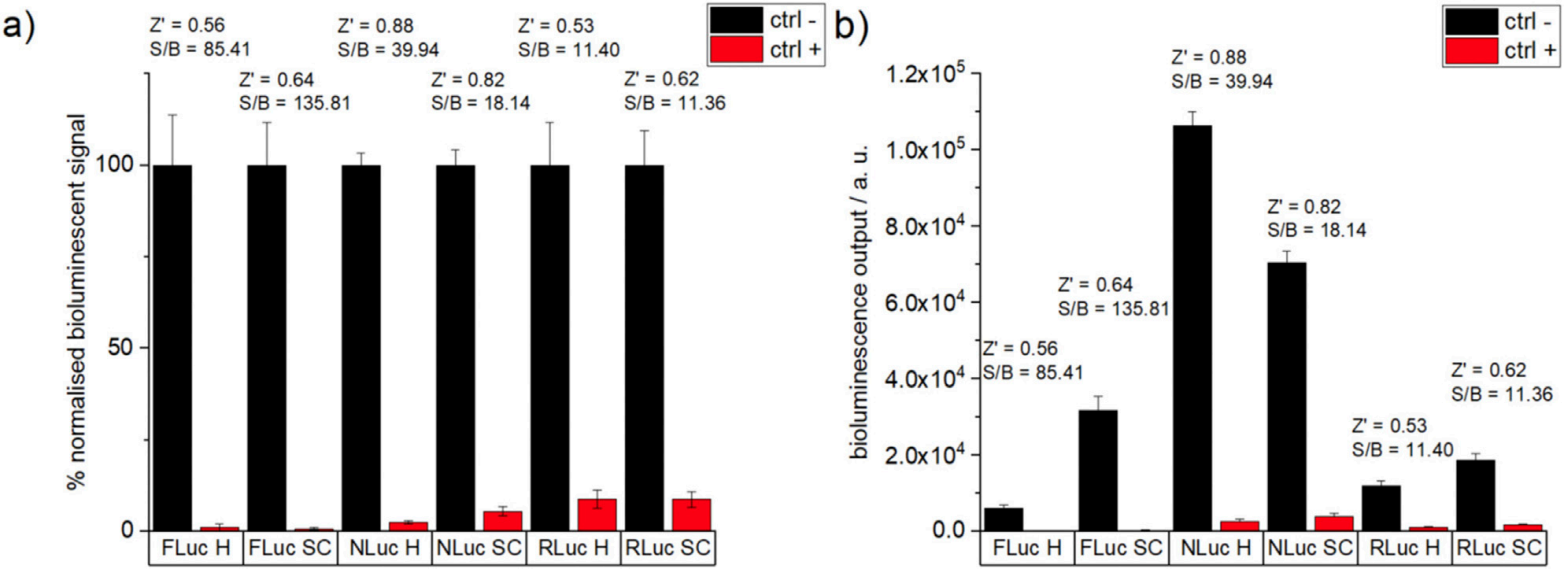
Relative bioluminescent signal **(A)** and bioluminescent output value **(B)** of positive (red) and negative (black) controls from assays for each luciferase–luciferin pair. Positive controls used are PTC-124 (1 µM), isradipine (10 µM), and BTS (10 µM) for FLuc, NLuc, and RLuc, respectively. The negative controls had the same %DMSO as the corresponding positive controls. Assay quality parameters (Z′ factor, S/B ratio: calculated according to the equations in Section 2.5). Each condition for a given type of experiment was performed in triplicate on the same plate, but every sample is prepared independently and each experiment type was performed on a different day.

In order to ensure suitable assay performance, PTC-124 (1 µM), isradipine (10 µM), and BTS (10 µM) inhibitors were used as positive controls, respectively, for FLuc, NLuc, and RLuc activity assays. Based on the experiments with and without inhibitors, a common quality-control HTS assay parameter, Z’ factor, for all six assays (three luciferases, each in two buffer systems) remained above 0.5, which is a recommended minimal value for biochemical assays (Figure 1A). The highest Z’ factor value of 0.88 was observed for the NLuc assay in HEPES (H) conditions vs. 0.82 in SC. Reversely, for FLuc and RLuc, the assays in SC exhibited slightly higher Z’ factor values (respectively, for FLuc assays: 0.64 for SC vs. 0.56 for H, and for RLuc assays: 0.62 in SC vs. 0.53 in H).

Additionally, the highest signal-to-background ratios (S/B—the ratio between bioluminescent signal for the negative control vs. bioluminescent signal for the positive control) were measured for FLuc assays (S/B 136 in SC and 85 in H), and S/B values were slightly lower for NLuc assays (40 for H and 18 for SC) followed by RLuc assays (S/B = 11 for both SC and H). This is in line with the strongest relative effectiveness of signal quenching by the inhibitors in FLuc assays (lowest relative residual bioluminescence from positive control, red in Figure 1A). Despite lower absolute values of residual bioluminescence in the presence of inhibitors in RLuc assays in comparison to NLuc (red in Figure 1B), the highest absolute bioluminescence intensity of the positive control for the NLuc assay (black in Figure 1B) leads effectively to the lower relative residual bioluminescence in NLuc assays vs. RLuc ones (red in Figure 1A) and is the main contributor to high S/B and Z’ factor parameters of NLuc assays.

Importantly, validation plate runs with screening conditions (no addition of metal ions or inhibitors) showed no particular pattern of the signal distribution across the plate (Supplementary Figure S1). Similarly, no plate position-dependence of the signal other than that related to the difference in the experimental conditions (no pattern on a single plate or between plates) was observed for the positive and negative controls in heatmaps for all plates (Supplementary Figure S2). All of this confirms the robustness of the assays and the lack of position-dependent artefacts, enabling reliable data generation independent of the plate design.

#### 3.1.2 Selection of metal ion salts

For initial screening experiments, 26 salts of 21 different biologically and environmentally relevant metal ions were selected. Most of the salts were in the form of highly soluble nitrates, with iron(II) (Fe(II)-2), iron(III) (Fe(III)-3), sodium (Na-2), and tin(II) (Sn (II)) as chlorides; iron(II) (Fe(II)-1 and 3), magnesium (Mg), manganese(II) (Mn(II), and sodium (Na-3) as sulphates; potassium (K-2) and cesium (Cs(I)) as carbonates; and copper(I) (Cu(I)) as a tetrafluoroborate. In order to get insight into the ionic strength and/or anionic effects, three different salts of sodium (sodium nitrate,Na-1; sodium chloride, Na-2; and sodium sulphate, Na-3), two different salts of potassium (potassium nitrate, K-1 and potassium carbonate, K-2), and three different salts of iron(II) (ammonium iron(II) sulphate hexahydrate, Fe(II)-1; iron(II) chloride, Fe(II)-2; and iron (II) sulphate, Fe(II)-3) were also used. The aqueous solutions of three of the selected metal ions (Fe(II), Fe(III), and Cu(I)) were particularly unstable, and they needed to be prepared freshly every time and used immediately after dissolution in water/buffer. In particular, in near-neutral pH, Fe(III) tends to form various water insoluble oxides that precipitate out of the solution in time and/or at higher concentrations, leading to a change in effective metal concentration that cannot be easily estimated. Fe(II) and Cu(I) are also particularly susceptible to oxidation even in air, particularly in a solubilised form, and so they needed to be handled with care and just for short periods of time. In addition to having high oxidation susceptibility, most of the widely available Cu(I) salts remain rather insoluble in aqueous solutions. Therefore, following the literature standards, the acetonitrile Cu(I) complex was used as a source of Cu(I). The salt is relatively stable in the solid form and is highly soluble in acetonitrile that stabilizes the Cu(I) state. After subsequent dilution of acetonitrile stock solutions of Cu(I) in water, the ion becomes highly susceptible to oxidation (and partial or total ligand exchange to water can occur); therefore, Cu(I) aqueous solutions were freshly prepared every time and measured immediately after addition to the reaction buffer.

#### 3.1.3 Initial screening for metal ion effects on the bioluminescent signal of FLuc

**FIGURE 2:**
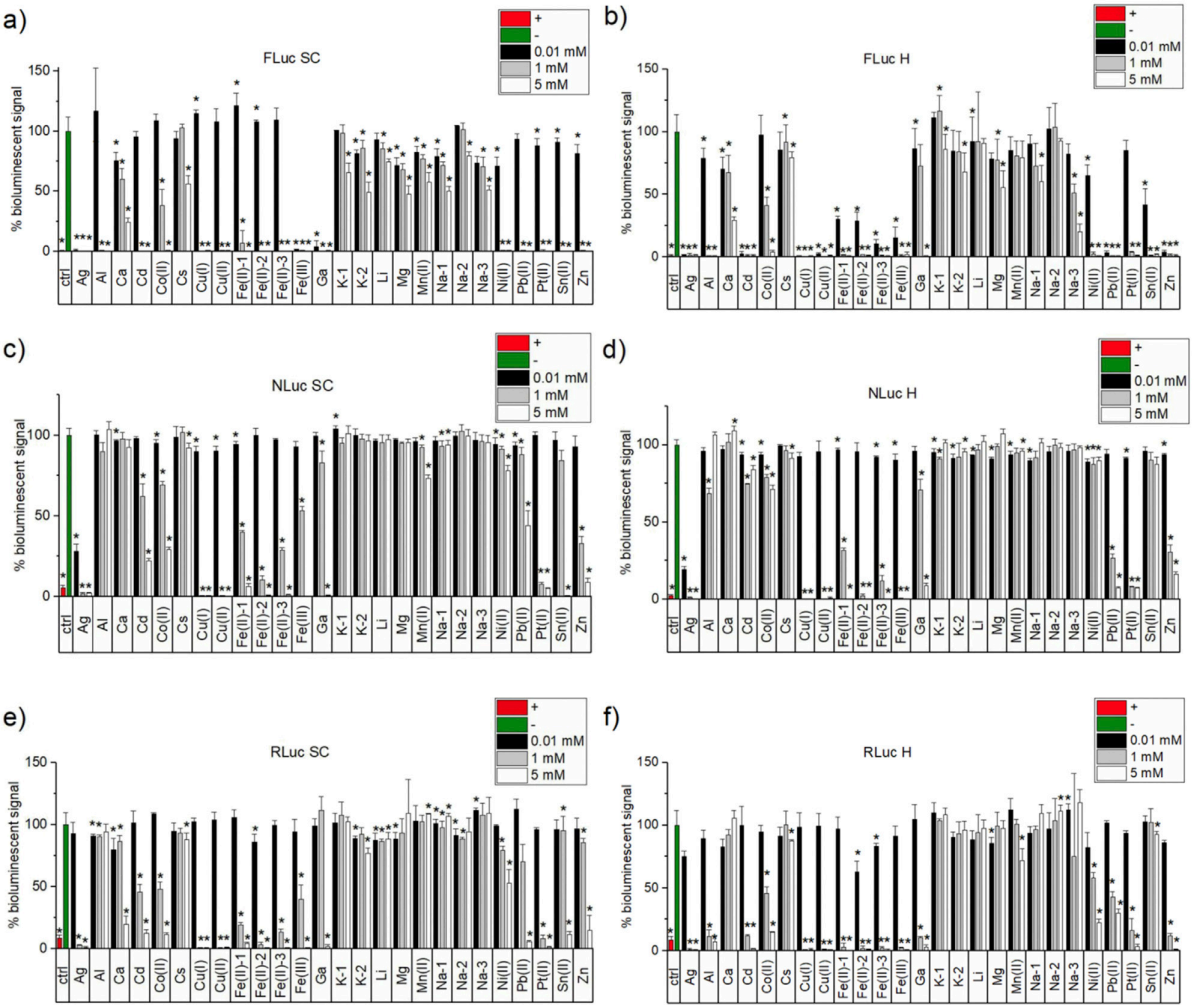
Normalised bioluminescent signal in the presence of three different concentrations (0.01. mM, black; 1 mM, light grey; 5 mM, white) of metal ion salts in optimised luciferase activity assays of **(A)** FLuc SC (10 µM substrate, 10 nM enzyme, 25 mM glycine, 0.25 mM EDTA, 2.5 mM MgSO_4_, 10 µM ATP, and pH 7.6), **(B)** FLuc H (100 mM HEPES, 5 mM MgSO_4_, 10 µM ATP, and pH 7.5) **(C)** NLuc SC (10 µM substrate, 10 nM enzyme, PBS pH 7.4 0.75% 0.1% BSA) **(D)** NLuc H (100 mM HEPES, 0.1% BSA, and pH 7.5), **(E)** RLuc (2 µM substrate, 50 pM enzyme, 50 mM Tris-HCl, 12.5 mM KH_2_PO_4_, 125 mM NaCl, 0.25 mM EDTA, 0.1% BSA, and 0.3 M sodium ascorbate), and **(F)** RLuc H (100 mM HEPES, 0.05% BSA, 0.3 M sodium ascorbate, and pH 7.5). Positive controls (red) used are PTC-124 (1 µM), isradipine (10 µM), and BTS (10 µM) for FLuc, NLuc, and RLuc, respectively. The negative controls (green) had the same %DMSO as the corresponding positive controls.Each condition for a given type of experiment was performed n = 3 on the same plate, but every sample was prepared independently and each experiment type was performed on a different day. Normalisation of the bioluminescent signal was carried out against the negative control output of the corresponding bioluminescent assay plate. Statistical two-tailed Student’s t-test (95% confidence) was carried out between a given sample and the corresponding negative control. The samples were deemed statistically different from the negative control for *p* < 0.05 and were marked with black “*.”

As reported above in Chapter 2, all the selected metal ions have been tested initially in three assays, each in previously optimised screening conditions (SC) at three different salt concentrations, 0.01 mM (imitating a standard concentration of drug candidates used in screening), 1 mM, and 5 mM (equal to the highest concentration of Mg ions used in the standard FLuc SC buffer). The results of these experiments are presented in Figure 2 (each panel corresponds to a different assay) and Supplementary Figure S3 (plots responses of different luciferases to the same metal ion salts’ concentrations against each other). Importantly, in the FLuc assay, in the screening conditions buffer (SC, Figure 2A), all metal ion salts, with the exception of Al, exhibited a statistically significant effect (quenching) on the bioluminescent signal (“*” in Figure 2A), with Cd, Co(II), Cu(I), all iron salts, Ga, Ni(II), Pb(II), Pt(II), Sn(II), and Zn, inducing almost complete quenching at that concentration and at 1 mM (with the exception of Co(II)). For Ag, Fe(III), and Ga, the bioluminescent signal was almost completely quenched even at 0.01 mM concentration (respectively, 1.1%, 1.3%, and 3.8% of residual signal remained; Table 3; Figure 1A), with lower but statistically significant quenching effect (between 71.2% and 90.8% of residual signal) noticeable for Al, Ca(II), K-2, Mg, Mn(II), Na-1, Ni(II), Pt(II), Sn(II), and Zn(II).

**TABLE 3:** Summary of the effect of metal ion salts on bioluminescent signal (residual % signal after the addition of metal, 100%: no effect, 0%: full quenching) in screening buffer and in HEPES buffer for FLuc, NLuc, and RLuc.

Metal ion	Counter ion	FLuc SC			NLuc SC			RLuc SC			FLuc H			NLuc H			RLuc H		
		0.01 mM	1 mM	5 mM	0.01 mM	1 mM	5 mM	0.01 mM	1 mM	5 mM	0.01 mM	1 mM	5 mM	0.01 mM	1 mM	5 mM	0.01 mM	1 mM	5 mM
Ag	NO_3_^−^	1.1	0.3	0.4	28.0	2.0	2.1	92.8	2.7	0.9	1.4	1.4	1.5	19.6	1.0	0.2	75.3	1.0	0.5
Al	NO_3_^−^	116.7	0.6	0.5	100.4	89.9	103.4	91.1	90.2	94.2	78.9	0.7	0.5	95.9	68.7	106.5	89.3	11.2	7.2
Ca	NO_3_^−^	75.6	60.2	24.5	96.5	97.6	92.6	79.9	86.7	19.5	70.0	67.2	29.2	97.1	101.8	109.2	83.0	92.3	105.8
Cd	NO_3_^−^	95.2	0.3	0.2	98.2	62.1	22.1	101.5	45.6	12.2	2.4	1.1	1.1	93.8	74.6	84.1	100.1	11.8	1.4
Co(II)	NO_3_^−^	109.0	38.5	0.5	95.1	69.0	29.4	108.5	47.9	11.5	97.6	41.1	3.8	93.8	78.9	71.3	94.8	45.7	15.0
Cs	CO_3_^2-^	93.8	103.0	56.2	98.8	101.9	92.2	94.9	93.0	87.8	85.2	91.6	79.4	99.4	96.5	91.4	91.5	100.3	87.4
Cu(I)	BF_4_^−^	115.0	0.5	0.4	90.0	0.8	0.4	102.5	0.5	0.6	0.5	0.4	0.7	92.6	0.3	0.1	98.7	0.8	1.1
Cu(II)	NO_3_^−^	107.7	0.7	0.6	90.4	0.7	0.2	103.6	0.6	0.9	2.6	0.4	1.0	95.5	0.2	0.7	99.3	0.8	0.8
Fe(II)-1	NH_4_^+^, SO_4_^2-^	121.4	6.6	0.2	94.4	40.0	6.3	105.8	19.0	4.2	30.1	1.5	0.7	96.7	31.7	0.4	97.0	2.9	0.5
Fe(II)-2	Cl^−^	107.9	0.3	0.4	99.7	10.5	0.8	85.9	3.5	0.4	28.9	1.8	0.8	95.6	2.4	0.2	62.9	1.9	0.8
Fe(II)-3	SO_4_^2-^	109.4	0.2	0.2	97.1	29.0	1.1	99.5	13.5	0.8	10.5	1.1	1.2	92.1	12.2	0.3	83.1	2.3	0.7
Fe(III)	Cl-	1.3	0.7	0.3	93.0	53.4	0.2	94.1	39.9	0.8	15.5	1.1	1.9	90.2	0.5	0.2	91.5	2.3	1.0
Ga	NO_3_^−^	3.8	0.3	0.5	99.5	83.0	0.6	98.9	111.7	1.9	86.3	72.6	0.7	96.1	70.9	8.8	104.9	10.6	3.0
K-1	NO_3_^−^	100.2	98.6	65.5	104.1	94.9	101.1	101.3	107.6	102.4	111.3	116.3	86.1	95.2	90.9	101.3	110.1	103.8	108.7
K-2	CO_3_^2-^	81.3	85.8	49.2	100.0	97.6	96.6	88.9	92.2	76.8	84.3	83.9	67.9	91.3	92.1	95.6	90.4	93.2	96.4
Li	NO_3_^−^	93.0	85.8	74.5	96.7	95.4	97.1	87.4	86.8	88.5	92.2	92.2	90.5	93.2	97.0	102.3	88.6	94.2	97.7
Mg	NO_3_^−^	71.5	68.0	47.7	97.4	95.1	95.5	88.5	93.1	109.0	78.3	77.4	55.5	91.0	99.0	107.1	85.7	99.3	97.7
Mn(II)	NO_3_^−^	82.6	77.1	57.4	96.1	92.7	73.4	102.9	102.5	108.6	85.0	80.6	79.4	93.9	95.0	96.0	112.4	101.0	71.9
Na-1	NO_3_^−^	79.1	71.7	50.0	96.7	93.1	94.1	101.1	97.6	106.5	90.0	72.4	60.0	89.7	91.8	101.6	93.9	96.7	109.5
Na-2	Cl^−^	104.6	101.5	79.7	99.4	102.5	99.4	91.3	88.6	94.4	102.1	103.4	92.4	95.5	99.8	98.5	97.4	103.6	111.2
Na-3	SO_4_^2-^	73.4	70.4	51.1	96.8	96.0	95.6	111.5	107.5	109.3	81.9	51.0	20.0	96.0	96.8	98.4	112.2	75.1	118.1
Ni(II)	NO_3_^−^	71.2	0.5	0.6	94.5	91.4	78.1	99.1	79.3	52.7	64.7	2.3	0.8	89.0	87.6	89.9	82.5	57.9	22.4
Pb(II)	NO_3_^−^	93.3	0.7	0.3	93.8	88.2	44.2	112.5	69.9	6.0	3.1	0.8	1.0	94.0	26.7	7.6	101.9	42.9	30.2
Pt(II)	K^+^, Cl^−^	87.9	0.7	0.3	99.8	7.8	5.1	96.1	8.1	1.2	85.0	3.8	1.1	91.5	8.1	7.5	93.6	16.3	3.4
Sn(II)	Cl^−^	90.8	0.4	0.6	97.0	84.2	0.4	96.3	95.3	11.3	41.7	1.2	1.9	95.8	90.1	87.3	102.7	102.5	92.9
Zn	NO_3_^−^	81.5	0.8	0.3	93.0	32.8	9.0	96.8	85.7	14.9	3.8	1.5	1.1	93.6	30.5	16.1	86.0	11.8	1.1

Colour range: red, below 10%; orange, 10%–50%; yellow, 50%–80%; and non-coloured, above 80%.

Positive controls used are as follows: PTC-124 (1 µM), isradipine (10 µM), and BTS (10 µM) for FLuc, NLuc, and RLuc, respectively. The negative controls had the same %DMSO as the corresponding positive controls.

Each condition for a given type of experiment was performed in triplicate on the same plate, but every sample was prepared independently and each experiment type was performed on a different day. Values are averaged over three repeats and measured relatively to plate-specific positive and negative controls.

In HEPES buffer conditions (Table 3; Figure 1B), quenching was generally more pronounced in all concentrations, and at 0.01 mM, in addition to Ag and Fe(III) salts that did have that effect in SC, Cd, Cu(I), Cu(II), all iron(II) salts, Pb(II), and Zn also led to almost full disappearance of bioluminescence (strong quenching). The opposite, that is, higher relative bioluminescent signal (lower signal quenching effect) in H over SC for FLuc, was observed for Ga (significant inhibition at 5 mM but not so much at 1 mM and 0.01 mM in H); all monovalent cations except Cu(I), Ag, and Na-3 with sulphate cation (Li, Na-1, and Na(II)-2, all K salts, and Cs all exhibiting approximately 15%–30% more signal in H vs. SC); and Mg (47.7%–55.5% from SC to H) and Mn(II) (57.4%–79.4% for SC vs. H—Table 3).

Clear trends emerge when directly comparing the relative residual bioluminescent signals for different luciferases at the same metal ion salts’ concentrations and buffers (Supplementary Figure S3). In particular, in SC conditions, at the lowest concentration (0.01 mM), RLuc activity is largely unaffected, with NLuc being partially quenched by Ag and FLuc almost completely quenched by Ag, Fe(III), and Ga (Supplementary Figure S3A). However, all luciferases are largely quenched by all metal ion salts at 5 mM concentration at the SC condition (Supplementary Figure S3E) and also mostly quenched in the H conditions, except Cd(II) and Co(II) for NLuc and Sn(II) for both NLuc and RLuc (Supplementary Figure S3F). Interestingly, at the H condition, FLuc is quenched by all metal ions even at the lowest concentration of 0.01 mM, except Co(II), Ga, and Pt(II), whereas RLuc is significantly affected mainly by Ag and Fe(III) and very slightly by Zn (Supplementary Figure S3B). NLuc bioluminescence in H conditions, on the other hand, is only mildly affected at 0.01 mM salts’ concentration, with the exception of strong quenching by Ag (Supplementary Figure S3B).

#### 3.1.4 Initial screening for metal ion effects on the bioluminescent signal of NLuc and RLuc

For NLuc and RLuc assays in SC (Table 3; Figures 2C, E respectively), little to no effect was observed for any of the metal ions at 0.01 mM, with the exception of Ag in the NLuc assay. Very similar trends (and similar types of metal ions) were observed for both at higher concentrations, with almost complete quenching observed for Ag, Cu(I), Cu(II), Fe(II)-2, and Pt(II) at 1 mM and 5 mM concentrations and Ga(II), Fe(II)-1, and Fe(II)-3, Fe(III), Pb(II), Sn(II), and Zn at 5 mM. For Cd, Co(II), and Ni(II), quenching in the NLuc SC assay was less pronounced than that in the RLuc one, and the effect was even more clearly visible at 5 mM concentration for Pb(II) (only 6% residual signal in RLuc vs. 44.2% in NLuc) and especially Ca(II) (19.5% in RLuc vs. 92.6% in NLuc SC assays).

In analogous assays with NLuc and RLuc, but in HEPES buffer conditions (Table 4; Figure 2C vs. Figure 2D for NLuc; Figure 2E vs. Figure 2F for RLuc), the effects are largely the same. The loss of the inhibitory effect in H vs. SC at even 5 mM concentrations is observed for Sn(II) on RLuc and NLuc and for Ca(II) in the RLuc H assay. More pronounced quenching (in H vs. SC), on the other hand, is observed in the RLuc assay for Ga at 1 mM (no quenching in SC vs. only 10.6% residual signal remaining in H). Finally, the opposite effects of changing the buffer from SC to H when comparing both luciferases are observed for Cd and Co(II) (weakened for NLuc but more pronounced for RLuc) as well as Pb(II) (weakened for RLuc but exacerbated for NLuc).

**TABLE 4:** IC_50_ values from the curve fitting for each luciferase activity assay in SC and in H.

Metal ion	FLuc SC IC_50_/µM	FLuc H IC_50_/µM	NLuc SC IC_50_/µM	NLuc H IC_50_/µM	RLuc SC IC_50_/µM	RLuc H IC_50_/µM
Ag	0.24	<0.01*	4.50	46.63*	68.44	26.98
Cd	568.55	1.21	1633.95	88.31	536.75	294.56
Co(II)	726.99	31.54	3996.84	>5000	688.50	>5000
Cu(I)	408.12	0.016 < IC50 < 0.063*	46.64	56.45	363.64	18.51
Cu(II)	125.35	0.07	31.08	22.38	132.36	7.79
Fe(II)-1	180.67	23.76	294.34	336.44	107.62	43.08
Fe(II)-2	264.05	22.89	883.48	364.32	117.53	40.47
Fe(II)-3	256.96	2.73	438.79	316.77	114.89	47.27
Fe(III)	23.91	22.32	816.32	130.01	535.61	62.54
Ga	14.94*	10.54	1815.21	2125.75	4285.96	397.97
Pb(II)	737.41	2.74	>5000	541.60	2558.78	598.29
Pt(II)	42.97	39.93	258.81	246.10	350.43	723.68
Sn(II)	85.45	>5000*	2099.27	>5000*	>5000	>5000*
Zn	1200.65	0.88	106.40	128.18	4268.64	196.40

For samples marked in red and with “*” in their IC50 value, the fitting is deemed unsuitable (R2 below 0.80 generally; and no fitting possible for FLuc H Cu(I)).

### 3.2 Concentration-dependent inhibitory effects of selected active metal ions (IC_50_ determination)

Following the initial screening of metal ions’ effects in six assays, those that have exhibited significant inhibitory influence were selected for a follow-up investigation toward quantification of their IC_50_ (50% inhibitory concentrations). This included the following salts: Ag, Cd, Cu(I), Cu(II), Fe(II)-1,2 and 3, Fe(III), Ga, Pb(II), Pt(II), Sn(II), and Zn. For each of the salts, measurements were performed for at least 11 different concentrations (ranging from 0.16 nM to 20 mM as the final concentrations) in triplicates. The results of the experiments have been subsequently plotted on the logarithmic scale graph (Supplementary Figure S1). Subsequently, data were fit with the LL.4 regression model, and the IC_50_ values (concentration inducing 50% inhibition) were extracted and re-summarised in Table 4. Parameters characterising the corresponding curves (including slope and lower and upper limit) have been reported in Supplementary Table S1. For several instances, including Sn(II) in particular for all assays but FLuc in SC, Co(II) for NLuc H and RLuc H, and Pb(II) for NLuc SC conditions, 100% inhibition was not achieved even at the highest concentrations, leading to an inadequate fit and no reliable IC_50_ estimation, indicating weak to no inhibition. In the case of Ag and Cu(I) salts in the FLuc H assay, the inhibition was very significant even at the highest concentration, leading to the lack of reliable IC_50_ fit but clearly indicating that the value of that parameter lies below 0.1 µM. Further examination of the IC_50_ and the curves largely confirms qualitative observations from the initial screen.

In general, the influence of metal ions is more pronounced in FLuc assays, with the following order of the strength of inhibition according to IC_50_ values (the lower the IC_50_ value, the stronger the inhibitor), starting from the strongest, for the H conditions: Ag(I), Cu(I) (0.01 µM) > Cu(II) (0.07 µM) > Zn (0.88 µM) > Cd (1.21 µM) > Fe(II)-3 (2.73 µM) and Pb(II) (2.74 µM) > Ga (11 µM) > Fe(II)-1, Fe(II)-2, and Fe(III) (all between 22–24 µM) > Co(II) (31 µM) > Pt(II) (40 µM). The IC_50_ values in the SC conditions for the FLuc assay are in the same order of magnitudes for Fe(III), Ga, and Pt(II); only slightly higher (weaker inhibition) for Ag (0.24 µM in SC); one order of magnitude higher for Co(II), Fe(II)-1, and Fe(II)-2; two orders of magnitude higher for Fe(II)-3, Pb(II), and Cd; more than three orders of magnitude larger for Zn and Cu(II); and over 10,000 times larger for Cu(I).

In the case of NLuc assays, only Ag in the SC buffer has an IC_50_ value below 10 µM (4.5 µM); Ag in the H buffer and Cu(I) and Cu(II) in both H and SC buffers exhibit IC_50_ values between 22 µM and 56 μM, with the rest of the metal ions’ IC_50_ being above 100 µM in both H and SC, and above 1 mM in case of Cd, Co(II), Ga, and Sn(II) in both SC and H and Pb(II) in SC conditions. For RLuc assays, the strongest inhibition is seen for Cu(II) ions in the H condition (7.79 µM), with Cu(I) and all iron salts in the H buffer and Ag in both H and SC being within the 18–68 µM range. Ga(II) in SC, Pb(II) in SC, Zn in SC, Co(II) in H, and Sn(II) in both buffers have all shown IC_50_ values of above 1 mM. As for NLuc, higher inhibition can be found generally in the H conditions, with Ag being approximately 2 times less inhibiting in SC than in H, with Cu(I) and all iron as well as Ga, Pb(II), and Zn salts having one order of magnitude higher IC_50_ values in SC than H. Co(II) (688 µM in SC) and Pt(II) (350 µM in SC vs. 723 µM in H) are the only metal ions with higher inhibitory effect in SC than in H for RLuc assays, but even in SC, the effect is moderate to weak.

### 3.3 Effects of other interferents and buffer elements on metal ion-induced changes in luciferases’ activities

On the basis of the experiments above, a handful of the most potent and biologically relevant metal ions were selected to further investigate the nature of the potential interference of metal ions with bioluminescent signal generation in luciferase activity assays. This included Ag (highest potency across the board), Cu(II), Fe(III) (biological relevance, significant potency, and high abundance in environment as well as impurity), and two Fe(II) salts to study, along with the counter-ion effects, and Zn due to its biological and environmental ubiquity. All of the experiments below will therefore involve only these selected metal ions.

#### 3.3.1 pH measurements for buffers upon the addition of metal ions

Screening buffers for each of the luciferases differ by the chemical nature of the buffer as well as its concentration, and therefore its buffering capacity. In order to account for pH change as the potential source of inhibitory effects of metal ions on luciferases’ activity, pH measurements of metal solutions in different concentrations were performed for standard screening buffers and HEPES (100 mM). To study the effect in more depth, the experiment had the form of a titration in which 0.2 M stock solutions of the selected metal ions were added to the buffer of interest, the solution was stirred, and the pH was measured. The results of those experiments are provided in Table 5 below. The final maximal concentration of selected metal ions in each of the buffers was 5 mM (the highest concentration used in the screening, with the exception of 10 mM for Zn in RLuc SC buffer), whereas the intermediate concentrations were adjusted to match the lowest inhibitory concentration of the metal ion against the respective luciferase (Table 2).

**TABLE 5:** Result of pH titration experiments (with standard deviations, n = 3) of various metal ions in SC conditions and in H conditions for different concentrations (see Table 2 for concentrations of x, y, and z and x1, x2, and x3).

		C [mM]	Ag	Cu(II)	Fe(II)-1	Fe(II)-2	Fe(III)	Zn
SC	FLuc	**0**	6.95 ± 0.03	6.94 ± 0.10	6.94 ± 0.06	6.92 ± 0.07	6.88 ± 0.09	6.89 ± 0.08
		**x**	6.92 ± 0.03	6.90 ± 0.06	6.93 ± 0.03	6.96 ± 0.05	6.64 ± 0.05	5.39 ± 0.06
		**5**	5.97 ± 0.18	3.53 ± 0.07	5.75 ± 0.02	5.79 ± 0.11	3.29 ± 0.08	5.05 ± 0.05
	NLuc	**0**	7.27 ± 0.02	7.33 ± 0.06	7.32 ± 0.08	7.32 ± 0.10	7.33 ± 0.08	7.24 ± 0.02
		**y**	7.28 ± 0.02	7.24 ± 0.01	-	6.73 ± 0.05	5.74 ± 0.06	-
		**5**	7.30 ± 0.04	4.89 ± 0.03	6.19 ± 0.06	6.28 ± 0.07	2.71 ± 0.04	5.55 ± 0.02
	RLuc	**0**	7.02 ± 0.01	6.99 ± 0.04	7.04 ± 0.03	7.07 ± 0.06	7.04 ± 0.06	7.03 ± 0.03
		**z**	7.02 ± 0.04	7.00 ± 0.03	7.03 ± 0.03	-	7.02 ± 0.04	6.54 ± 0.03
		**5**	6.92 ± 0.13	6.55 ± 0.02	6.77 ± 0.06	6.76 ± 0.05	6.37 ± 0.05	5.84* ±0.02
H		**0**	7.32 ± 0.03	7.18 ± 0.03	7.19 ± 0.06	7.19 ± 0.04	7.23 ± 0.03	7.27 ± 0.02
		**x1**	7.27 ± 0.01	7.16 ± 0.01	7.16 ± 0.03	7.17 ± 0.02	7.19 ± 0.03	7.22 ± 0.02
		**x2**	7.26 ± 0.02	7.15 ± 0.01	7.16 ± 0.02	7.15 ± 0.01	7.15 ± 0.03	7.19 ± 0.02
		**x3**	7.25 ± 0.02	7.15 ± 0.01	7.14 ± 0.02**	-	7.02 ± 0.03	7.18 ± 0.02
		**5**	7.06 ± 0.05	6.92 ± 0.04**	7.13 ± 0.03	7.13 ± 0.04	6.86 ± 0.04	7.17 ± 0.01

Bold values: Different concentrations of metal ions in SC and H conditions, according to Table 2. “*” signifies 10 mM concentration. “**” signifies cases in which some precipitation could be observed upon addition of metal ion salt.

A quick overview of the table below clearly indicates that in 100 mM HEPES buffer, most of the metal ions did not induce significant changes to the pH of the solution even at the highest concentrations of 5 mM, with the subtle change caused only by Fe(III) salt at the highest concentration (pH of 6.89 vs. the initial one of 7.19). In case of SC buffers with lower buffer concentration (and therefore lower buffering capacity), the pH values of the solutions were affected almost in all cases. In particular, for the FLuc SC buffer (with pH before addition of approx. 7, composed of 0.05 M glycine, 0.5 mM EDTA, 5 mM MgSO_4_, and 10 µM ATP; Table 1), the addition of the first aliquot did not change the pH significantly apart from Zn (2.5 mM led to a decrease in pH from 6.89 to 5.39), whereas 5-mM solutions of all the metals led to a comparable drop of 1–1.5 pH units in the case of Ag, Fe(II)-2, and Fe(II)-2 (to 5.97–5.75); almost two pH units drop for Zn (to 5.05); and a dramatic drop to pH 3.53 mM for Cu(II) and 3.29 mM for Fe(III).

Similarly, little to no decrease in the pH was observed with the addition of the first metal aliquot to the SC RLuc buffer (50 mM Tris-HCl, 12.5 mM KH_2_PO_4_, 125 mM NaCl, 0.25 mM EDTA, 0.1% BSA, and 0.3 M sodium ascorbate; Table 1), apart from Zn (drop from 7.03 to 6.54 for 5 mM solution and then 5.84 for 10 mM solution). At 5-mM concentration, the pH of the solution for all of the metals ranged between 6.37 (for Fe(III)) and 6.92 (for Ag).

In case of the NLuc screening buffer (Table 1; PBS 0.75% × 0.1% BSA), no change in the pH was observed even for 5-mM concentration of Ag. Initial addition of Cu(II) (20 µM) did not change the pH, but at 5-mM concentration, the pH dropped to 4.89.5 mM of Fe(II)-1, resulting in the pH of 6.19, whereas Fe(II)-2 solution had a pH of 6.73 at 2.5-mM concentration and 6.28 at 5-mM. Fe(III) induced most dramatic changes with pH 5.74 (2.5 mM) and 2.71 (5 mM). Similar to other buffers, the addition of 5 mM of Zn to NLuc SC led to a pH of 5.55.

#### 3.3.2 Pre-incubation experiments of metal ions with different elements of the buffer

**FIGURE 3:**
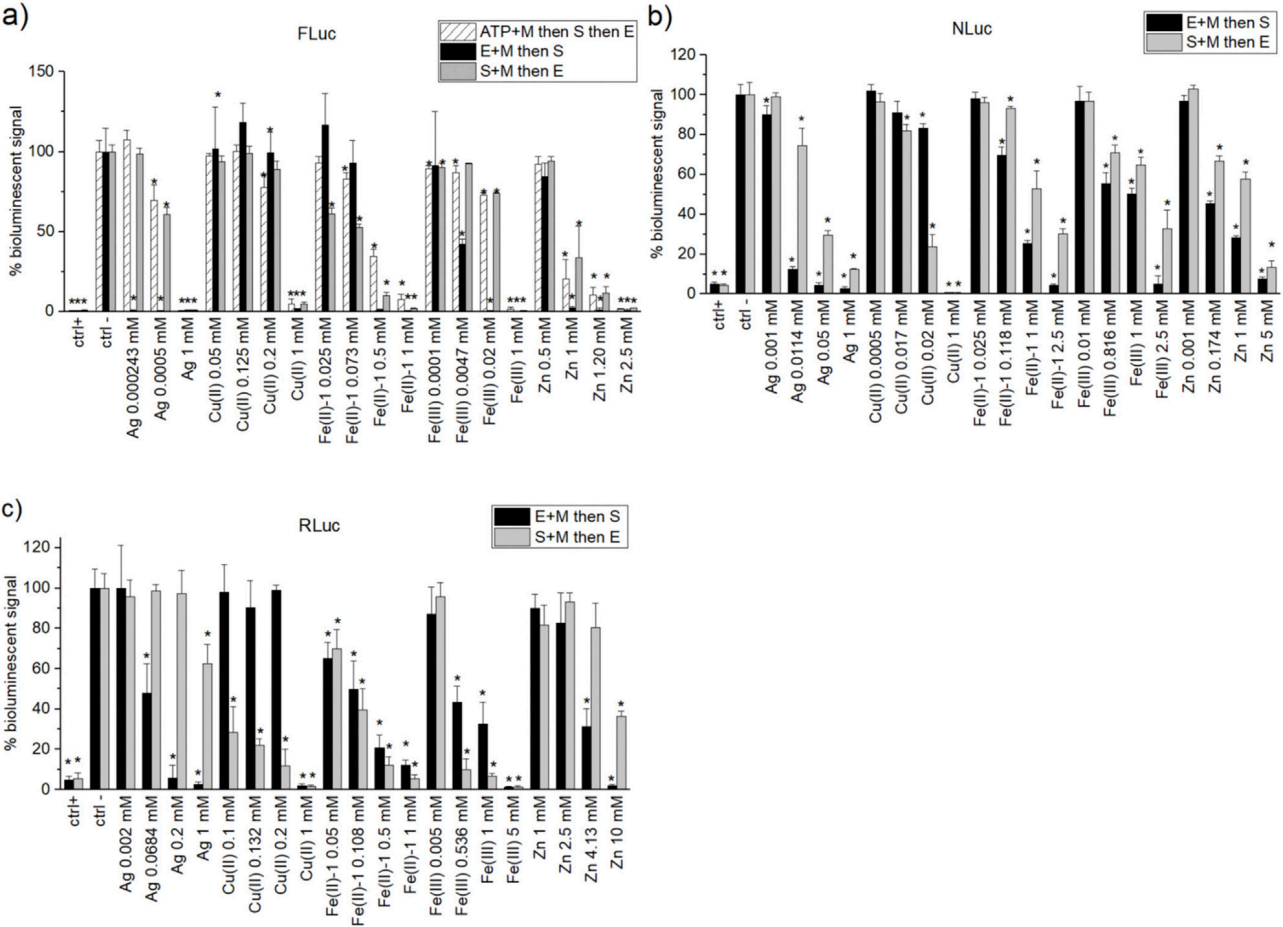
Normalised percentage of bioluminescent signal for **(A)** FLuc, **(B)** NLuc, and **(C)** RLuc assays for different pre-incubations. FLuc assay with subsequent addition of substrate and enzyme to a 30-min pre-incubated solution of ATP + metal salt (white dashed). Addition of a solution of substrate (+ATP, in the case of FLuc) to a 30-min pre-incubated solution of metal salt + enzyme (black). Subsequent addition of enzyme (and ATP in the case of FLuc assay) to a 30-min pre-incubated solution of substrate + metal (grey). Positive controls used are PTC-124 (1 µM), isradipine (10 µM), and BTS (10 µM) for FLuc, NLuc, and RLuc, respectively. The negative controls had the same %DMSO as the corresponding positive controls. Normalisation of the bioluminescent signal was carried out against the negative control output given for each independent pre-incubation condition. Statistical two-tailed Student’s t-test (95% confidence) was carried out between a given sample and the corresponding negative control both at the same pre-incubation conditions. The samples were deemed statistically different from the negative control for *p* < 0.05 and were marked with black “*.”

To further elucidate the origins of the metal ion’s effect on bioluminescence, selected metal ion salts have been pre-incubated either with enzyme for 30 min followed by the addition of the substrate (E + M then S; Figure 3) or with a substrate and subsequent addition of enzyme after 30 min (S + M then E; Figure 3) with enzyme and substrate concentrations kept the same for a given luciferase for all metal salts and concentrations. For the FLuc assay, additionally, metal ion salts were also pre-incubated with ATP as a potentially reactive and yet critical element of the buffer. Selected metal ion salts included Ag, Cu(II), Fe(II)-1, Fe(III), and Zn. Four different concentrations of the salts (for Ag(I) in FLuc, only three concentrations) were selected based on IC_50_ experiments, including 1 mM (same for all the salts), the concentration near the IC_50_ value itself, and one lower and one higher than that (respectively, closer to concentrations inducing the lower and upper limit responses in the IC_50_ curve for a given assay).

In case of the FLuc assay in SC buffer, a pre-incubation of Ag with enzyme leads to almost complete quenching (no signal) for even the lowest Ag concentration, whereas ATP or substrate pre-incubation with Ag salt leads to higher signal than that without pre-incubation (so weakens its quenching effect). A somewhat similar but weaker effect has been observed for Fe(III) at IC_50_ concentration of 4.7 µM and also above (20 µM) and for high Zn concentrations (1 mM and 1.25 mM). Interestingly, in case of Fe(II)-1 salt at lower concentrations (25 μM and 75 µM), pre-incubation with substrate first leads to maintaining of the level of quenching observed when no pre-incubation is applied (same as for IC_50_ value in pure SC buffer), whereas enzyme or ATP-pre-incubation causes higher signal (weaker quenching) than that without pre-incubation. The effect is reversed for 0.5-mM concentration, at which near-complete quenching is observed for enzyme pre-incubation, with least quenching (most residual signal) at ATP pre-incubation.

For both NLuc and RLuc exposed to Ag salt or Zn salt, pre-incubation of metal ion salt with enzyme leads to lower bioluminescent signal (stronger signal quenching) than pre-incubation with the substrate. The same is true for NLuc with Fe(II)-1 and Fe(III), whereas in the case of RLuc, the order of pre-incubation seems to have little to no effect on Fe(II)-1-induced quenching, with less residual signal (stronger quenching) for Fe(III) at higher concentrations. Similarly, but for both NLuc and RLuc, Cu(II) presence quenches the signal more strongly (lower residual signal) in case of pre-incubation with a substrate, with much lower (or even none) quenching observed when pre-incubated with an enzyme first.

#### 3.3.3 Effects of EDTA on metal ions’ interference

**FIGURE 4:**
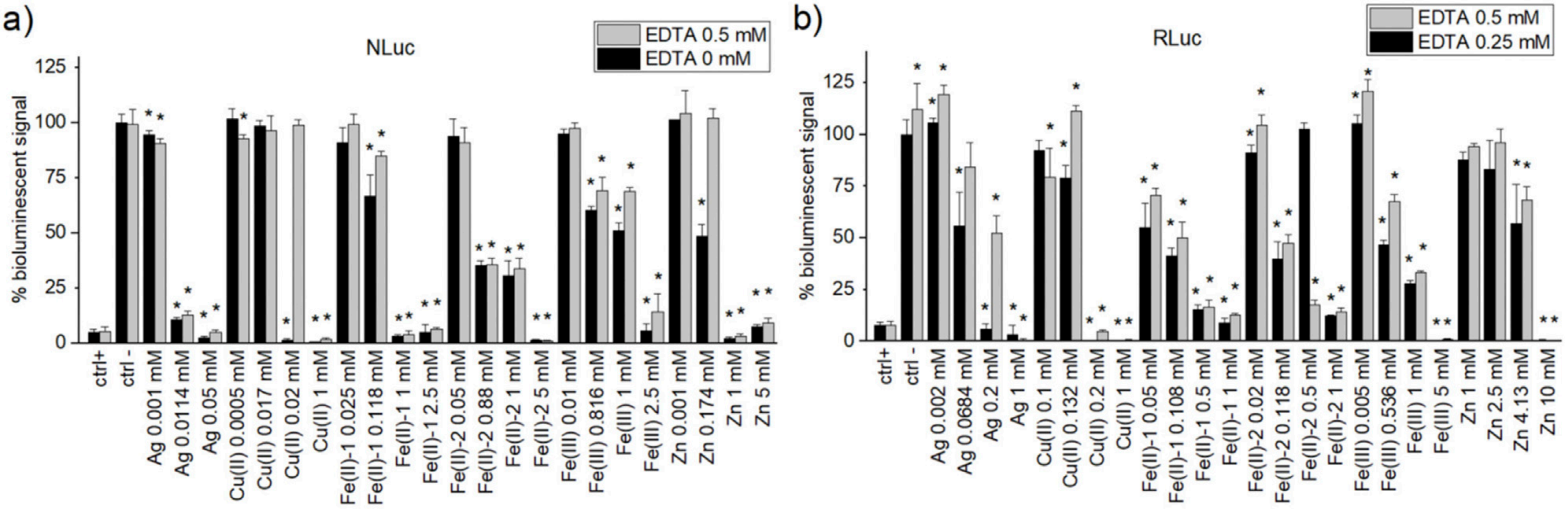
Normalised percentage of bioluminescent signal for **(A)** NLuc and **(B)** RLuc assays with the original EDTA concentration in screening conditions (black; 0 mM for NLuc, and 0.25 mM for RLuc) and the same EDTA concentration as in FLuc assay screening conditions (grey; 0.5 mM). Positive controls used are PTC-124 (1 µM), isradipine (10 µM), and BTS (10 µM) for FLuc, NLuc, and RLuc, respectively. The negative controls had the same %DMSO as the corresponding positive controls. Normalisation of the bioluminescent signal was carried out against the negative control output given by the original screening conditions (lowest EDTA concentration) of the corresponding bioluminescent assay plate. Statistical two-tailed Student’s t-test (95% confidence) was carried out between a given sample (at a given EDTA concentration) and the negative control at lowest EDTA concentration. The samples were deemed statistically different from blank for *p* < 0.05 and were marked with black “*.”

The effect on bioluminescent output of luciferase assays caused by metal ion salts was studied in the presence of EDTA, a recommended additive in FLuc screening buffer, at the same final concentration as in the FLuc assay (0.5 mM), the highest among the three luciferases assays covered in this paper. Original screening buffer conditions for RLuc include half that concentration (0.25 mM), whereas NLuc does not include EDTA as an additive. Selected metal ion salts included Ag, Cu(II), Fe(II)-1,2, and Zn. Four different concentrations of metal ions were selected based on IC_50_ experiments, including 1 mM (same for all metal ions), the IC_50_ value itself, the concentration inducing a lower limit response in the IC_50_ curve, and the one inducing the upper limit response for the given assay. The average percentage of the residual bioluminescence normalised to the negative control (no metal ions, no EDTA) for each of experimental conditions is provided in Supplementary Table S2 and represented on Figure 4.

Relative bioluminescent output from NLuc assay with no additional metal ion salts (negative control, Figure 4A) is not affected by the presence of EDTA (grey), neither is the signal to background ratio (19.7 for no EDTA vs. 19.1 for 0.5 mM EDTA), with Z′ factor decreasing slightly from the initial 0.83 in the absence of EDTA to 0.71 in the presence of EDTA (Supplementary Table S4). However, high EDTA concentration (0.5 mM) in the NLuc assay removes quenching by Cu(II) (at 0.2 mM) and Zn (at 0.174 mM), with a similar but less pronounced effect observed for moderate concentrations of Fe(II)-1 (at 0.118 mM) and even Fe(III) (0.816 mM and 1 mM). In case of RLuc, EDTA at 0.25 mM is an integral component of the SC buffer. An increase from 0.25 mM to 0.5 mM concentration leads to small but statistically significant increase in initial bioluminescence without an absence of additional metal ion salts (black vs. grey bar in negative control on Figure 4B), with insignificant change in the S/B ratio (2.8 at 0.25 mM EDTA concentration and 3.1 at 0.5 mM) but some drop in the Z′ value (0.72–0.60), although still remaining within the acceptable range of above 0.5 (Supplementary Table S4). In general, 0.5-mM EDTA slightly increases the bioluminescent signal for all samples, with Ag at 68 μM and 200 µM concentrations and Cu(II) at 132 µM concentrations (all near the IC_50_ values of the original SC assay) being the most affected (the largest increase in bioluminescent signal in the presence of EDTA).

#### 3.3.4 Effects of GSH on metal ions’ interference

**FIGURE 5:**
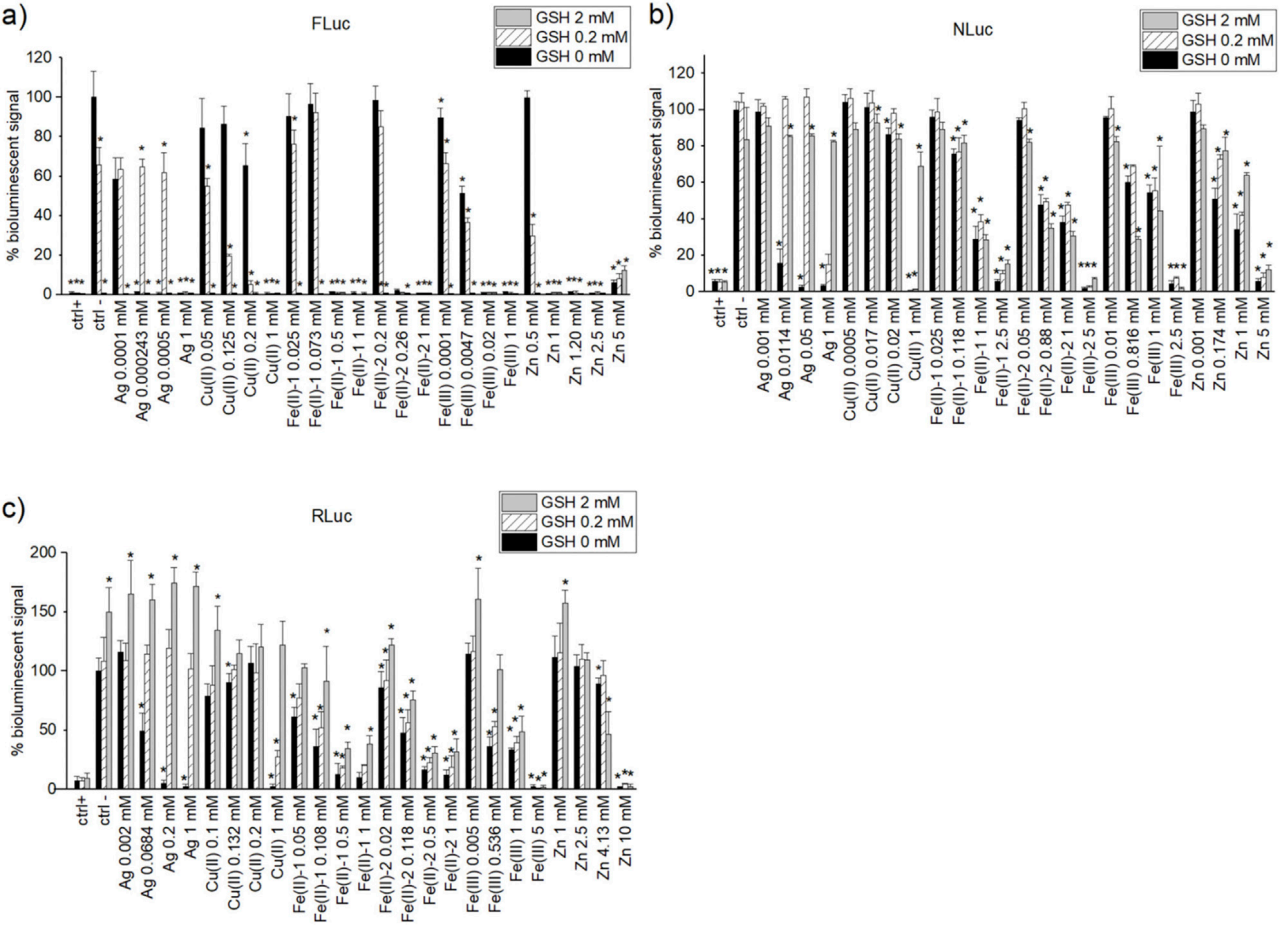
Normalised percentage of bioluminescent signal for **(A)** FLuc, **(B)** NLuc, and **(C)** RLuc assays with 0 mM (black columns), 0.2 mM (white dashed bars), and 2 mM (black columns) concentration of glutathione (GSH). Positive controls used are PTC-124 (1 µM), isradipine (10 µM), and BTS (10 µM) for FLuc, NLuc, and RLuc, respectively. The negative controls had the same %DMSO as the corresponding positive controls. Normalisation of the bioluminescent signal was carried out against the negative control output given by the original screening conditions (lowest GSH concentration) of the corresponding bioluminescent assay plate. Statistical two-tailed Student’s t-test (95% confidence) was carried out between a given sample (at a given GSH concentration) and the negative control at lowest GSH concentration. The samples were deemed statistically different from blank for *p* < 0.05 and were marked with black “*.”

Additional studies on interference in bioluminescent output for selected metal ions (Ag, Cu(II), Fe(II)-1,2, Fe(III), and Zn) were performed in the screening buffer in the absence and in the presence of GSH (0.2 and 2 mM as final concentrations). Four different concentrations of metal ion salts (except Ag with three concentrations) were selected based on IC_50_ experiments, including 1 mM (same for all metal ions), the concentration near IC_50_ value of the metal ion salt, the concentration inducing a lower limit response in IC_50_ curve, and the one inducing the upper limit response for the given assay. The average percentage of the residual bioluminescence normalised to the negative control (no metal ions and no GSH) for each of experimental conditions is provided in Supplementary Table S3 and represented in Figure 5.

Generally, in the FLuc assay, negative control conditions (no metal ions, only GSH) demonstrate a GSH concentration-dependent quenching of bioluminescent signal with increasing GSH concentration. The same trend is also largely observed for most metal ion salts. In addition, at 2-mM concentration of GSH, no signal has been observed in any sample, independent of the co-presence of the metal ions (Figure 5A). For NLuc, the bioluminescent signal of the negative control (no metal ions, no inhibitors) remains unaltered even at 2-mM GSH concentration (Figure 5B). However, bioluminescent signal increases with increasing concentration of GSH for Ag, Cu(II), and Zn salts. For almost all concentrations of iron salts, GSH makes little to no difference, with some trending toward higher signal for increasing GSH concentration at 5 mM of Fe(II)-1 and Fe(II)-2, and a statistically significant drop in bioluminescent signal when moving from 0.2 mM to 2 mM of GSH at the original SC buffer IC_50_ concentration of Fe(III) (0.816 mM). The negative control for RLuc (Figure 5C), on the other hand, presents a statistically significant increase in activity with 0.2-mM GSH and even more with 2-mM GSH. Interestingly, GSH, especially at 2-mM concentration, leads to the weakening of inhibition of RLuc by all of the tested metals, with the effect being most pronounced for Ag and Cu(II), and least for Zn.

## 4 Discussion

Screening and subsequent investigations have been performed for a broad panel of environmentally and biologically relevant metal ions and their concentration ranges (Sigel and Sigel, 2000; Maret, 2016; Shamsollahi and Partovinia, 2019; Adams et al., 2020), increasing the significance of any observed metal ion-induced quenching of bioluminescent signals. These effects could arise from various processes, including direct interaction with a luciferase enzyme, interference with substrate binding or turnover, substrate and reactants’ sequestration, or alterations in the chemical environment required for the bioluminescent reaction. As all of those processes are broadly dependent on the same type of affinities of metal ions to various ligands such as amino acid residues on enzymes, chelating motifs on substrates, reactants, or elements of the buffer, discerning these mechanisms remains challenging. Diverse experimental designs discussed above allowed quantifying the effects and providing insights into the role of reactants and buffer components in metal-mediated interference with luciferase-driven bioluminescent signal generation.

### 4.1 Nature of the metal ion and relative trends in the extent of metal ions’ interference

Nearly all tested metal ions’ salts led to statistically significant reduction of bioluminescent signal of the FLuc system even at 1-mM concentration. Alkali metal ions salts showed the weakest inhibition that is relatively independent of the FLuc assay buffers (similar for both SC and H), which might suggest a non-specific quenching mechanism (e.g., ionic strength modulation or similar). Nevertheless, their ubiquitous presence in buffers and biological as well as environmental samples, often at well into millimolar concentrations, can have a significant effect on bioluminescent readout and requires careful consideration.

Subsequent order of strength of inhibition followed the series: Mn(II) < Mg < Ca and was concentration-dependent and relatively similar in SC and H buffer conditions. When combined with little to no inhibitory effect of these salts observed for ATP-independent NLuc and RLuc activity, this result supports the previously reported mechanism of quenching based on ATP binding (Wang et al. 2011). Although many different divalent metal ions can bind to ATP, the optimal choice of metal catalyst, commonly used in FLuc assay buffers, includes generally moderate concentrations of smaller Mn^2+^ and Mg^2+^ ions. They bind to two phosphate residues of ATP, facilitating nucleophilic attack of luciferin carboxylate on ATP and the departure of pyrophosphate–metal ion complex as a good leaving group, leading to the formation of luciferin–AMP intermediate in the FLuc active site (Scheme 1). However, as ATP binding strength decreases in the order Mn^2+^ > Mg^2+^ > Ca^2+^, higher concentrations of Mn^2+^ and Mg^2+^ can have adverse effect on the reaction, whereas the larger size of Ca^2+^, in turn, prohibits ATP from binding to luciferase in the first place, leading to a decrease in the bioluminescent signal supporting our observations (Wang et al. 2011).

All other metal ion salts tested in this manuscript exhibit virtually complete inhibition of FLuc-mediated bioluminescent signal emission even at 1-mM concentration. These qualitative effects are confirmed on the quantitative level in the FLuc HEPES buffer assay with the order of inhibition strength, Cu(I), Cu(II), Ag > Zn, Cd > Fe(II), Pb(II), > Fe(III), Ga(III), Pt(II) > Ni(II)/Co(II) > Ca > Mg > Mn(II) (Figure 2, Supplementary Figure S1; Tables 3, 4), reflecting previously reported similarity to the Irving–Williams series, which predicts relative stabilities of metal ion–ligand almost independently of the nature of the chelator (Lee et al., 1970; Wen et al., 2001; Wang et al., 2011). Similar order of affinities and stabilities or resulting complexes have been proposed to explain metal ion–protein binding in biology (Maret 2016), supporting direct metal ion–enzyme interaction as a cause of inhibition observed in our experiments.

Previous works on metal ion effects on FLuc bioluminescence indicated two possible sites of metal ion binding where both play a role in favouring a formation of an excited state oxyluciferin product responsible for green bioluminescence: 1) native cysteine residues responsible for allosteric stabilisation (for Zn^2+^, Cd^2+^, and other heavy metal ions) and 2) pH-sensitive histidine/glutamate couple H310/E354 and/or glutamate–arginine pair (E311/R337) near the active site that participates in oxyluciferin deprotonation and direct conformational stabilisation (for Fe^2+^ and other first row transition metal ions, possibly also Zn^2+^) (Wang et al., 2011; Viviani et al., 2018).

The pre-incubation experiments for selected metal ion salts (Ag, Cu(II), Fe(II), Fe(III), and Zn) at concentrations near and above IC_50_ values provided insights into these potential mechanisms, revealing that the order of addition and the incubation time with specific components (enzyme, substrate, or cofactors) can modulate the extent of metal ion interference. For instance, the fact that virtually for all five metal ion salts, pre-incubation with FLuc enzyme, but not when pre-incubated with substrate or ATP, led to similar or more pronounced quenching than when all reactants were mixed at the same time, further suggests that direct interaction between metal ion and the enzyme is responsible for signal quenching and that buffer element can sequester these ions, alleviating the signal reduction. This is in line with the fact that whereas Mg, Ca, and Mn(II) are believed to affect FLuc fluorescence via interaction with ATP and altering the efficiency luciferin–AMP formation, other divalent and trivalent metal ions were suggested to interact directly with the residues on the enzyme (Lee et al., 1970; Wang et al. 2011; Viviani et al., 2018), leading to a change in the stability of the light-emitting oxyluciferin product.

Additionally, whereas Fe(II), like other tested metal ions, still exhibits the strongest inhibition when pre-incubated with FLuc, substrate-metal ion pre-incubation leads to stronger quenching than ATP-metal ion pre-incubation. This supports previously reported hypothesis that apart from direct enzyme–metal ion interaction, FeSO_4_ can also reduce bioluminescent signal through the precipitation of D-luciferin substrate.

### 4.2 Variability in response for different luciferase systems

General trend of metal ion-induced inhibition was observed across all three luciferases, with Ag, Cu(I), and Cu(II) being seemingly most potent, followed by Fe(II) and Fe(III), but the quantitative extent of interference varied significantly between them (Figure 2; Table 3). The FLuc assay (especially HEPES one) exhibited the highest sensitivity to metal ion interference, with several metal ions causing substantial quenching even at low concentrations (0.01 mM). This is also confirmed by the IC_50_ values for metal ion salts ranging from sub-micromolar to low micromolar concentrations in HEPES and micromolar to sub-milimolar in SC for 13 out of 14 tested salts. This susceptibility could be attributed to unique structural features as well as multicomponent (requires ATP) and multistep catalytic mechanism of FLuc, as presented in Scheme 1 (Conti et al 1996; Kaskova et al. 2016). In contrast, NLuc and RLuc that do not require ATP and both use similar substrate types that are, however, significantly different from FLuc, displayed relatively higher tolerance to metal ion interference, with fewer metal ion salts exhibiting significant inhibitory effects at low concentrations and most IC_50_ values oscillating in the higher micromolar to millimolar range.

Higher FLuc sensitivity is particularly visible when comparing low metal-affinity HEPES-based assay variants that were designed to minimise buffer–metal binding and the buffer variability between different luciferases (HEPES buffer has been shown to have negligible binding to metal ions; however, each of those assays still differ by the additives that came out essential to ensure sufficient assay quality). In those H-type assays, all active metal ion salts tested exhibited from two (Fe(II) salts in RLuc H vs. FLuc H assay) to over thousand times (Cu(I) for NLuc H vs. FLuc H assay) higher IC_50_ values (lower inhibition) for NLuc H and RLuc H assays than in the FLuc H one. The variability in inhibition efficiency is much smaller between NLuc and RLuc (only up to 10 times the difference), with NLuc being generally more resistant than RLuc. This is in line with the fact that NLuc is a bioengineered version of natural RLuc, optimised for smaller size, bigger efficiency, and better stability and robustness. The exceptionally stronger inhibition of NLuc than RLuc in the H assay by Zn, Cd, and Pt(II) might stem from the differential presence of ascorbate in the RLuc H assay, which is a known and highly potent Zn ionophore (i.e., binds strongly to Zn^2+^ and potentially also to chemically similar Cd^2+^ and Pt^2+^ ions, depleting the effective concentration of “free” metal ions in the RLuc H assay).

With limited insights into the mechanism of NLuc and RLuc activity and regulation (Liu and Escher, 1999; Loening et al. 2006; Schenkmayerova et al., 2023; Nemergut et al. 2023), the mechanism of metal ion interference with RLuc- and NLuc-mediated bioluminescence is very poorly studied. Pre-incubation experiments described in this manuscript for NLuc SC and RLuc SC assay conditions shed some light on this process. In particular, similarly to FLuc, pre-incubation of NLuc and RLuc with Ag and Zn, NLuc with Fe(III), and RLuc with Fe(II) led to more pronounced quenching, indicating direct metal–enzyme interaction. Reversed effects observed for pre-incubation of furimazine (NLuc substrate) with Cu(II) and coelenterazine (RLuc substrate) with Cu(II) and Fe(III) suggest substrate interaction or sequestration by those metals.

### 4.3 Influence of buffer systems and additives on metal ion-induced effects

The nature and composition of the buffer system played a crucial role in modulating the extent of metal ion interference observed in the bioluminescent assays by binding/precipitation and, therefore, lowering of effective availability of metal ions for interaction with luciferase/substrate/reactants.

The screening conditions (SC) included buffers with higher affinity to and/or lower solubility with metal ions (e.g., glycine, phosphates, or chlorides at 10–100 mM) and containing additional components like sodium ascorbate (in RLuc at 0.3 M), TRIS, and EDTA (in FLuc and RLuc, both approximately 0.5 and 0.25 mM, respectively), as well as BSA for NLuc and RLuc (Table 1). In contrast, HEPES buffer (100 mM), which is known to exhibit negligible metal ions affinity (Brooke et al. 2015), was used to unify the buffering system across all luciferases; however, some additives (like BSA for NLuc and RLuc, ascorbate for RLuc, and MgSO_4_ for FLuc) could not be eliminated as they turned out to be essential to ensure sufficient luciferase activity and quality, and robustness of the assays (Z′ factor and signal-to-background ratios above recommended thresholds).

Certain metal ions, especially Cu(II) and Fe(III) at millimolar concentration, led to substantial pH decrease in SC buffers and especially in FLuc assay, potentially disrupting the optimal pH range for FLuc activity (Schenkmayerova et al., 2023) and demonstrating superior buffering capacity of HEPES buffering systems. This, together with simpler buffer composition and higher sensitivity of all enzymes to metal ion effects in the HEPES buffer, suggests that other mechanisms, such as direct enzyme inhibition or substrate interference, may be more prominent drivers of metal ion-induced quenching in this system.

Further insights into buffer effects could be obtained by analysis changes of IC_50_ values when moving from higher buffering capacity but lower metal affinity HEPES systems to more complex, multicomponent, and ligand-rich SC buffers. In case of NLuc assays, both magnitude and order of inhibition by metal ion salts remained largely the same in both H and SC, with the exceptions of the loss of Cd activity in SC vs. HEPES and an opposite stronger inhibition of NLuc by Ag in HEPES, which might be partially explained by a rare but yet previously observed affinity of HEPES toward Ag + ions at even micromolar concentrations (Brooke et al. 2015). Nevertheless, by far, the most dramatic loss signal inhibition of even three to five orders of magnitude (from sub-micromolar or single micromolar IC_50_ values to >100 µM) was found when moving from FLuc H to the SC buffering system with Cu(I) (over 11,000 times) > Cu(II) and Zn (over 1,000 times) > Cd and Pb (over 100 times). This can be linked to a high glycine content in FLuc SC (50 mM) that according to previously reported data exhibits the highest affinity for zinc and copper, followed by iron cadmium and lead, that is, the same set of metal ions that experience highest activity loss (Izatt et al. 1972; Kiss et al. 1991; Marino et al., 2006; Remko and Rode, 2006; Sajadi 2011). Interestingly, similarity of IC_50_ values for Cu(I), Cu(II), Fe(II), and Zn between RLuc SC and FLuc SC assays (despite large biochemical and mechanistic differences between these luciferases) and 10 times lower (stronger) values for those metals in NLuc SC correlate with the presence of similar quantities of TRIS, EDTA, and carboxylate ligands (glycine and ascorbate; Table 1) in RLuc SC and FLuc SC but not in NLuc SC.

EDTA as a promiscuous and yet relatively effective chelator of divalent and trivalent metal ions (due to its polydenticity, flexibility, and multiple pKa’s) is a common and previously recommended (Hermann et al., 2013) protective agent that can sequester metal ions form solution. This effect has also been observed in our work, where an addition of EDTA alleviated quenching of RLuc and NLuc bioluminescence by Cu(II) and Zn(II), and also to some extent by Fe(II) Fe(III) and Ag. The effect is dependent on relative concentration of EDTA and metal ions but is in line with the relative stabilities of EDTA–metal ion complexes (Cu > Zn > Fe > Mn > Ca > Mg; Xiao and Wedd, 2010; Goli et al 2012). This, together with the fact that Zn and Cu(II) IC_50_ values for FLuc and RLuc (bot not NLuc) drastically increase when moving from simpler H to TRIS and EDTA-containing SC conditions, points at those chelators as key protective additives of analysed luciferase buffer systems.

The presence of glutathione (GSH), an ubiquitous cellular antioxidant, exhibited differential effects on metal ion-induced quenching across the three luciferase systems. In the FLuc assay, GSH led to quenching of initial bioluminescence in a concentration-dependent manner (partial at 0.2 mM and almost complete at 2 mM). Quenching effect, especially at higher 2 mM concentration, was observed independently on the presence or the concentration of metal ions, indicating a likely direct interference of GSH with FLuc activity. Possible pathways for this interference might include interactions with enzyme cysteine residues responsible for stabilisation of the bioluminescence-promoting enzyme structure and/or interference in FLuc-catalysed luciferin oxidation step (European Chemical, 2024).

Conversely, the presence of GSH did not significantly affect basal bioluminescence of NLuc and even increased it for RLuc (control at 2 mM). Most prominent protective effect of GSH against Ag-induced and Cu(II)-induced bioluminescent signal reduction for all three luciferases is in line with the metal sequestration mechanism of action, as expected from the propensity of GSH to react and/or bind soft Lewis acids according to the HSAB theory. Nevertheless, 1) differential effects of GSH on basal bioluminescence of those luciferases and 2) the presence of GSH-sensitive and oxidation-sensitive cysteine residues (Liu and Escher, 1999; Loening et al 2006) implying the enzyme-centred mechanism suggest that both GSH–metal and GSH–enzyme interactions are in play and need to be considered.

These findings collectively highlight the importance of considering buffer composition and potential interactions between buffer components and metal ions when evaluating their impact on bioluminescent assays.

## 5 Conclusions

In this study, we screened comprehensive and diverse panel of metal ion interferents, highlighting their significant influence on bioluminescence-based high-throughput screening (HTS) assays involving firefly luciferase (FLuc), Renilla luciferase (RLuc), and NanoLuc luciferase (NLuc). It is noteworthy that the observed quenching effects occurred within biologically and environmentally relevant concentration ranges of metal ions, underscoring a significant impact on HTS campaigns and the subsequent interpretation of screening data.

The susceptibility to metal ion-induced quenching varied among the three luciferase systems, with FLuc exhibiting the highest sensitivity, followed by RLuc, and NLuc being the most stable. This variability and differential effects of buffer compositions, pre-incubation, EDTA, and GSH highlight the complexity of the underlying mechanisms. The findings have important implications for the design, optimisation, and interpretation of bioluminescence-based HTS assays, emphasising the need for rigorous assay validation protocols and potentially incorporating mitigating strategies, such as the use of chelators or alternative luciferase systems, depending on the anticipated metal ion composition of the samples or compound libraries.

Furthermore, in this study, we reported a panel of three pairs of high-quality HTS assays for the activity of each of the three luciferases, with different buffering capacities, buffer compositions, and often complementary sensitivities to different metal ions. This allows future users to select an assay that is best suited to the type of anticipated metal contamination. Additionally, the FLuc HEPES assay serves as a wide-spectrum metal ion detection assay with sub-micromolar sensitivity for quality control of samples/compound libraries and for hit validation to flag possible metal-induced false-positives.

By elucidating the influence of metal ions on these widely used bioluminescent reporter systems and providing a set of new high-quality HTS assays for the activity of the three most commonly used luciferases, in this study, we contribute to the ongoing efforts to enhance the robustness, reliability, and interpretability of HTS campaigns in drug discovery and biological research.

## Data Availability

The original contributions presented in the study are included in the article/Supplementary Material; further enquiries can be directed to the corresponding author.
