# Supplementary material for: Interference of metal ions on the bioluminescent signal of firefly, Renilla, and NanoLuc luciferases in high-throughput screening assays
Source: Front Chem. 2024 Nov 26;12:1436389. doi: 10.3389/fchem.2024.1436389 (PMC11628255; doi:10.3389/fchem.2024.1436389)
Supplement: Supplementary file 3 [file DataSheet1.docx]

***Supplementary Material***

**Interference of metal ions on bioluminescent signal of Firefly, Renilla and Nano-Luciferases in High-Throughput Screening assays.**

**Francesca Canyelles i Font^1^, Krzysztof Żukowski^1^, Masroor A. Khan^1^, Dorota Kwiatek^1^, Jacek L. Kolanowski^1^***

*** Correspondence:**dr hab. Jacek Łukasz Kolanowski
[jkolanowski@ibch.poznan.pl](mailto:jkolanowski@ibch.poznan.pl) (current address: j.kolanowski@victorchang.edu.au)

# Supplementary Data

Supplementary Material should be uploaded separately on submission. Please include any supplementary data, figures and/or tables.

Supplementary material is not typeset so please ensure that all information is clearly presented, the appropriate caption is included in the file and not in the manuscript, and that the style conforms to the rest of the article.

# Supplementary Figures and Tables

For more information on Supplementary Material and for details on the different file types accepted, please see [here](https://www.frontiersin.org/guidelines/author-guidelines#supplementary-material).

## Supplementary Figures


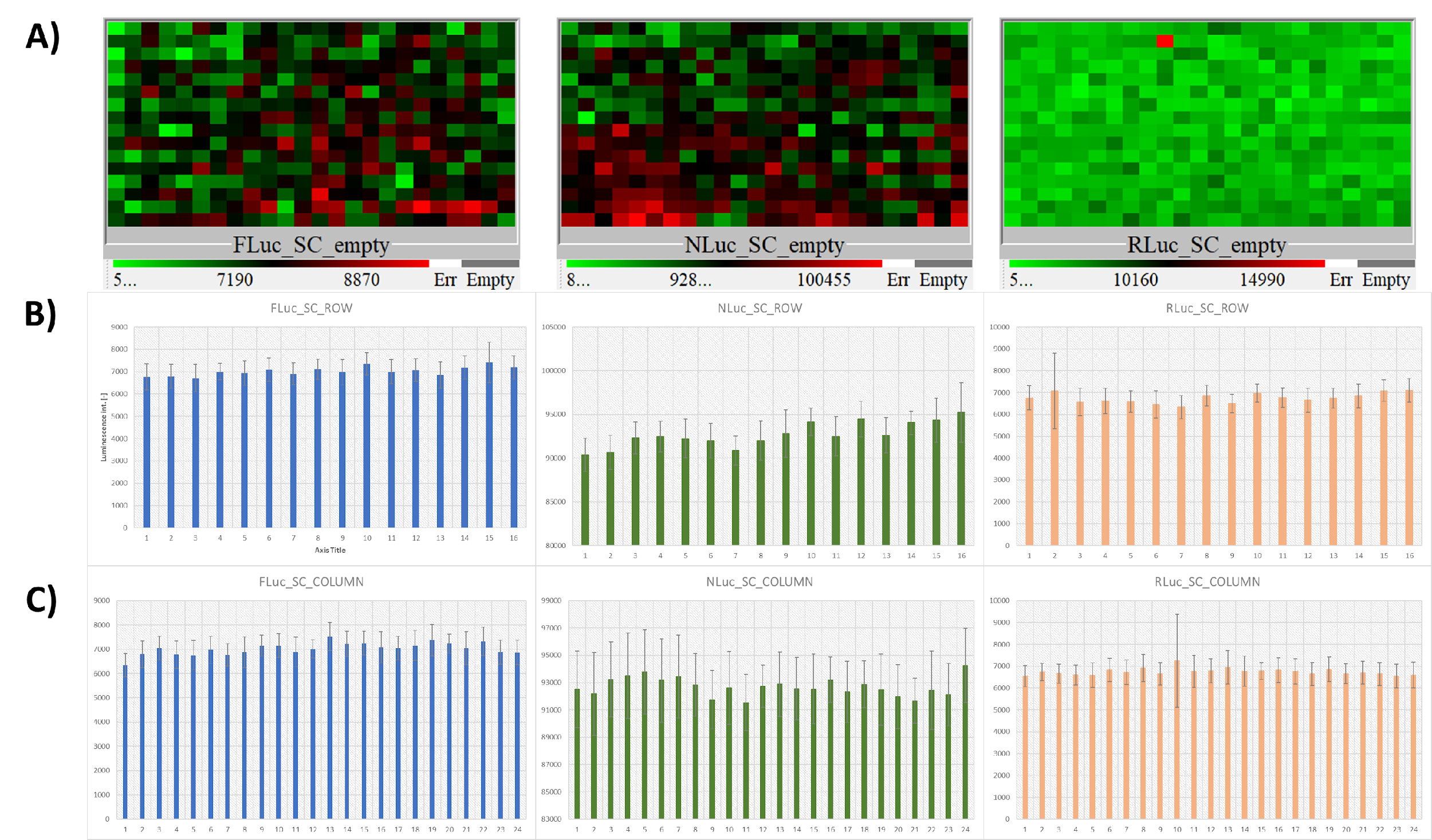


**Supplementary Figure S1**. Results of testing the plate effect for all luciferases under screening conditions for the validation plate. Heatmaps (A), average bioluminescence signals with standard deviations for rows (B) and columns (C).


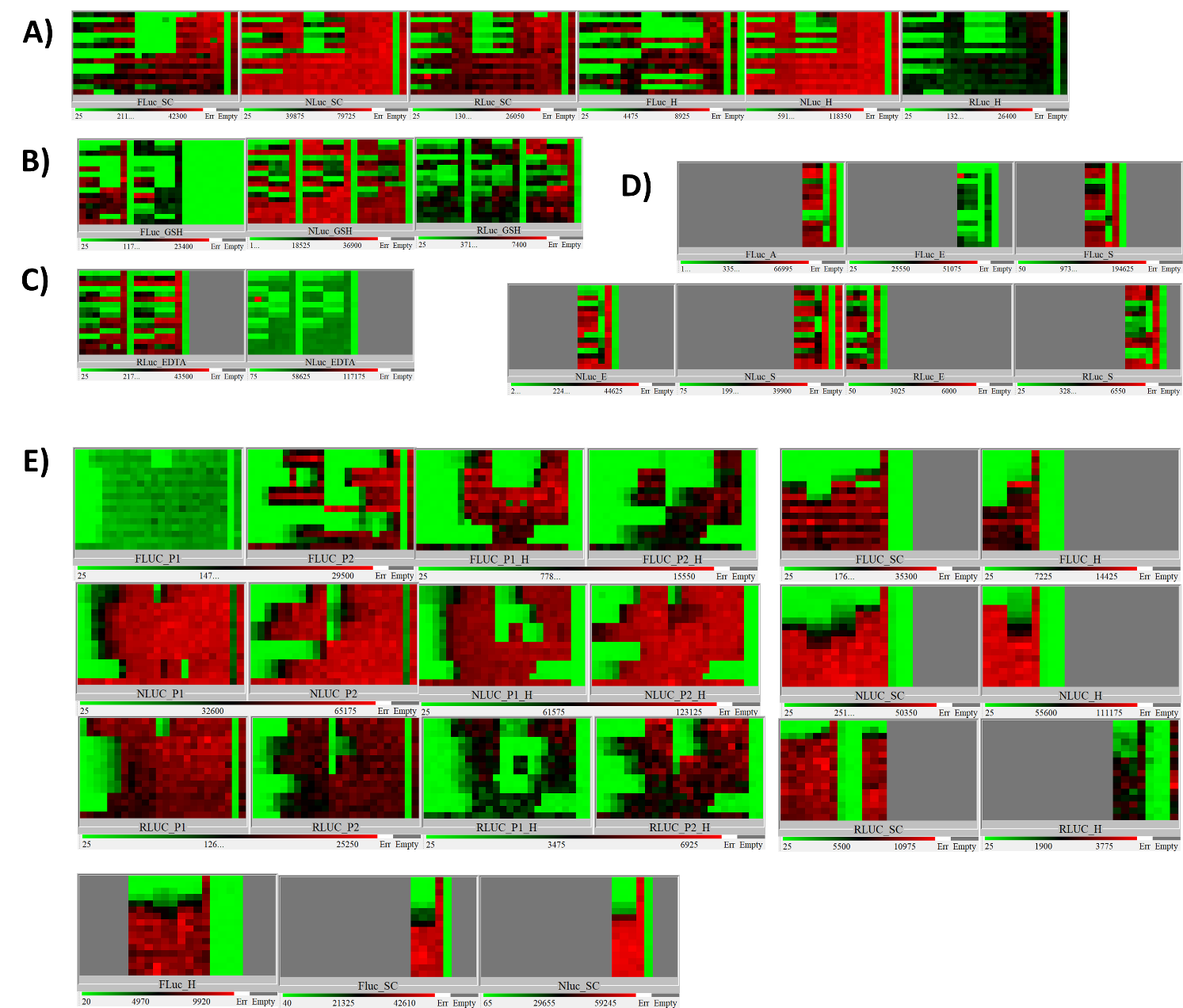


**Supplementary Figure S2**. Platemaps for all experiments reported. Positive controls used are PTC-124 (1 µM), isradipidine (10 µM), and BTS (10 µM) for FLuc, NLuc, and RLuc, respectively. The negative controls had the same %DMSO as the corresponding positive controls. A: Bioluminescent output from optimised luciferase activity assays for FLuc, NLuc, and RLuc in SC and in H buffers in the presence of most active metal ion salts in three different concentrations: 0.01 mM, 1 mM, and 5 mM. B: Bioluminescent output for FLuc, NLuc and RLuc assays with 0 mM, 0.2 mM, and 2 mM GSH concentration in screening buffer conditions. C: Bioluminescent output for NLuc and RLuc assays with the original EDTA concentration in screening buffer conditions (0 mM for NLuc, and 0.25 mM for RLuc) and the same EDTA concentration as in FLuc assay screening conditions (0.5 mM). D: Bioluminescent output for FLuc, NLuc and RLuc assays for different pre-incubations in screening buffer conditions. E: Bioluminescent output from samples for determination of IC50 values for each luciferase activity assay in SC and in H buffers. Corresponding plate designs can be found in File_1 from SI.

*
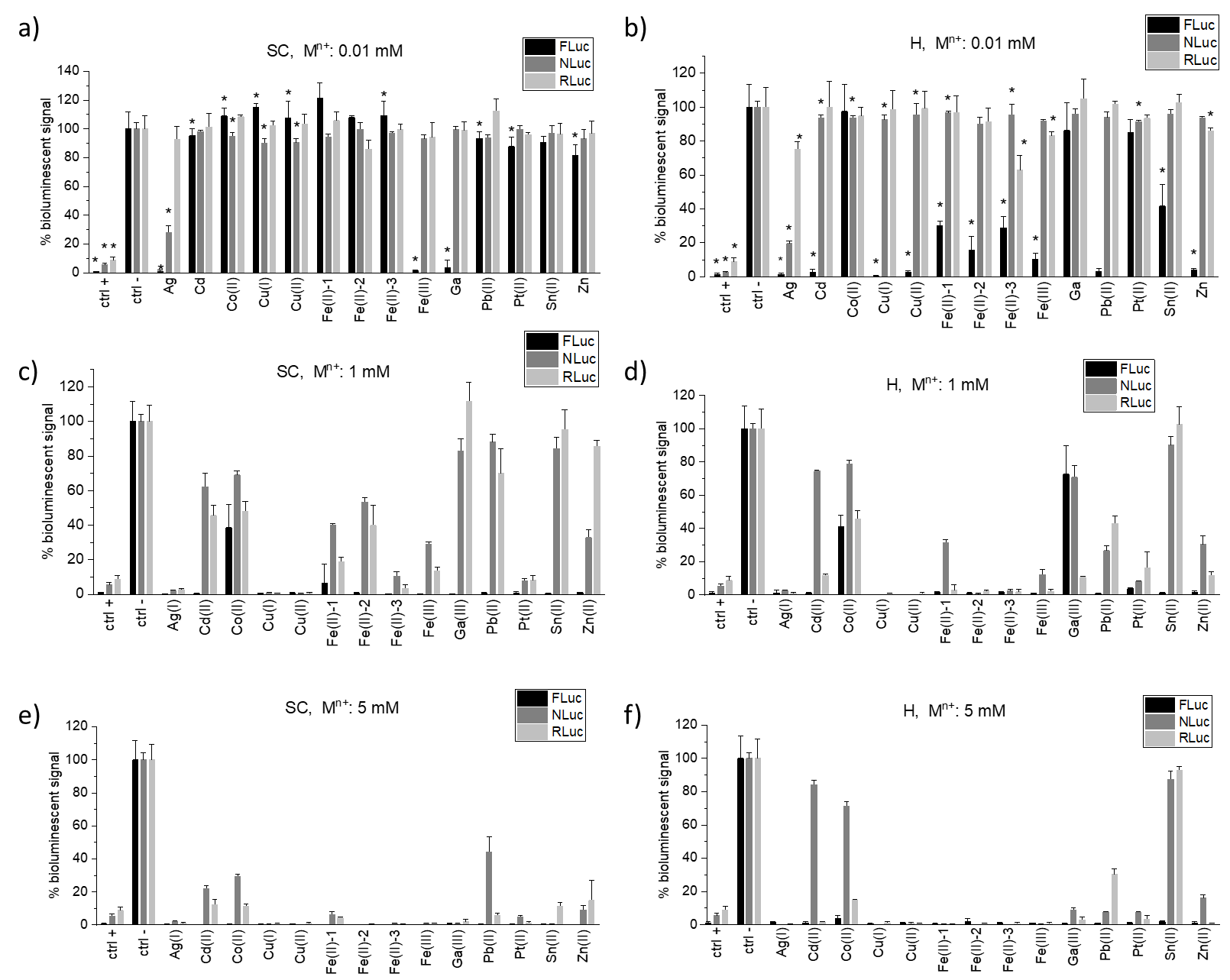
*

**Supplementary Figure S3**. Normalised percentage of bioluminescent signal from optimised luciferase activity assays for FLuc (black), NLuc (dark grey), and RLuc (light grey) in SC (a,c,e) and in H (b,d,f) buffers in the presence of most active metal ion salts in three different concentrations: 0.01 mM (a,b), 1 mM(c,d), and 5 mM (e,f). Positive controls used are PTC-124 (1 µM), isradipidine (10 µM), and BTS (10 µM) for FLuc, NLuc, and RLuc, respectively. The negative controls had the same %DMSO as the corresponding positive controls. Normalisation of the bioluminescent signal was carried out against the negative control output of the corresponding bioluminescent assay plate. Statistical two-tailed Student t-test (95% confidence) was carried out between a given sample and the corresponding negative control. The samples were deemed statistically different from blank for p < 0.05 and were marked with black “*” only for 0.01 mM final concentration of metal ion. All metal ion salts at a final concentration of 1 mM (except for RLuc SC – Ga, Sn(II), and FLuc H - Ga) and all of them at 5 mM are statistically significant.

| Metal | FLuc | NLuc | RLuc |
| --- | --- | --- | --- |
| Ag | 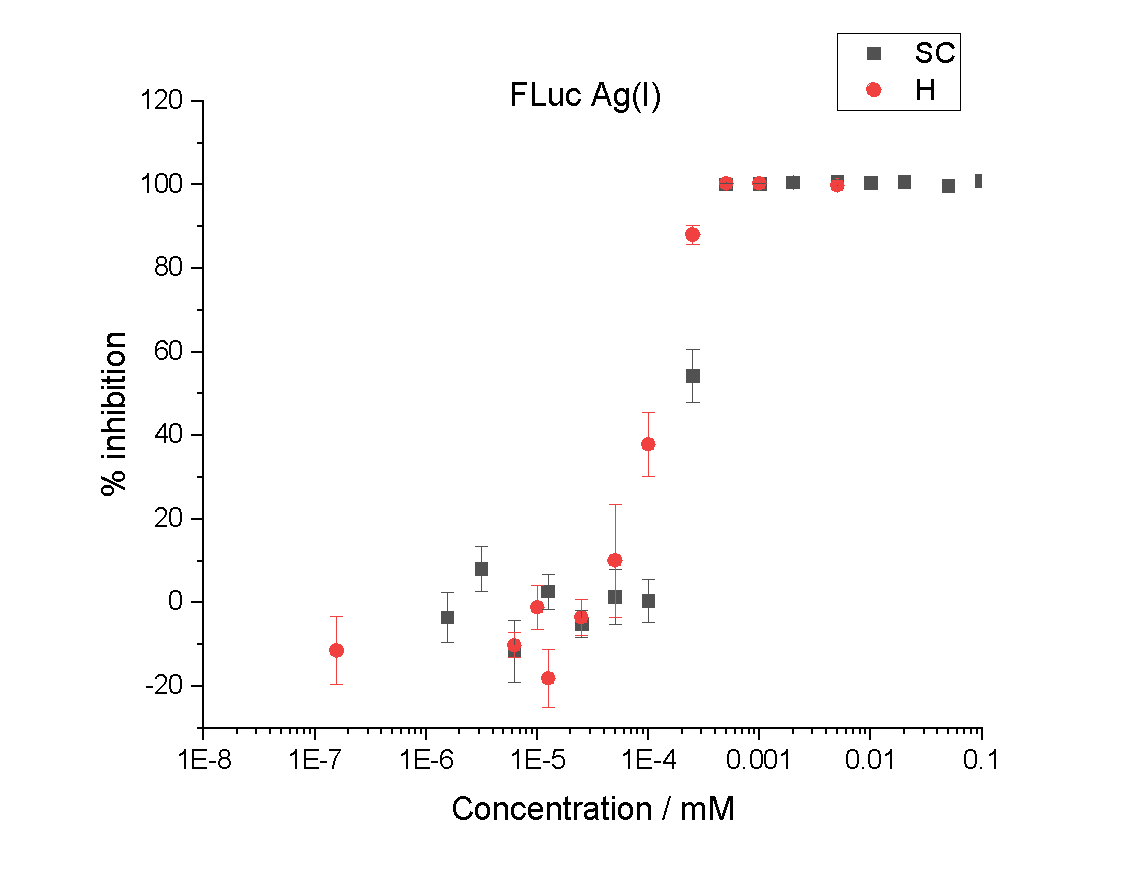 | 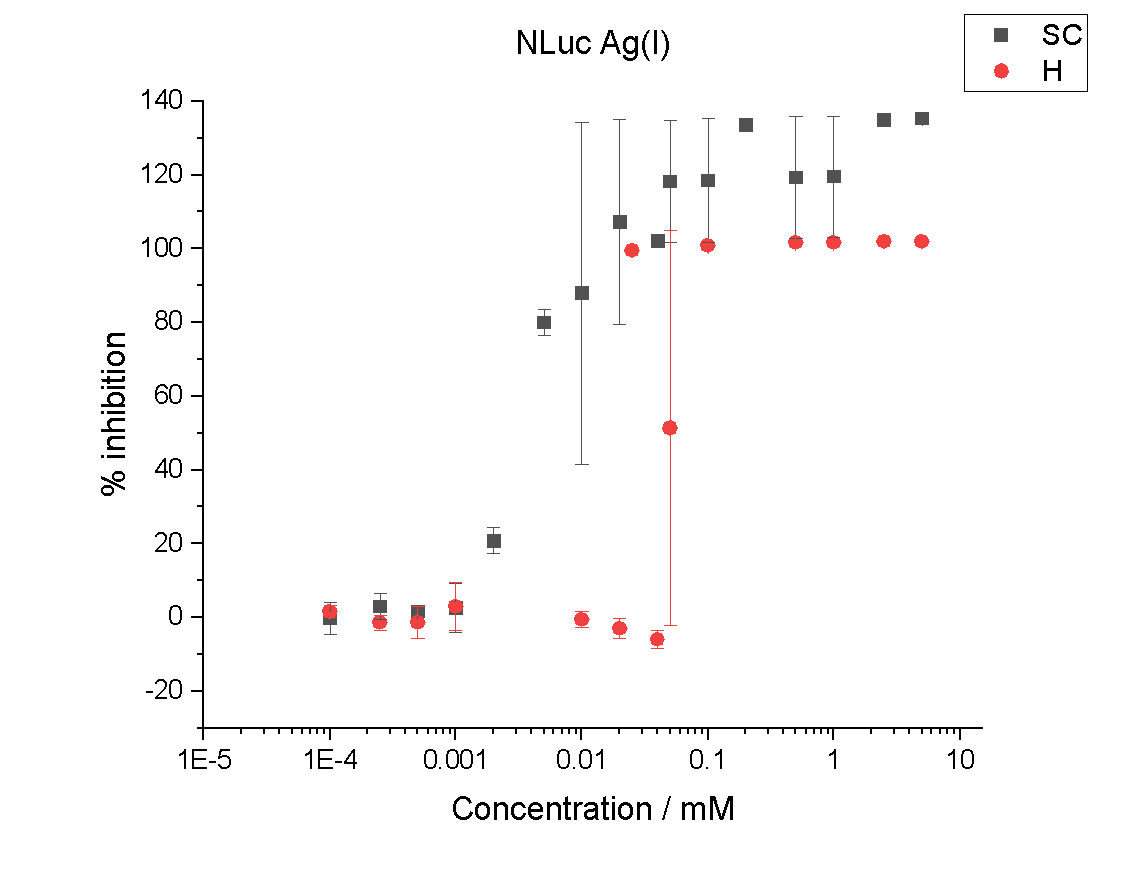 | 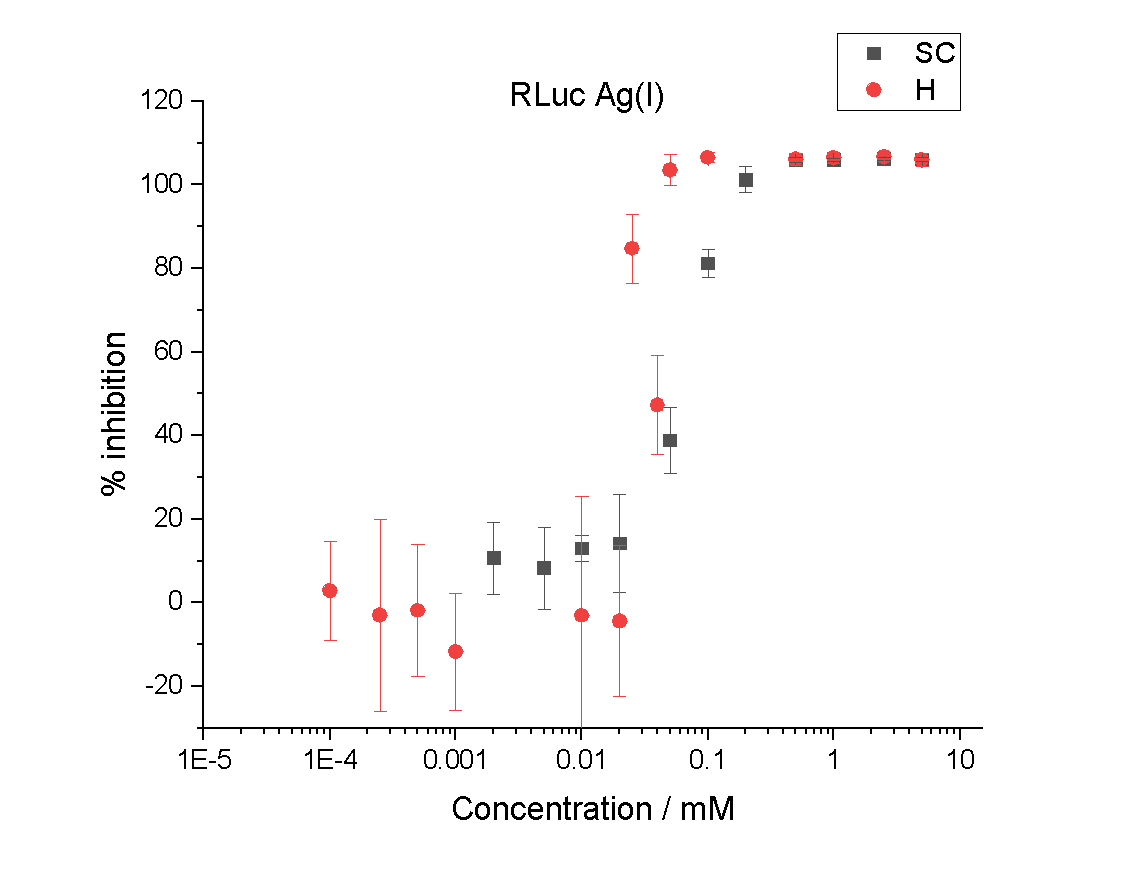 |
| Cd | 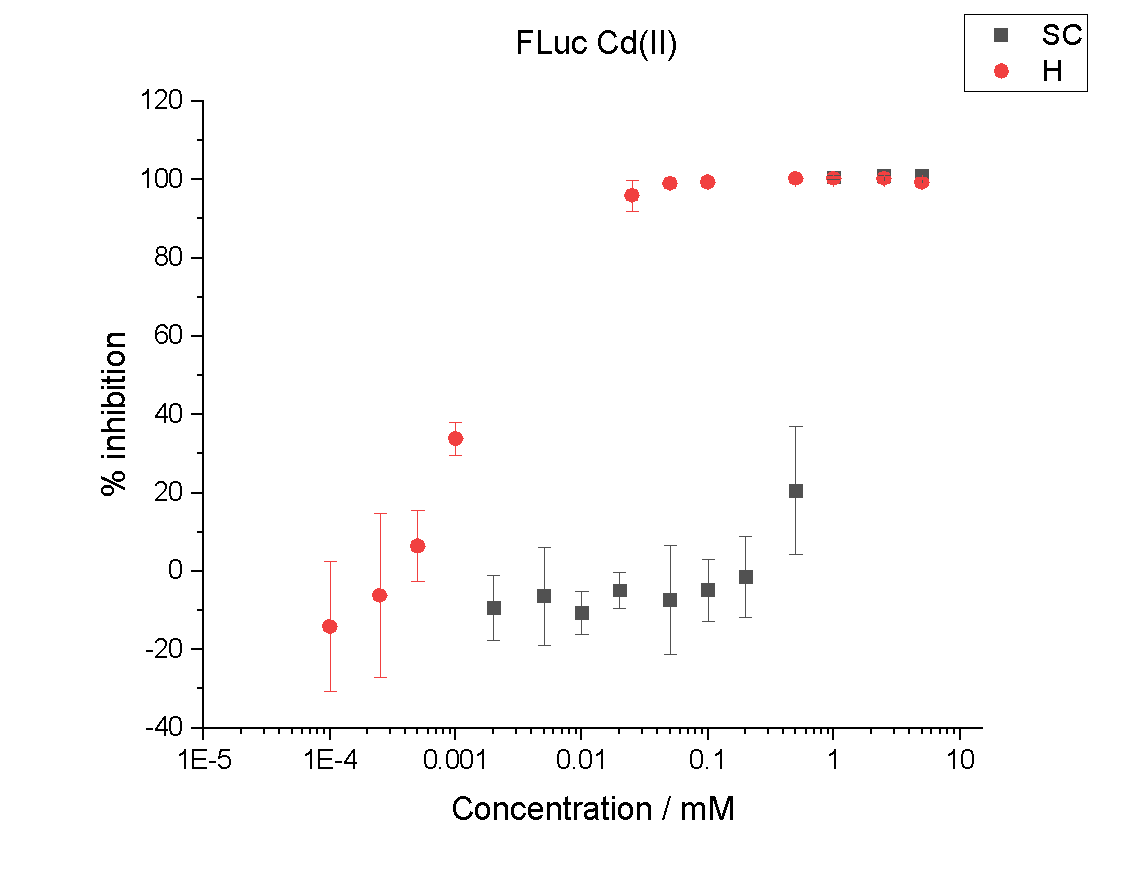 | 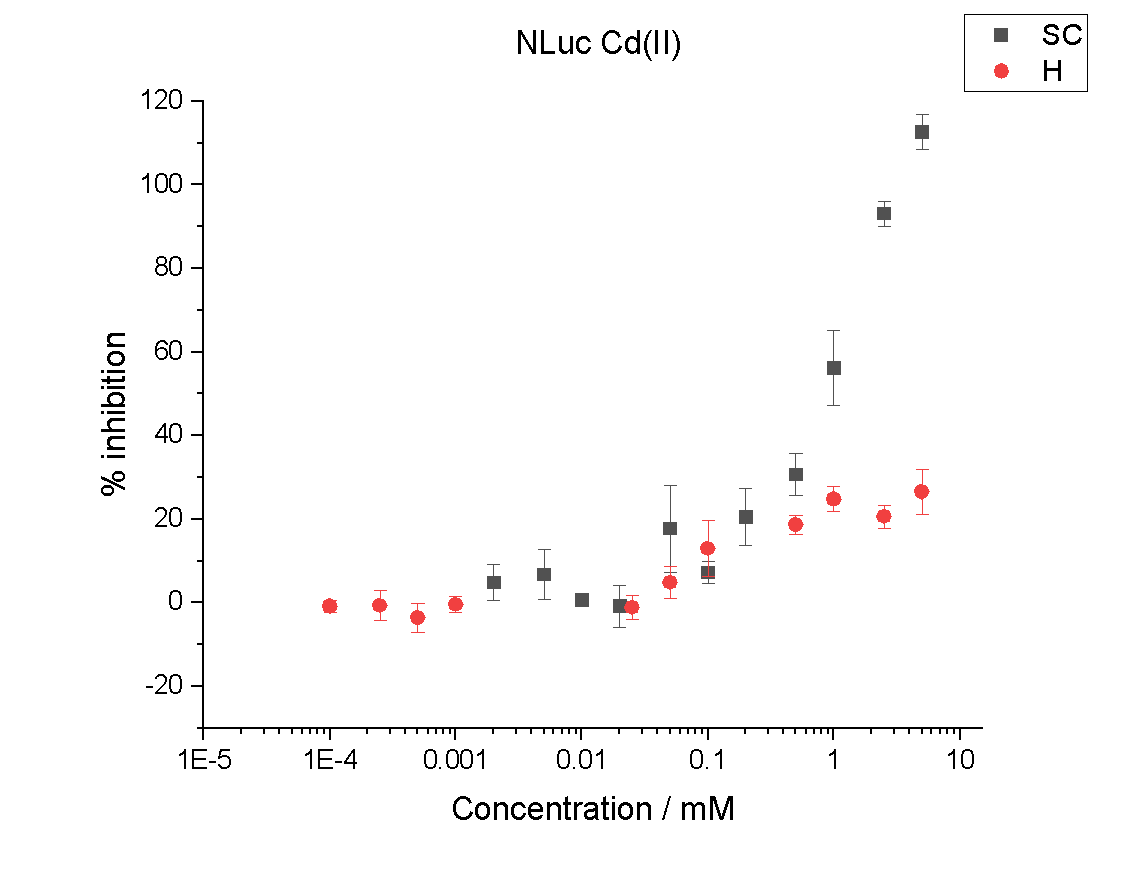 | 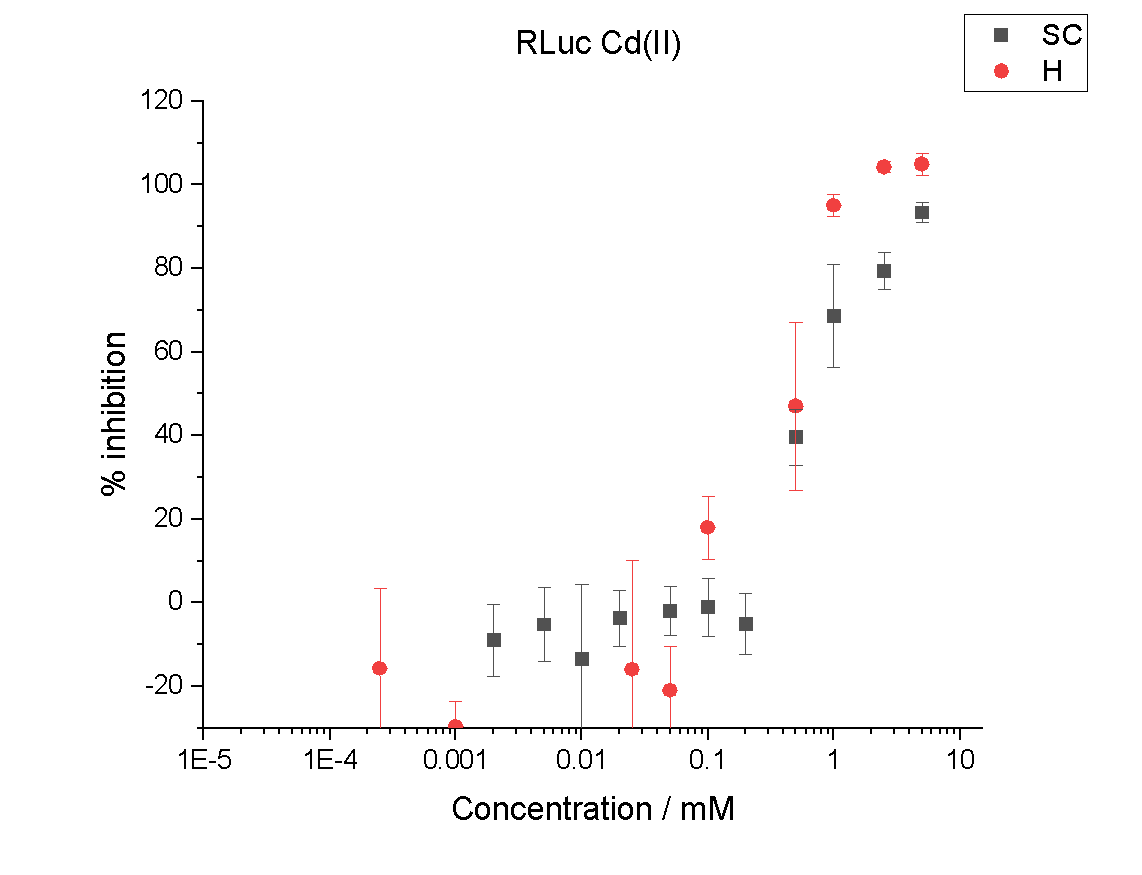 |
| Co(II) | 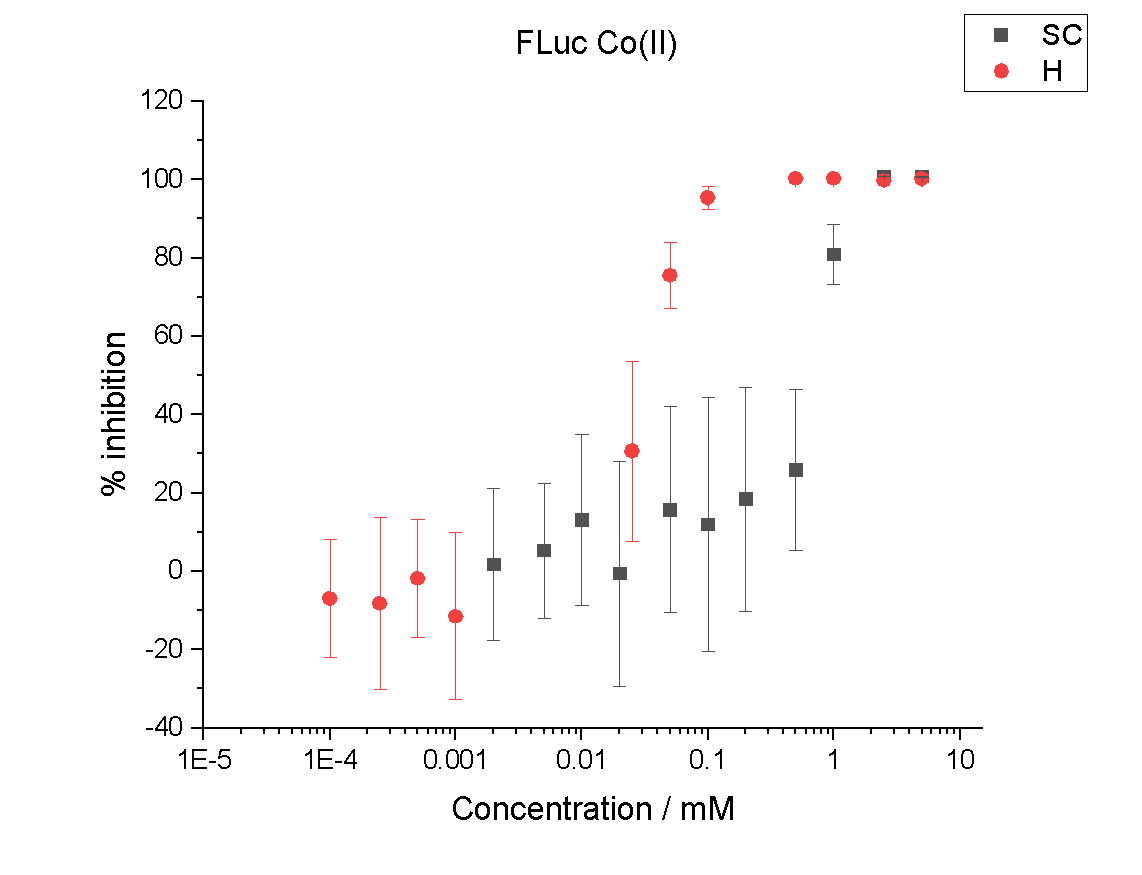 | 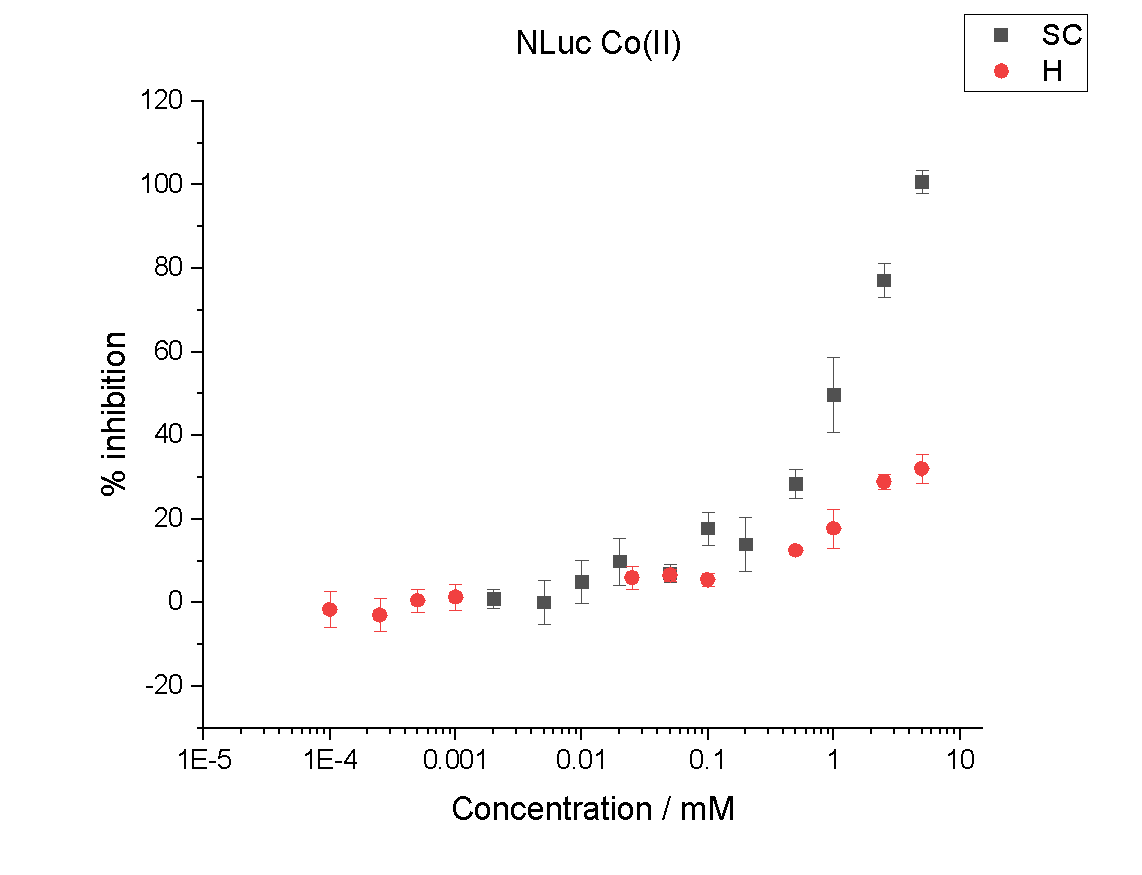 | 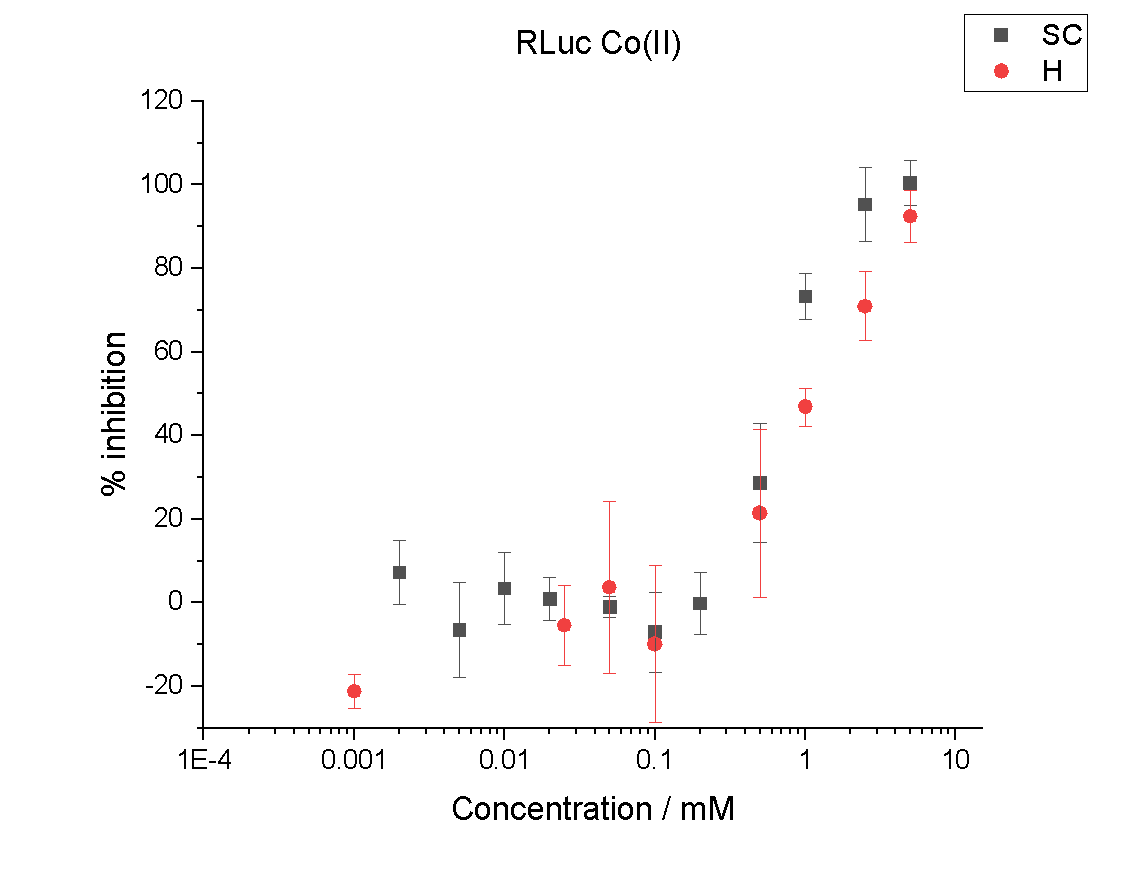 |
| Cu(I) | 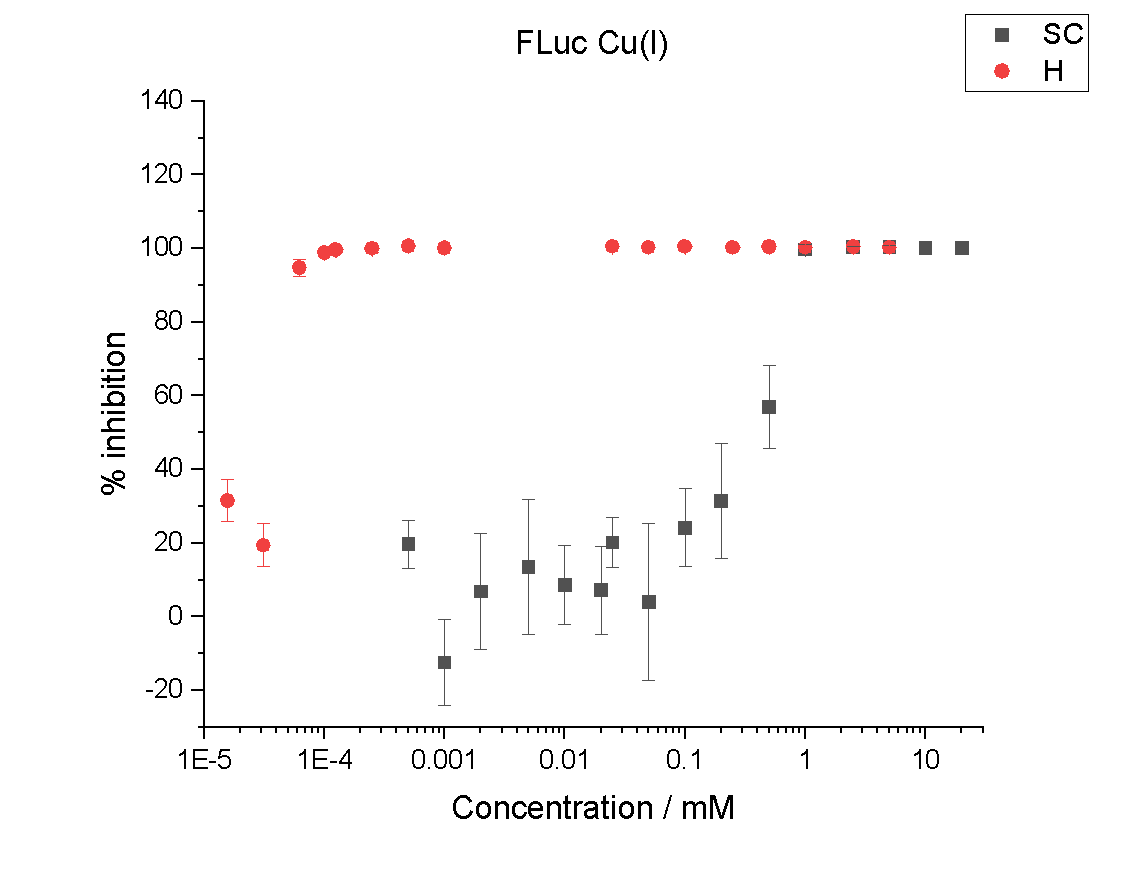 | 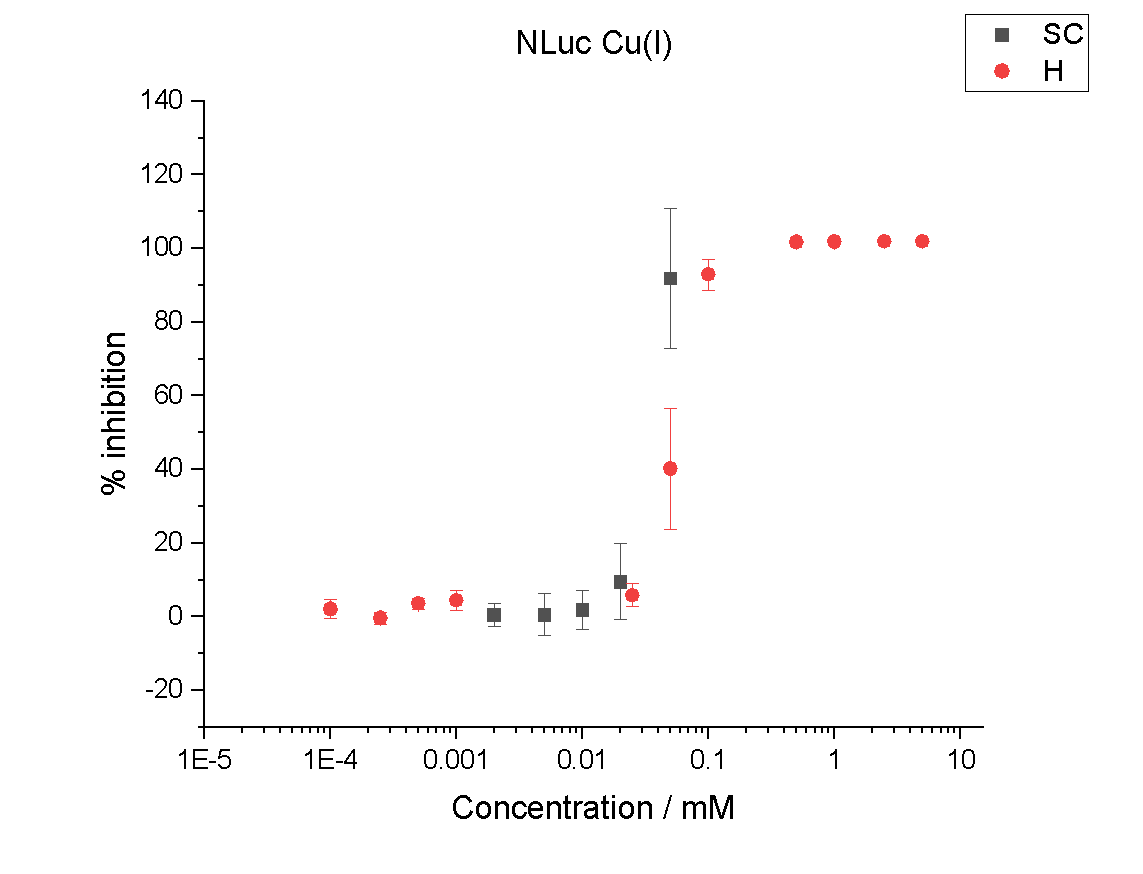 | 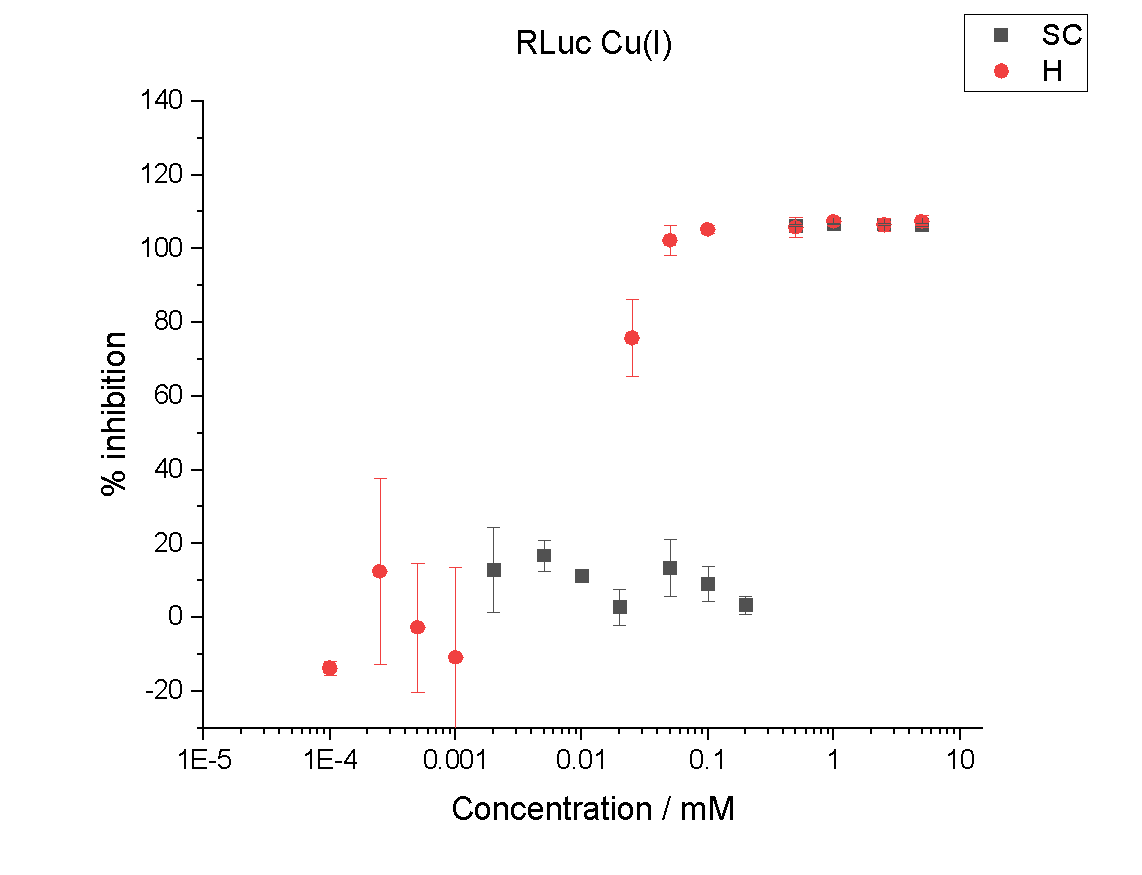 |
| Cu(II) | 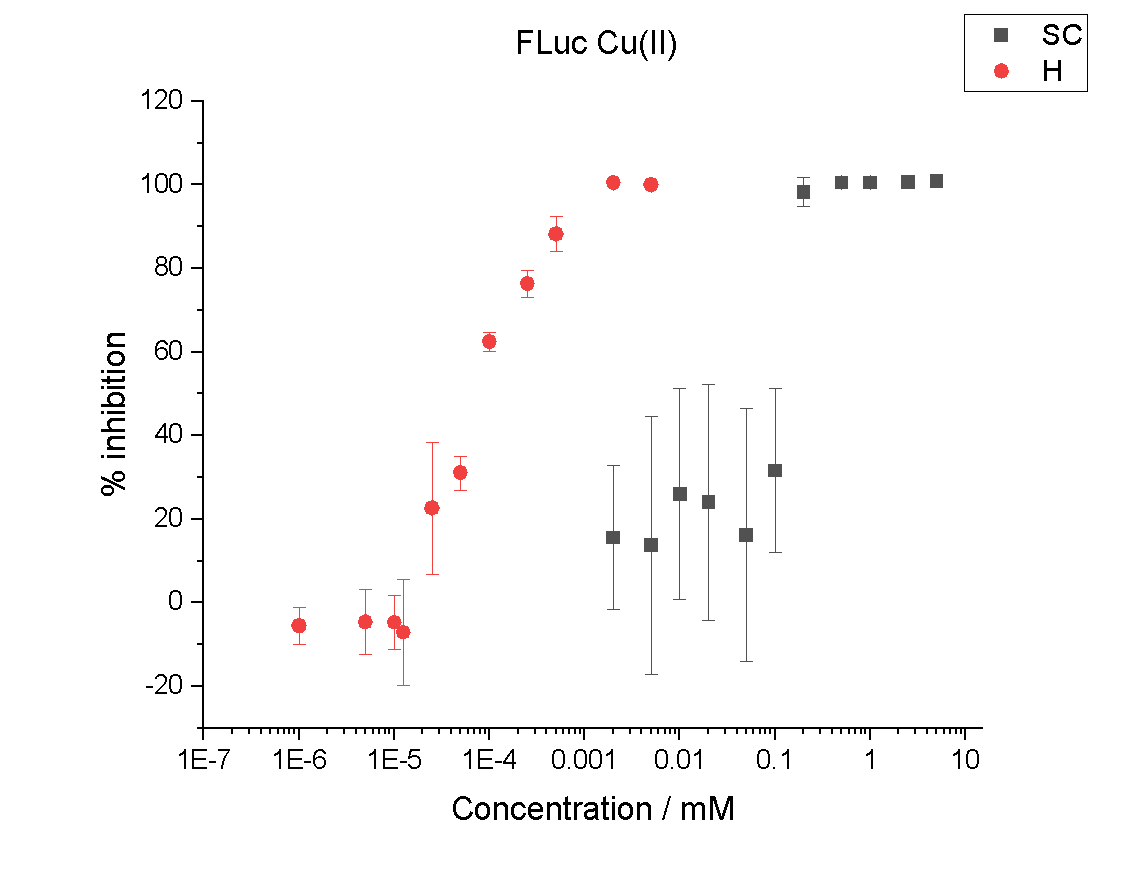 | 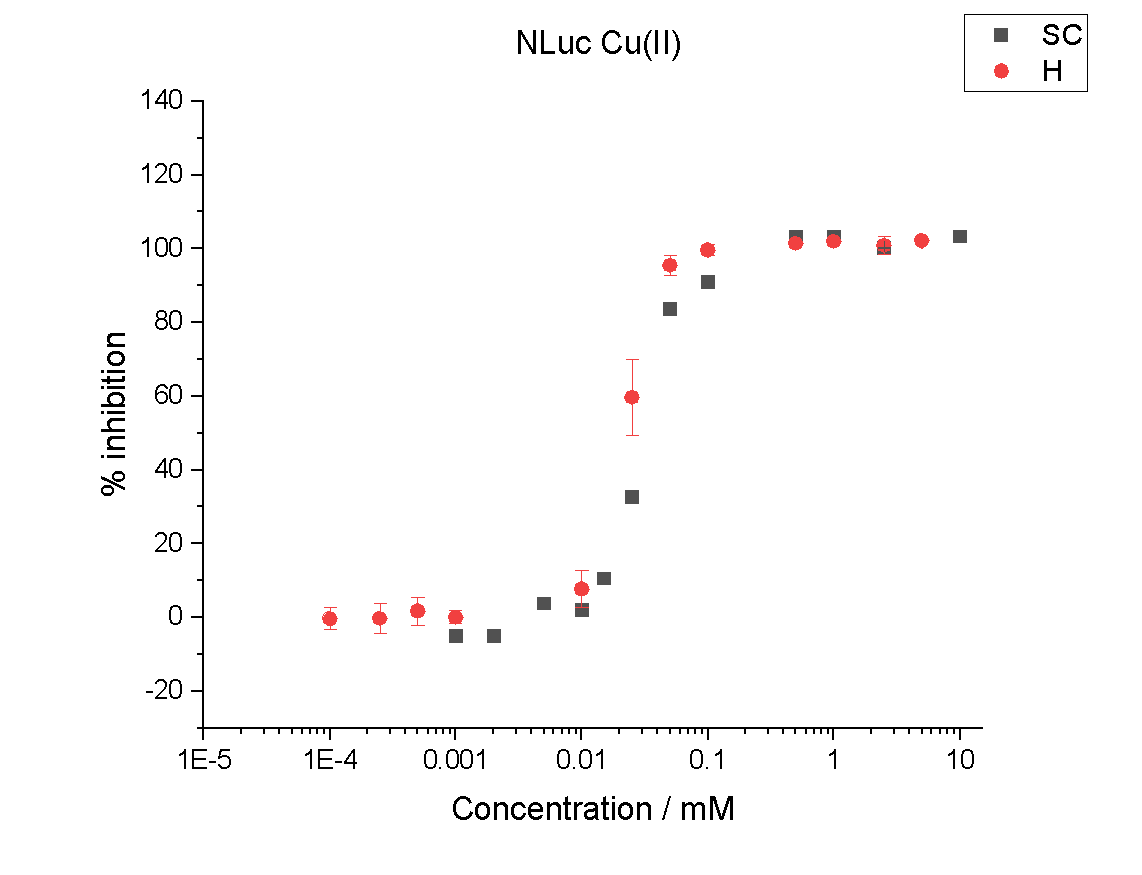 | 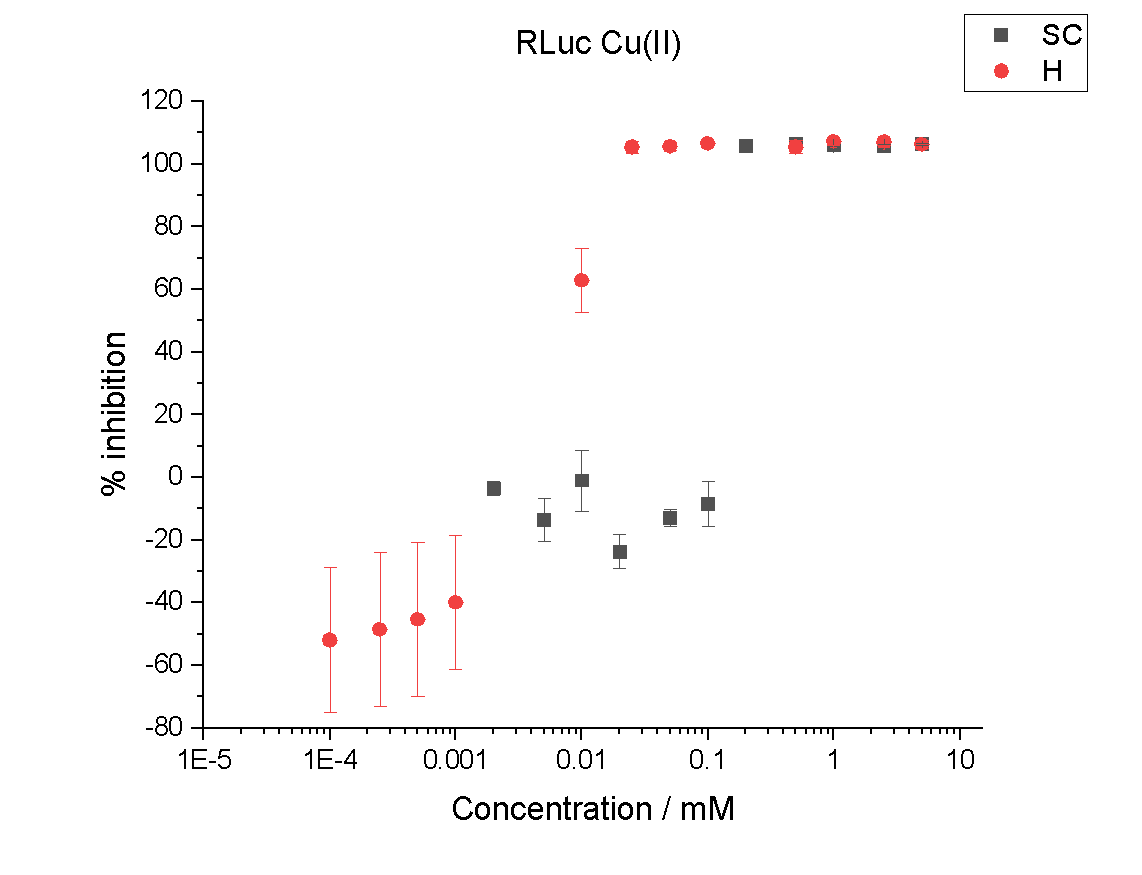 |
| Fe(II)-1 | 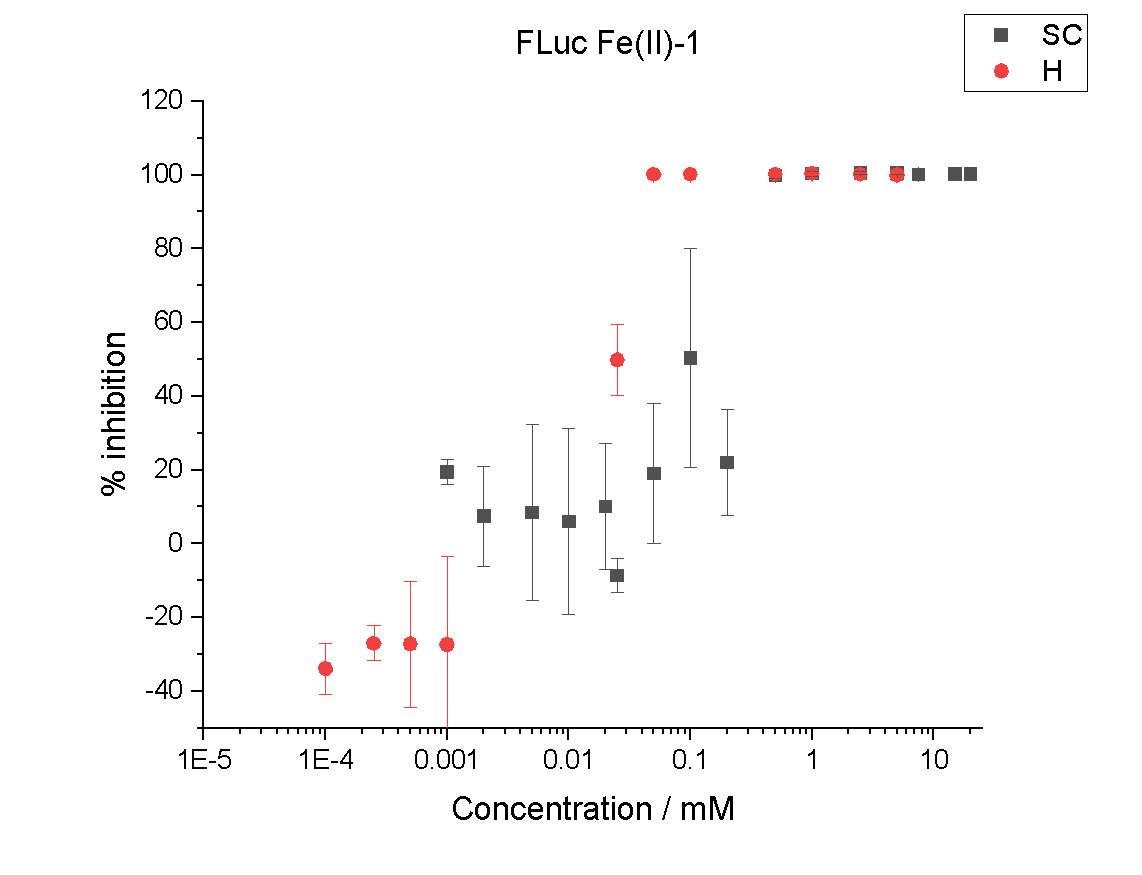 | 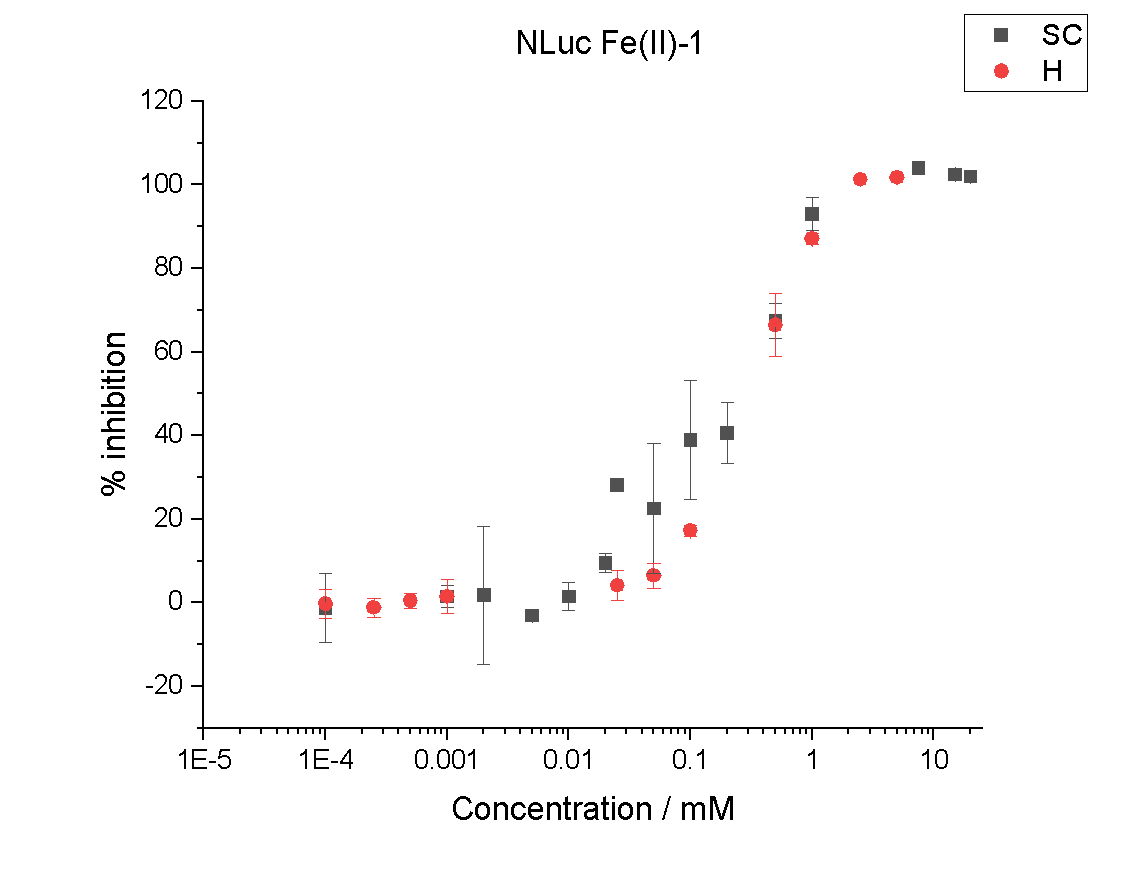 | 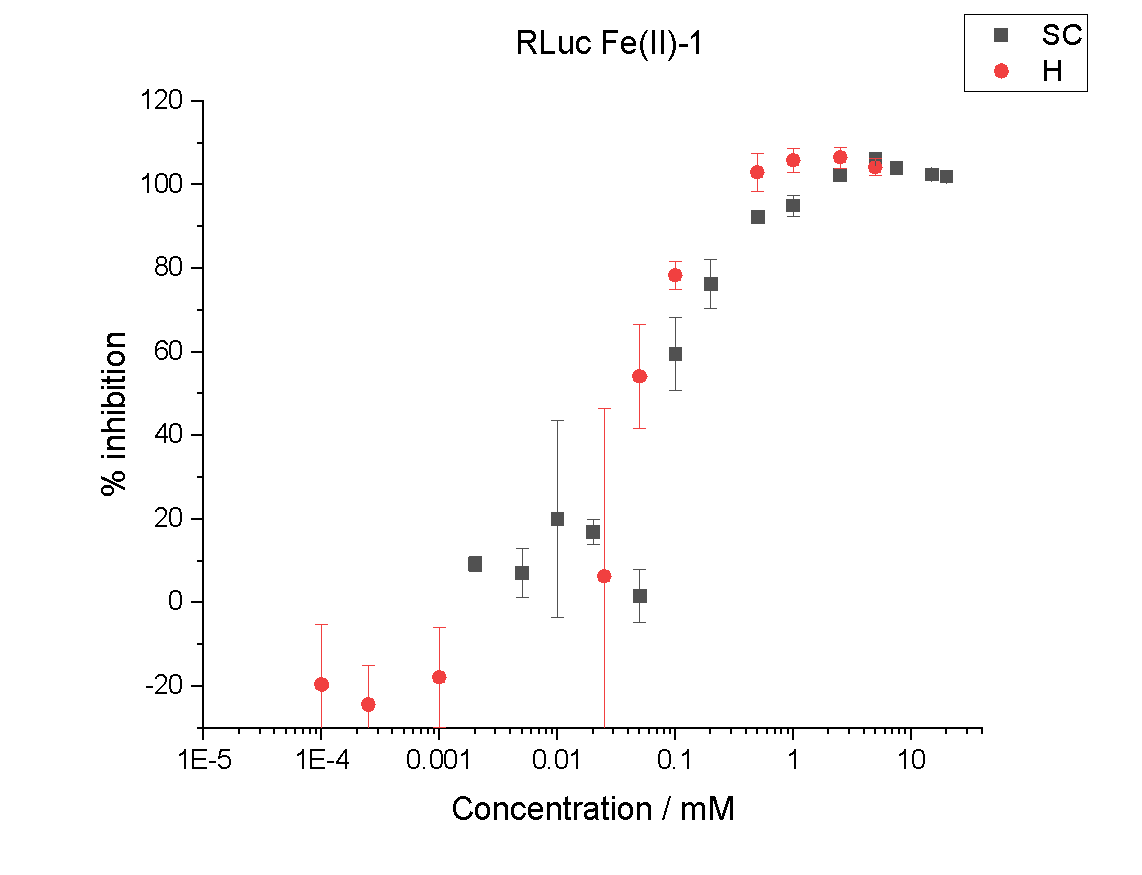 |
| Fe(II)-2 | 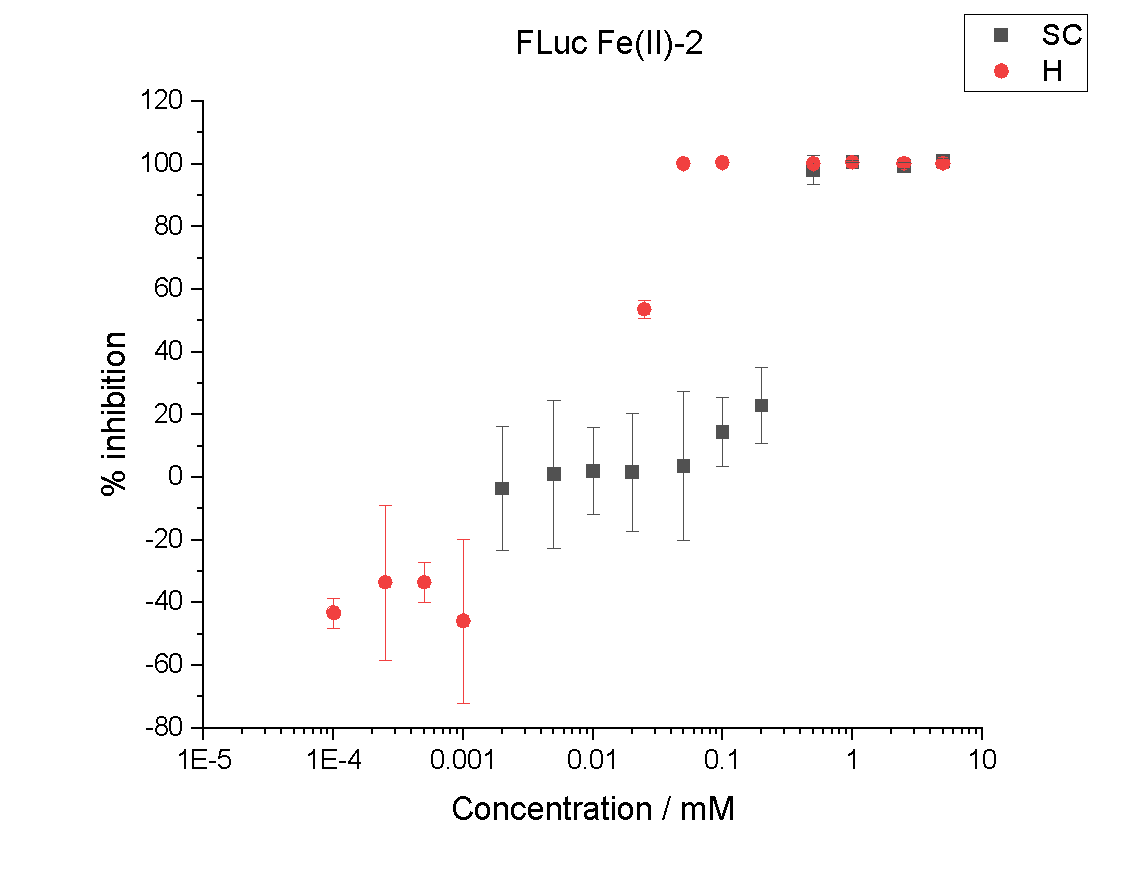 | 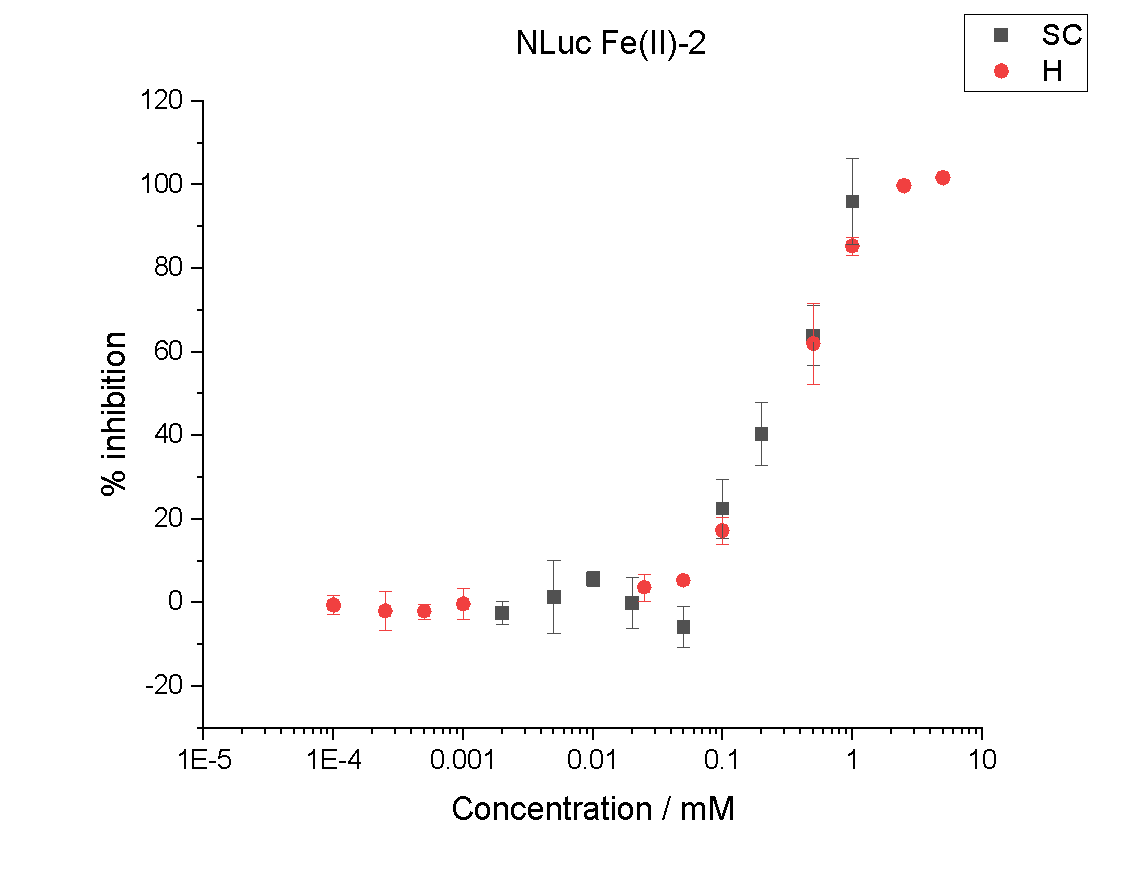 | 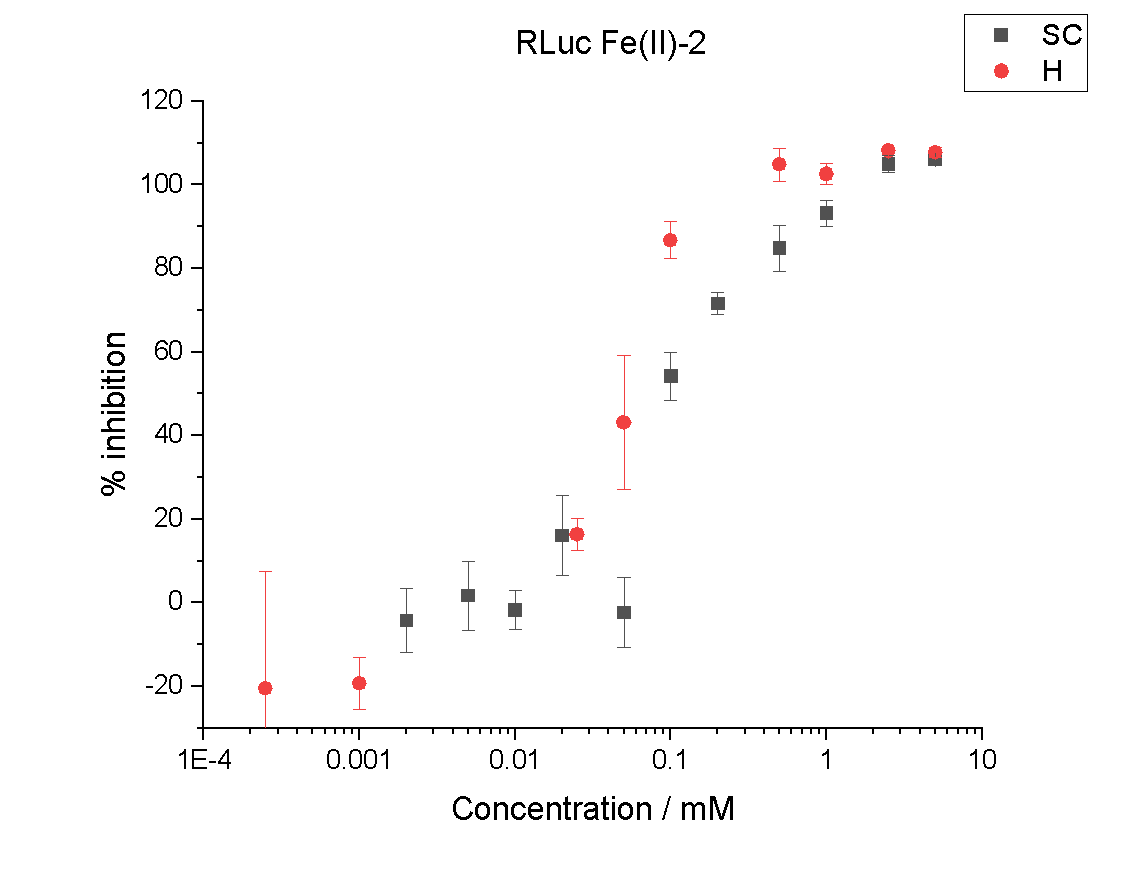 |
| Fe(II)-3 | 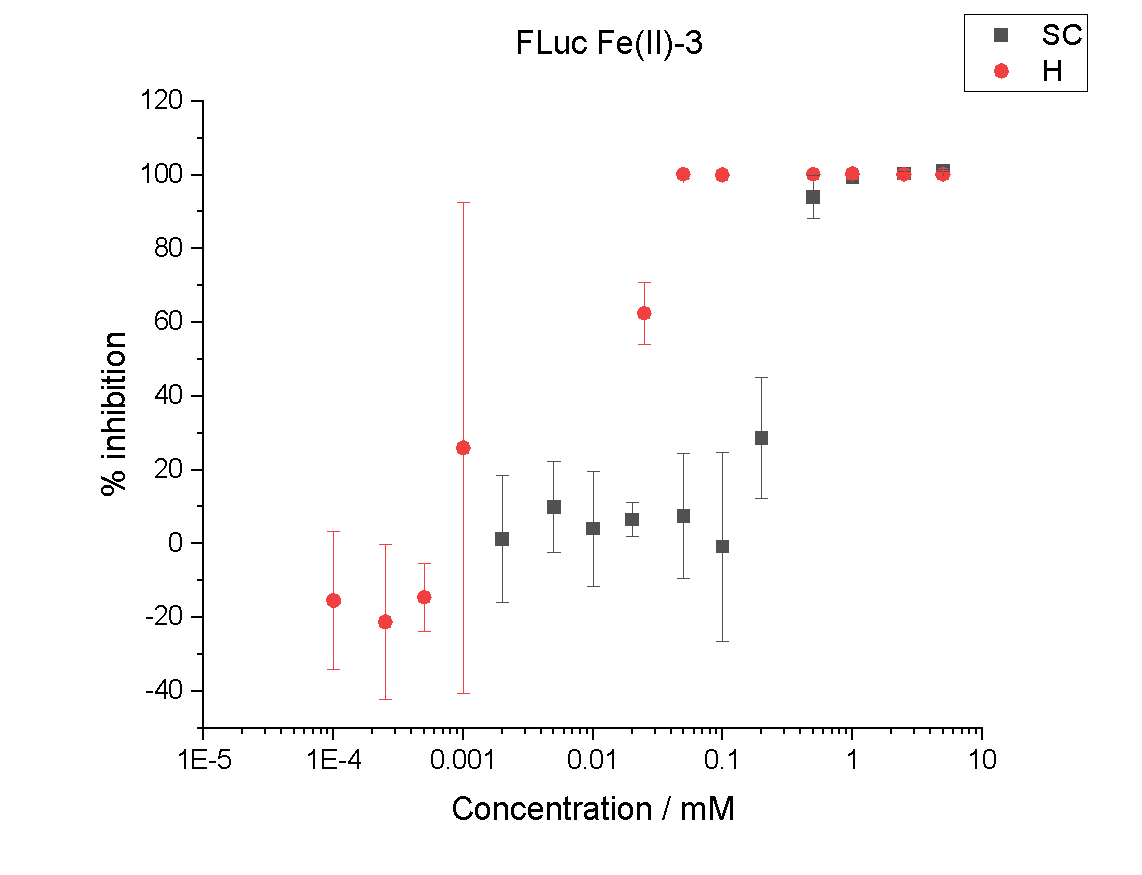 | 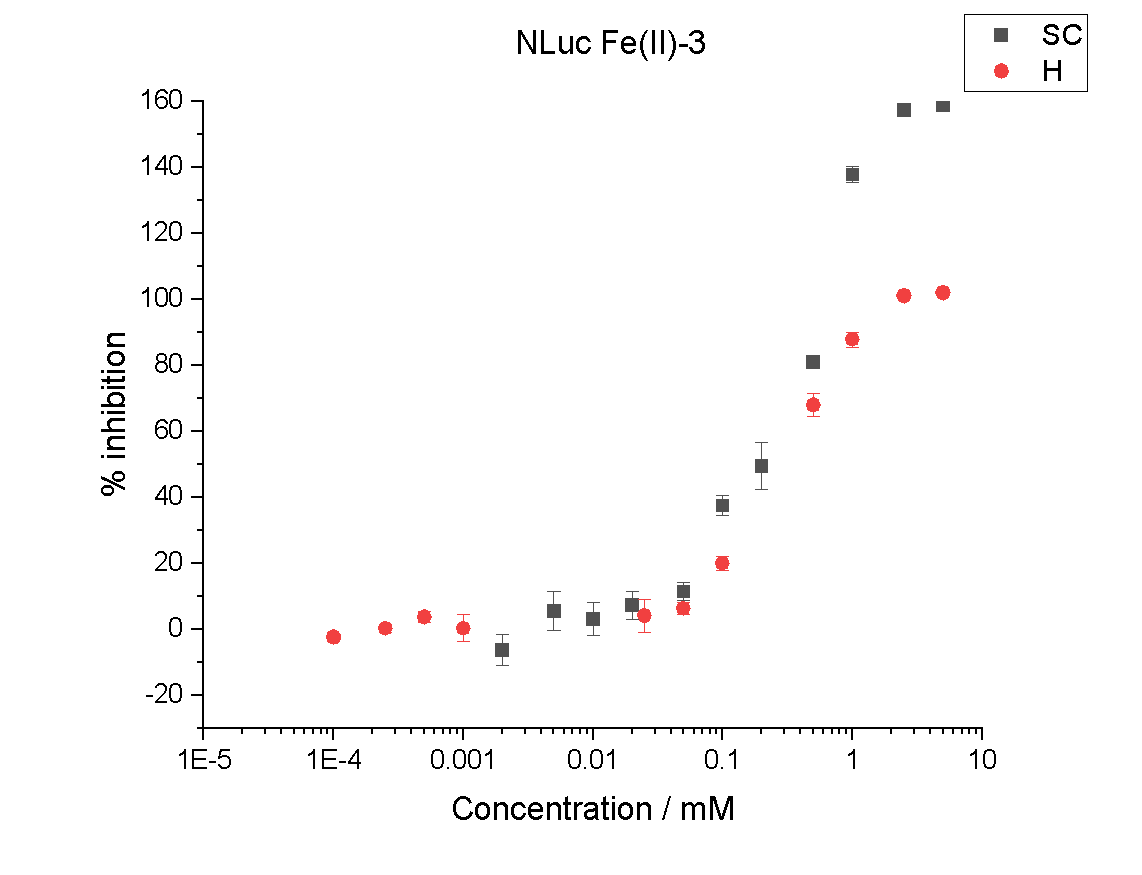 | 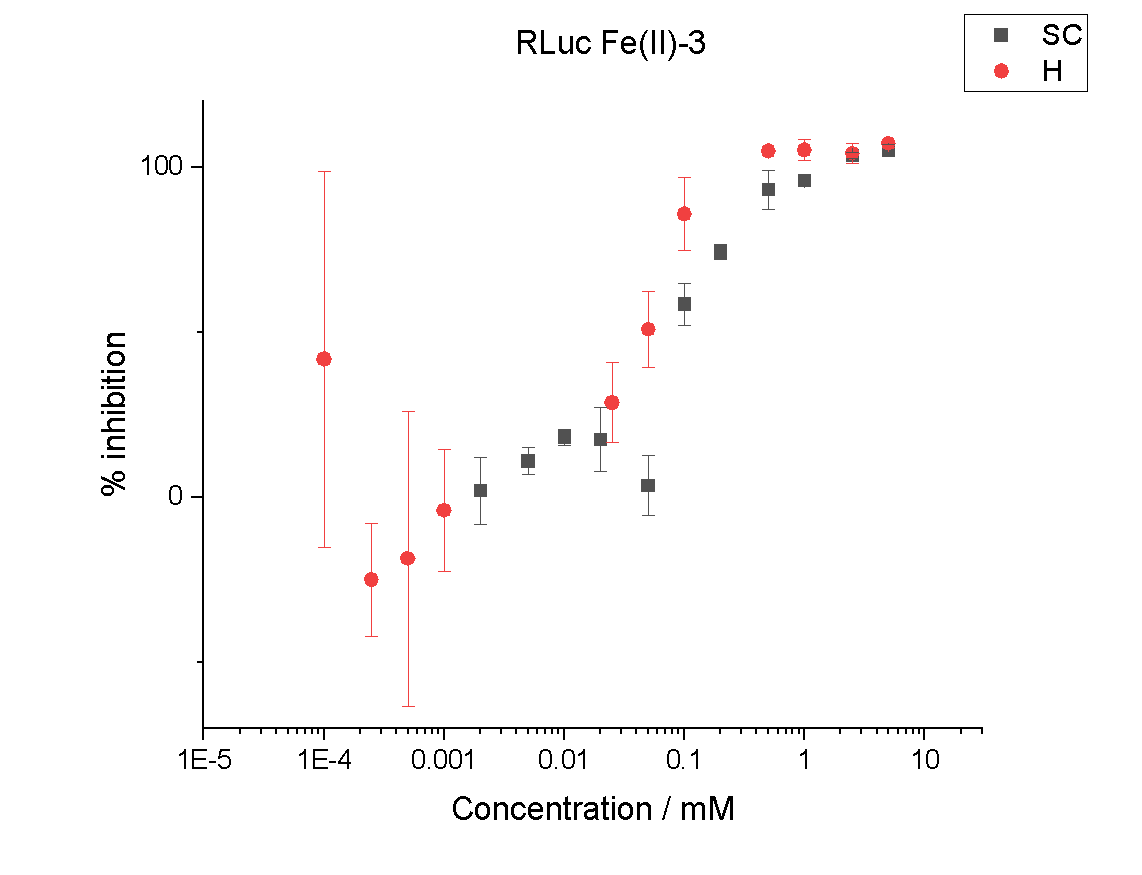 |
| Fe(III) | 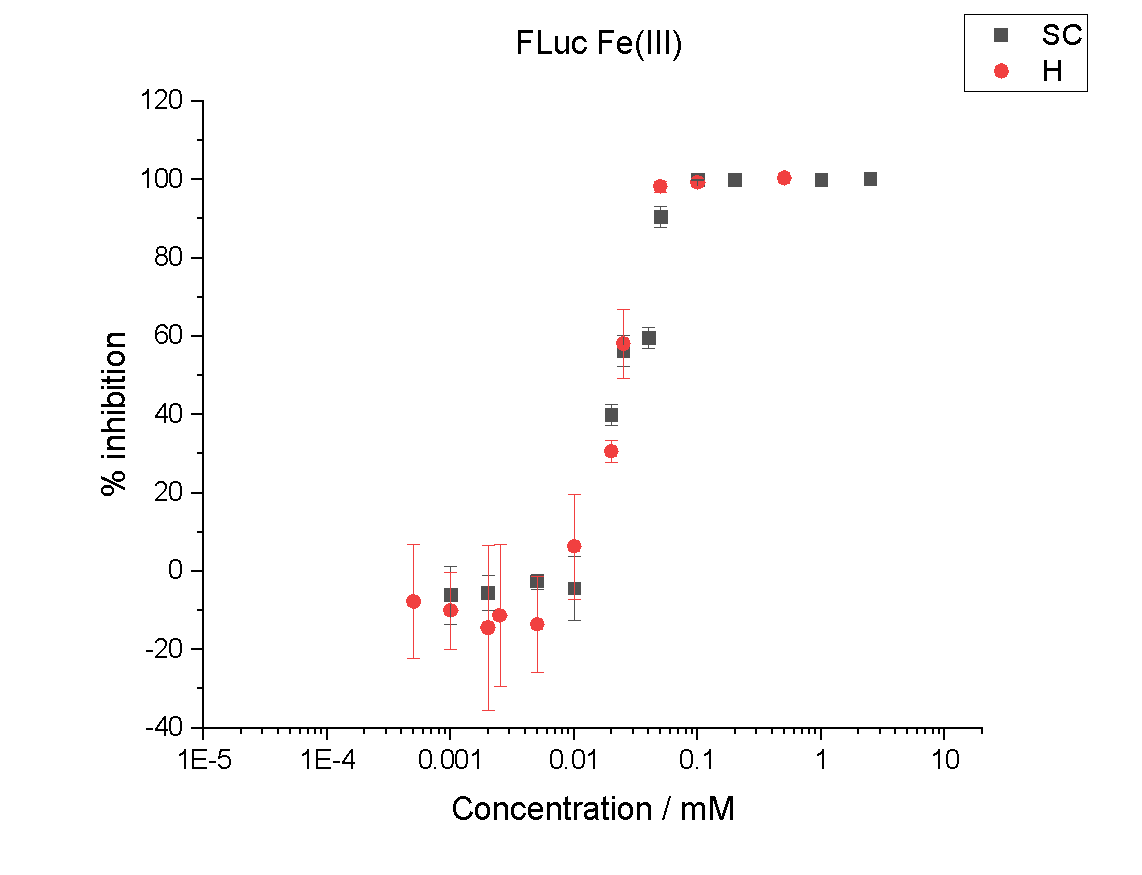 | 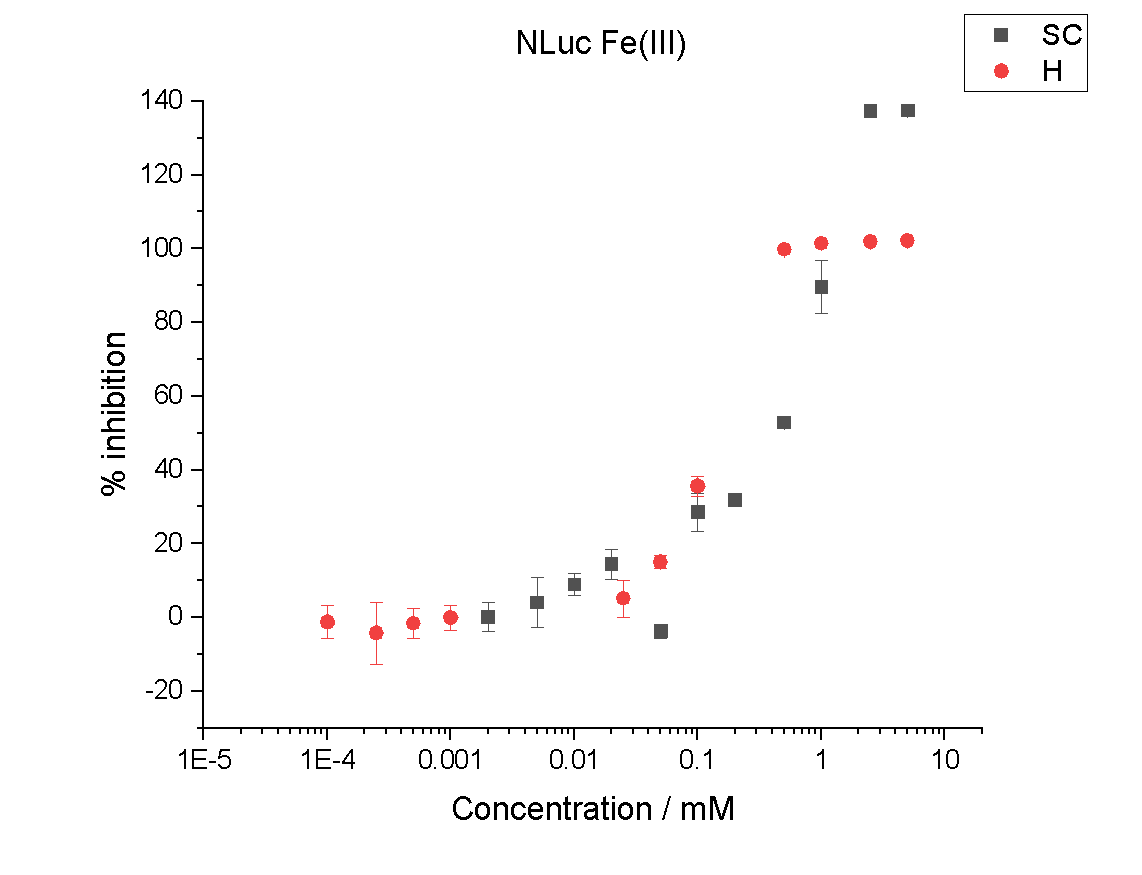 | 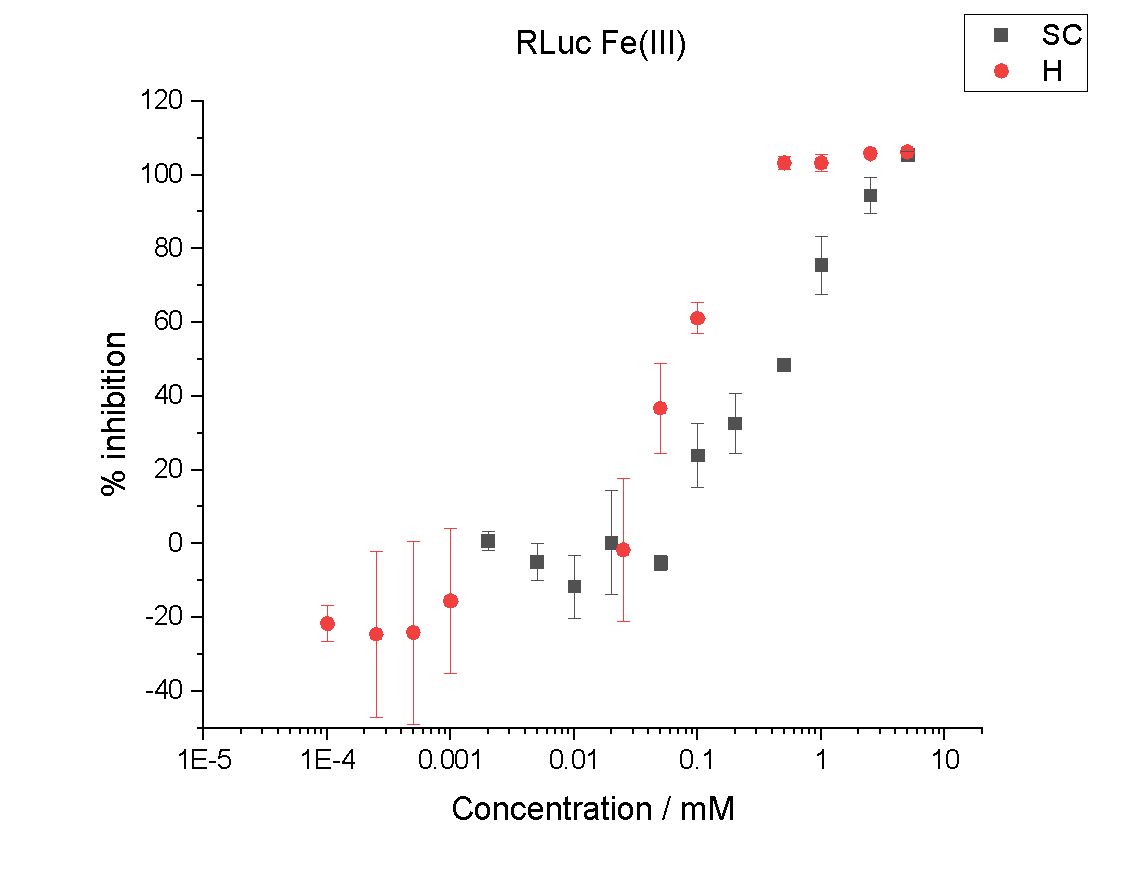 |
| Ga | 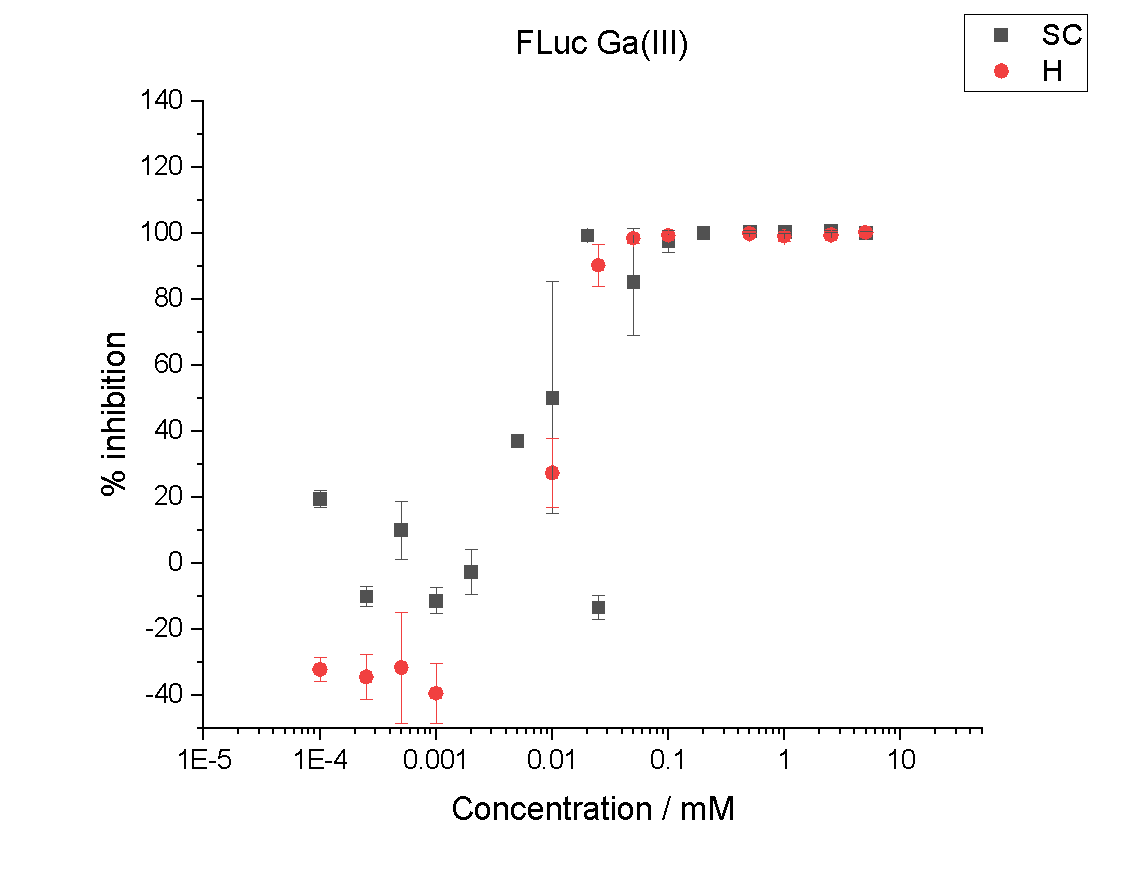 | 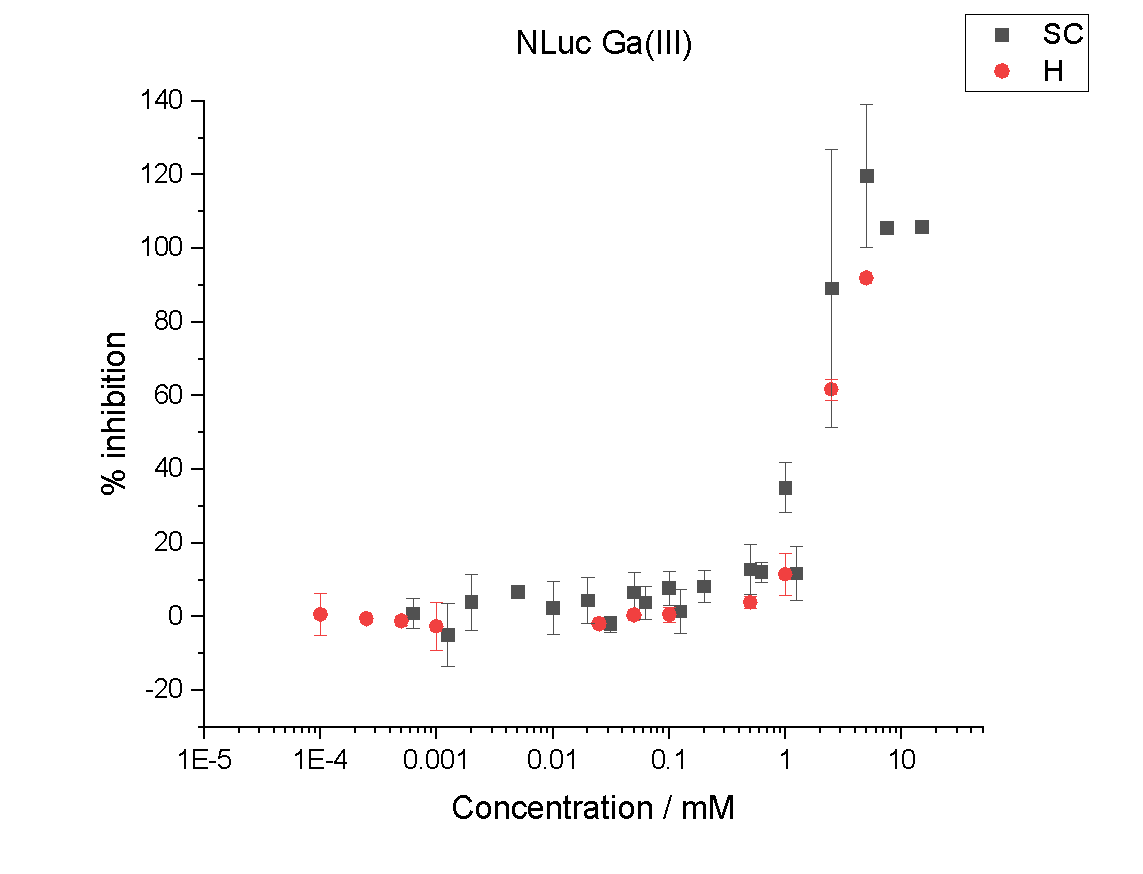 | 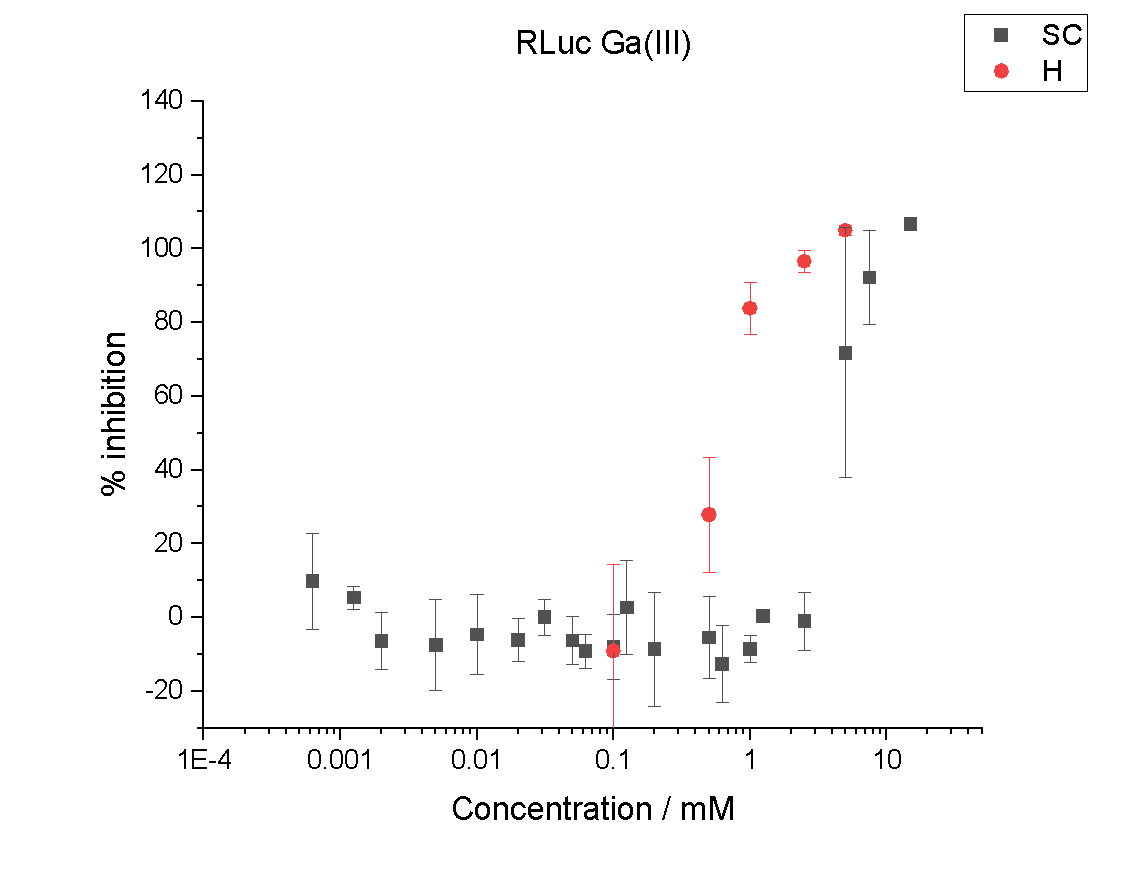 |
| Pb(II) | 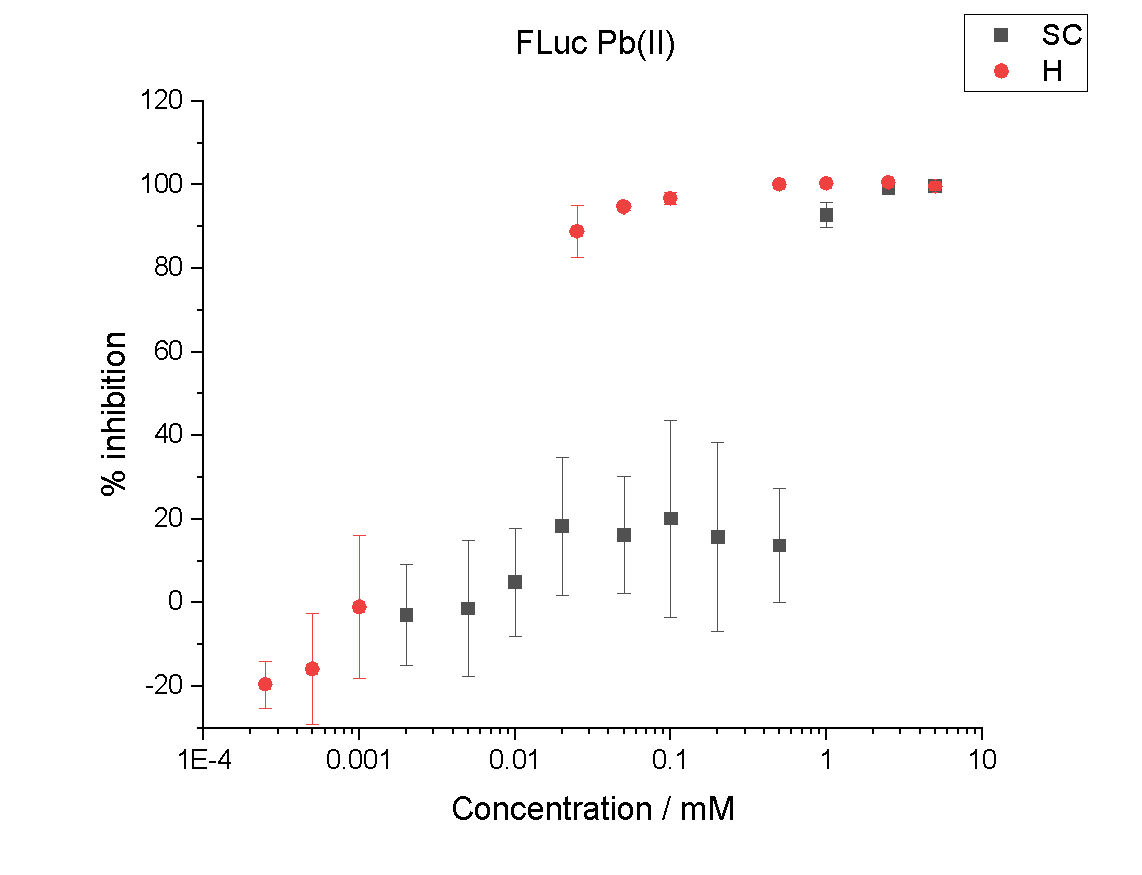 | 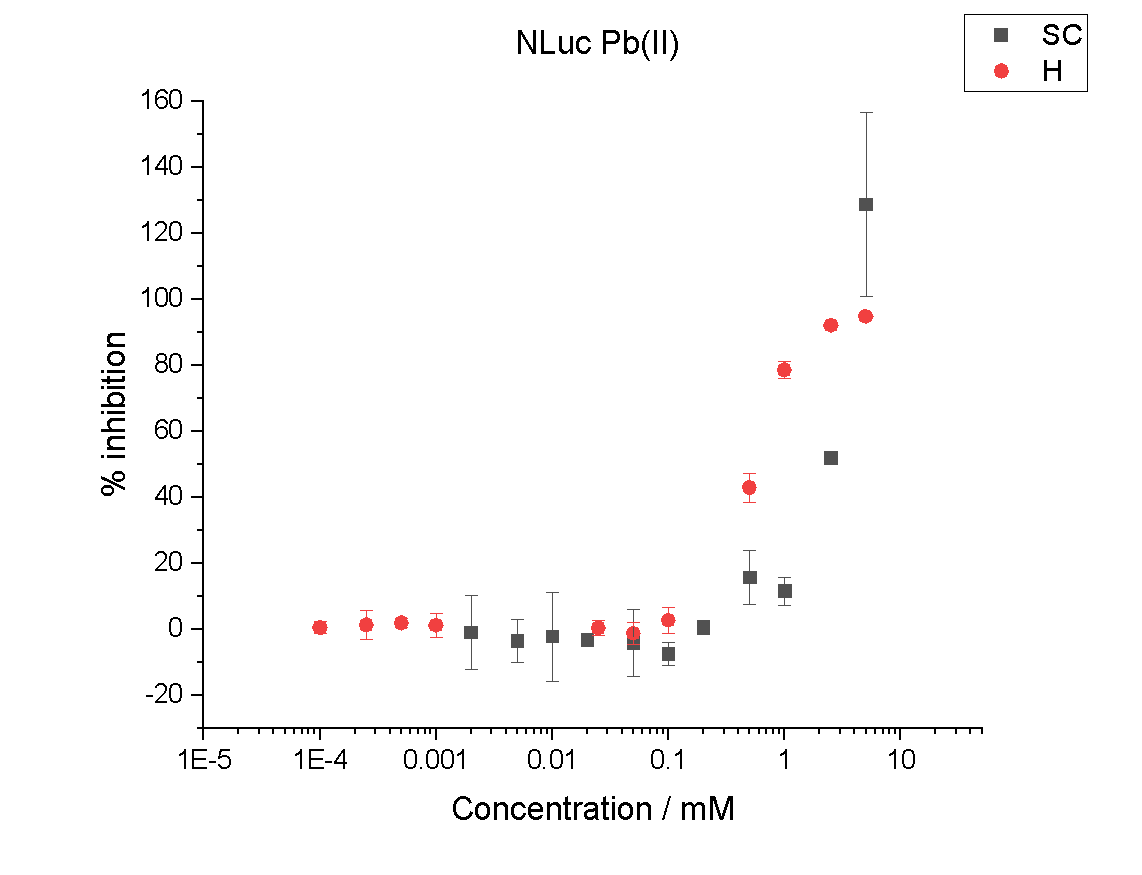 | 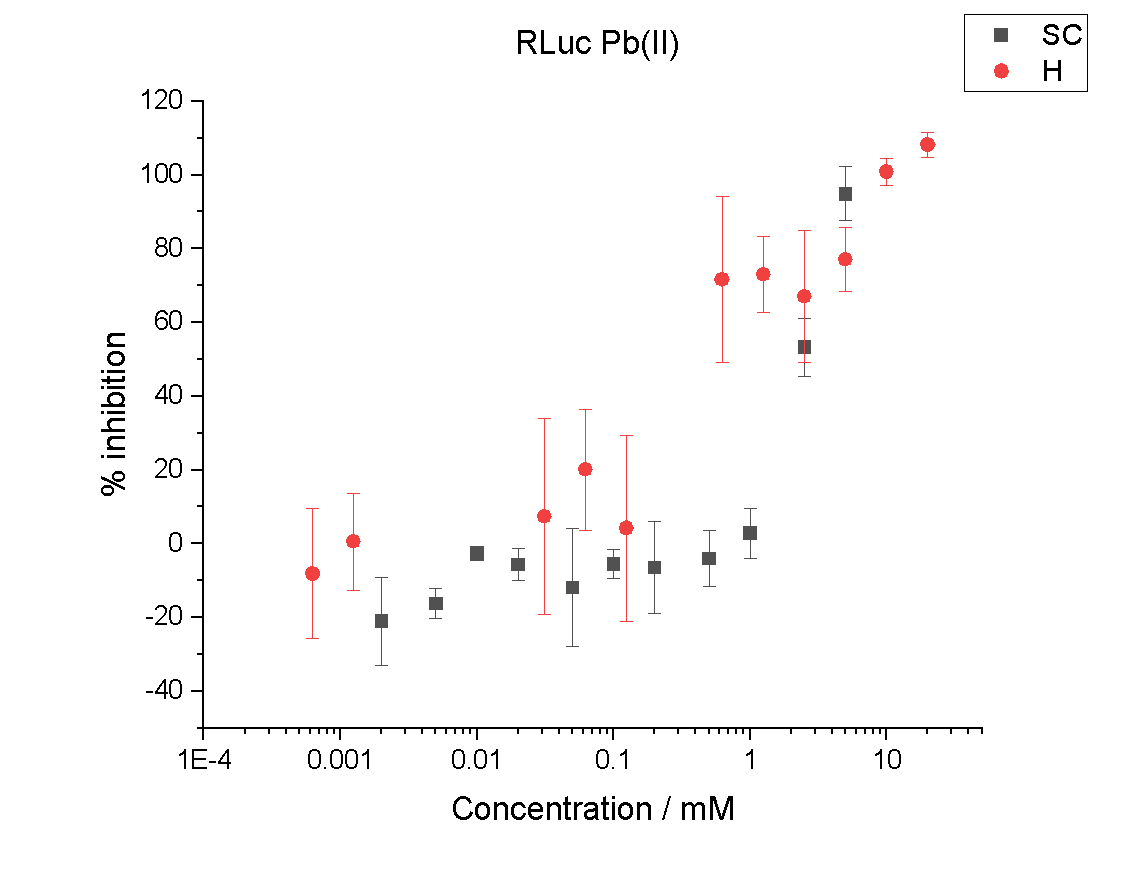 |
| Pt(II) | 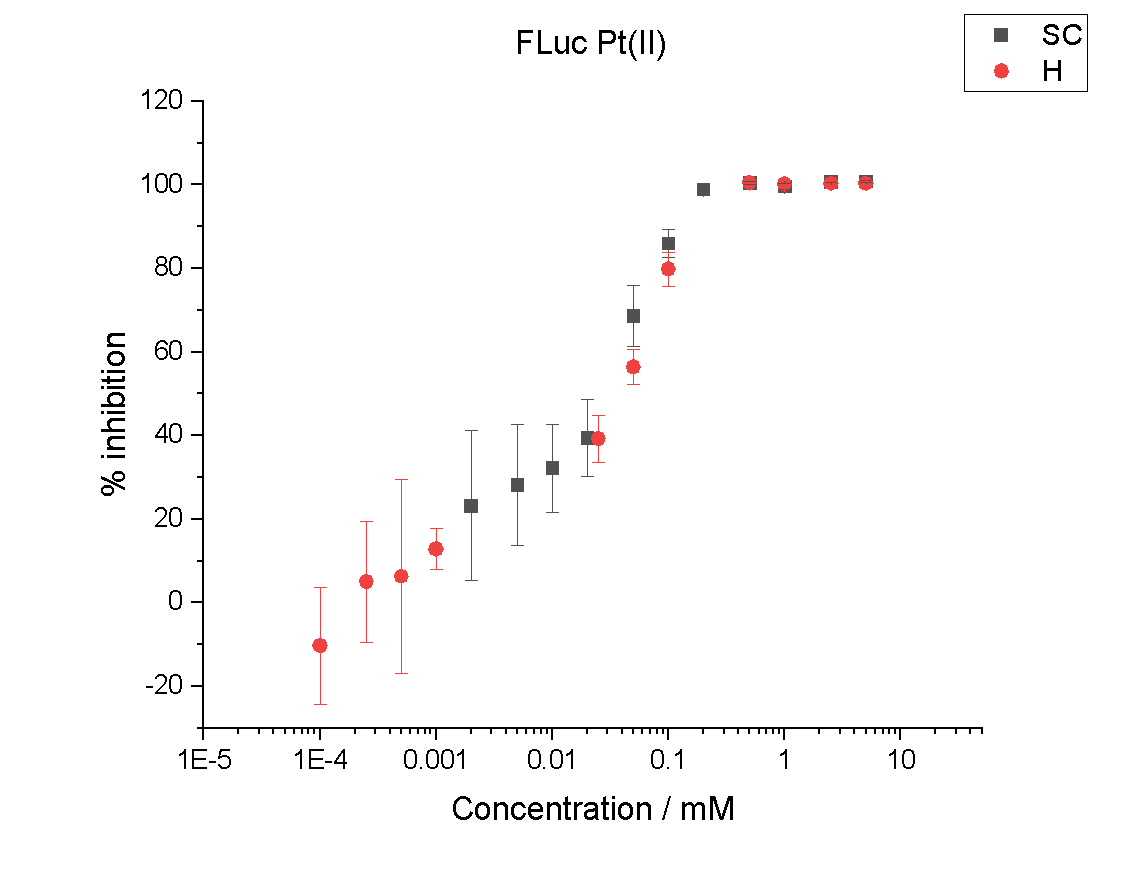 | 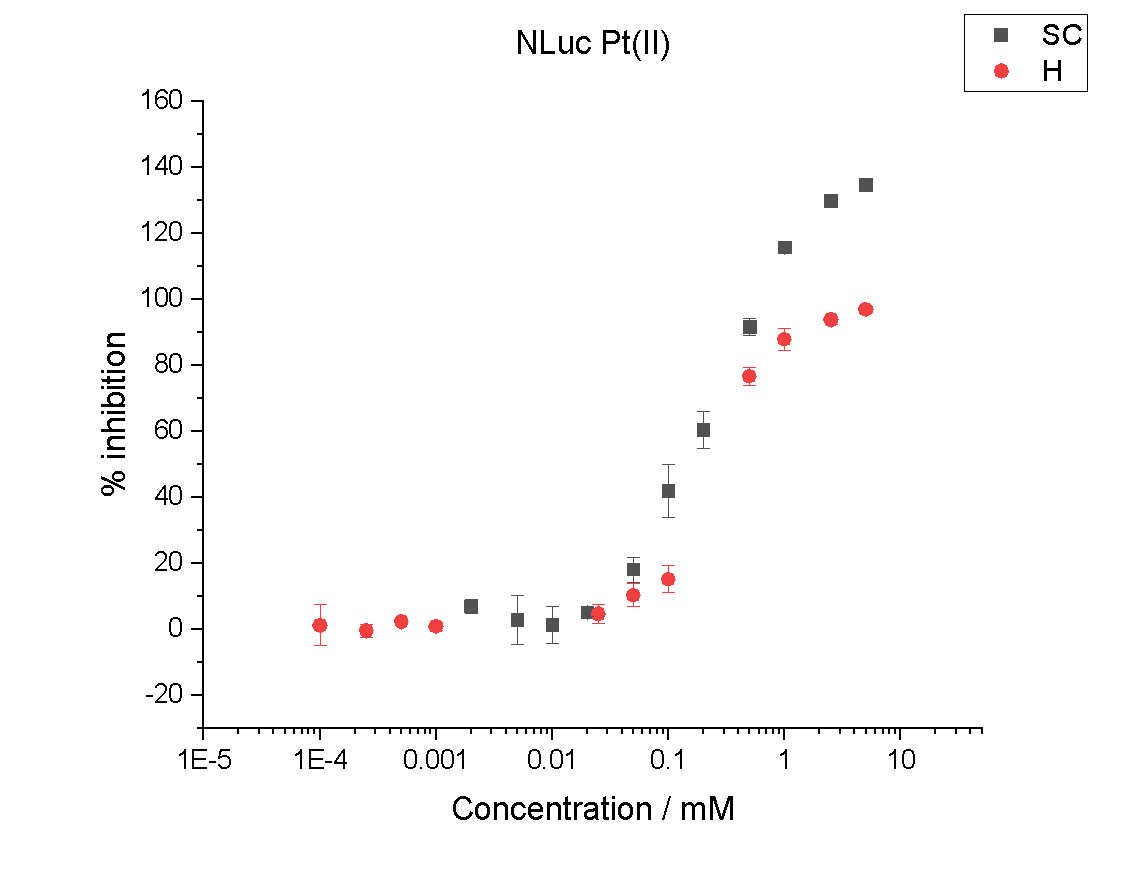 | 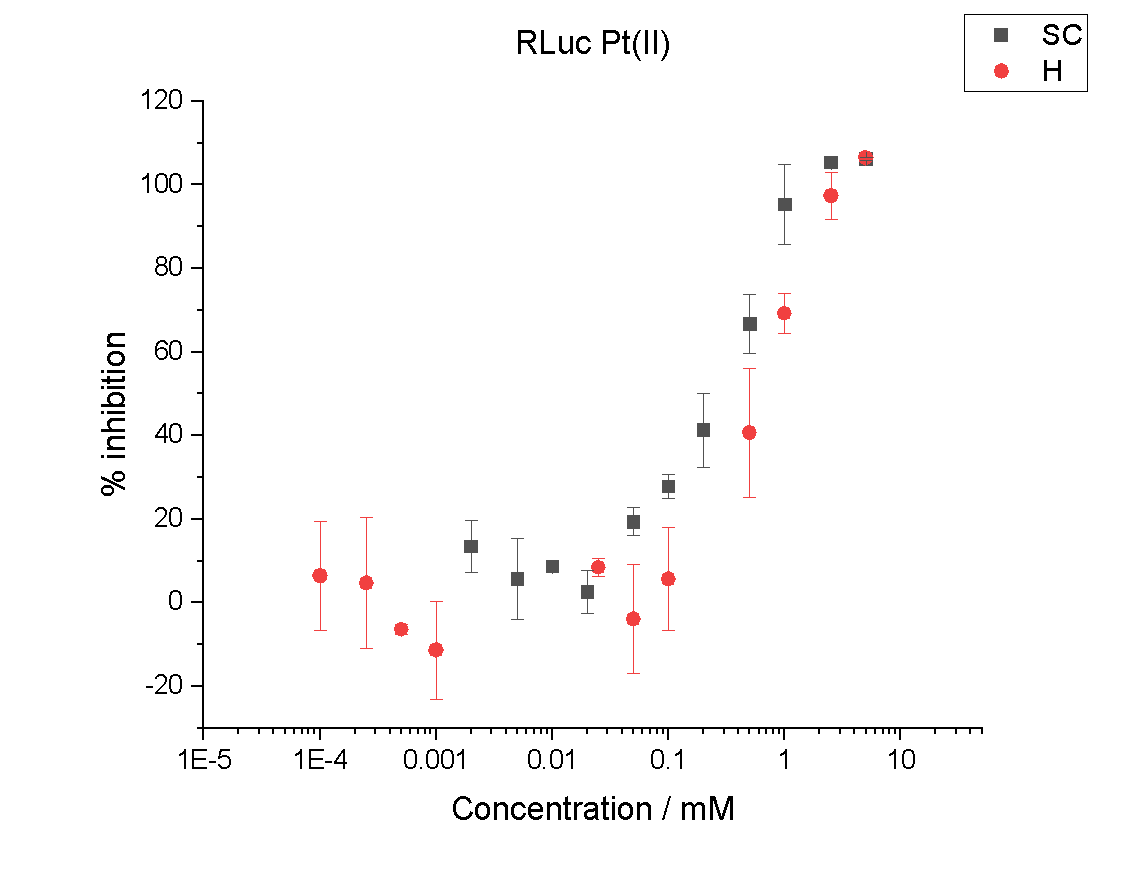 |
| Sn(II) | 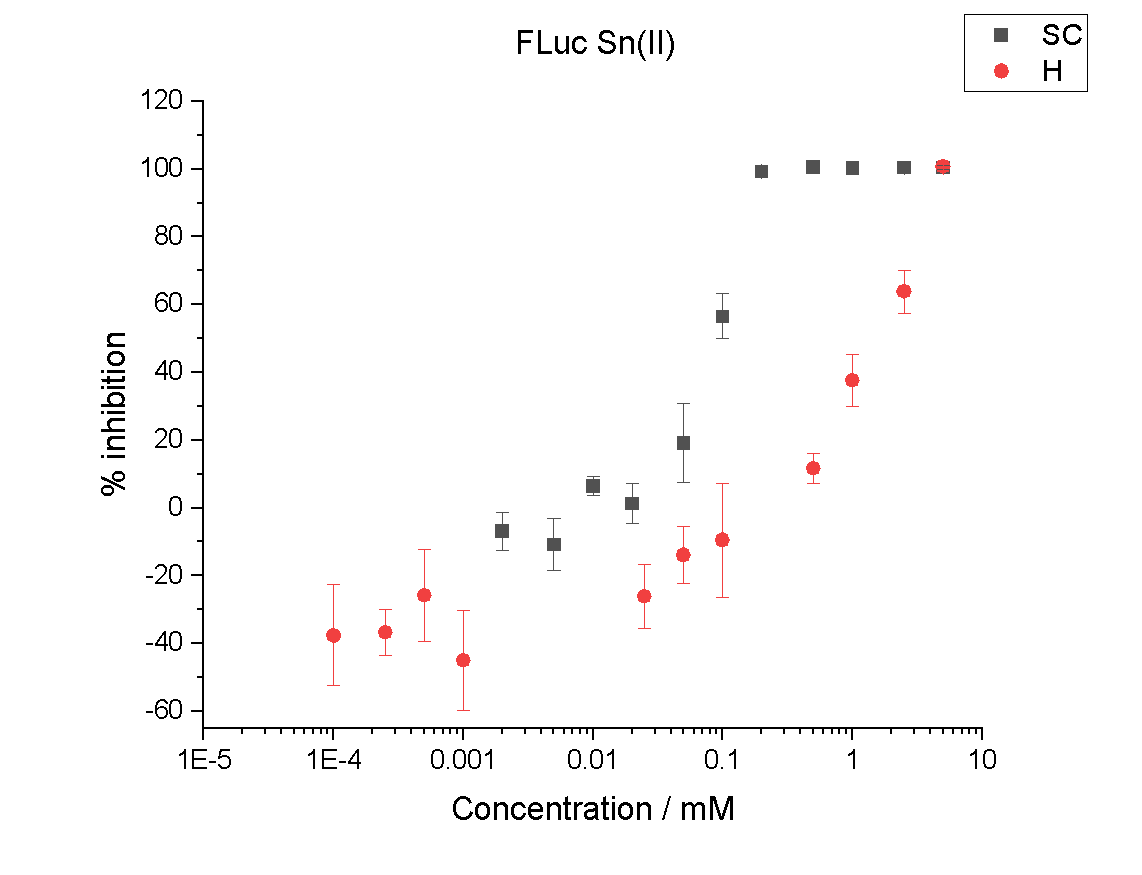 | 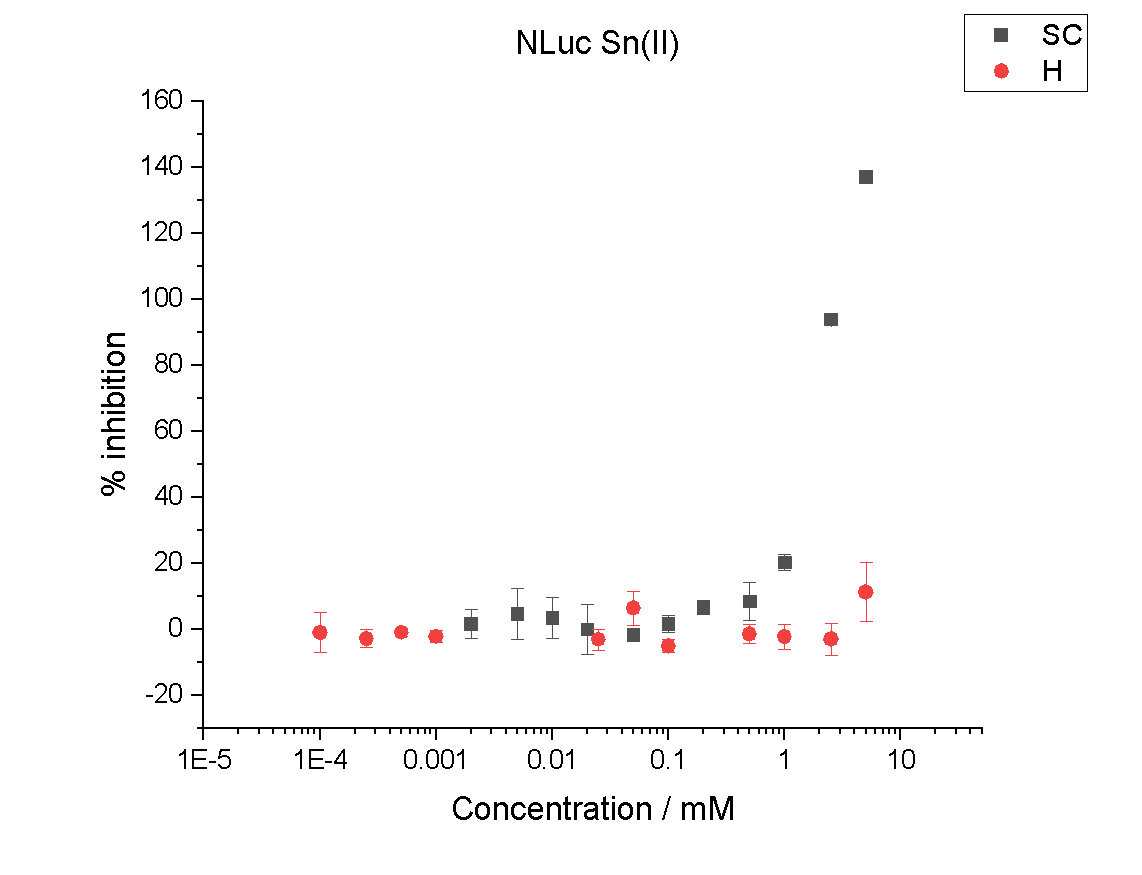 | 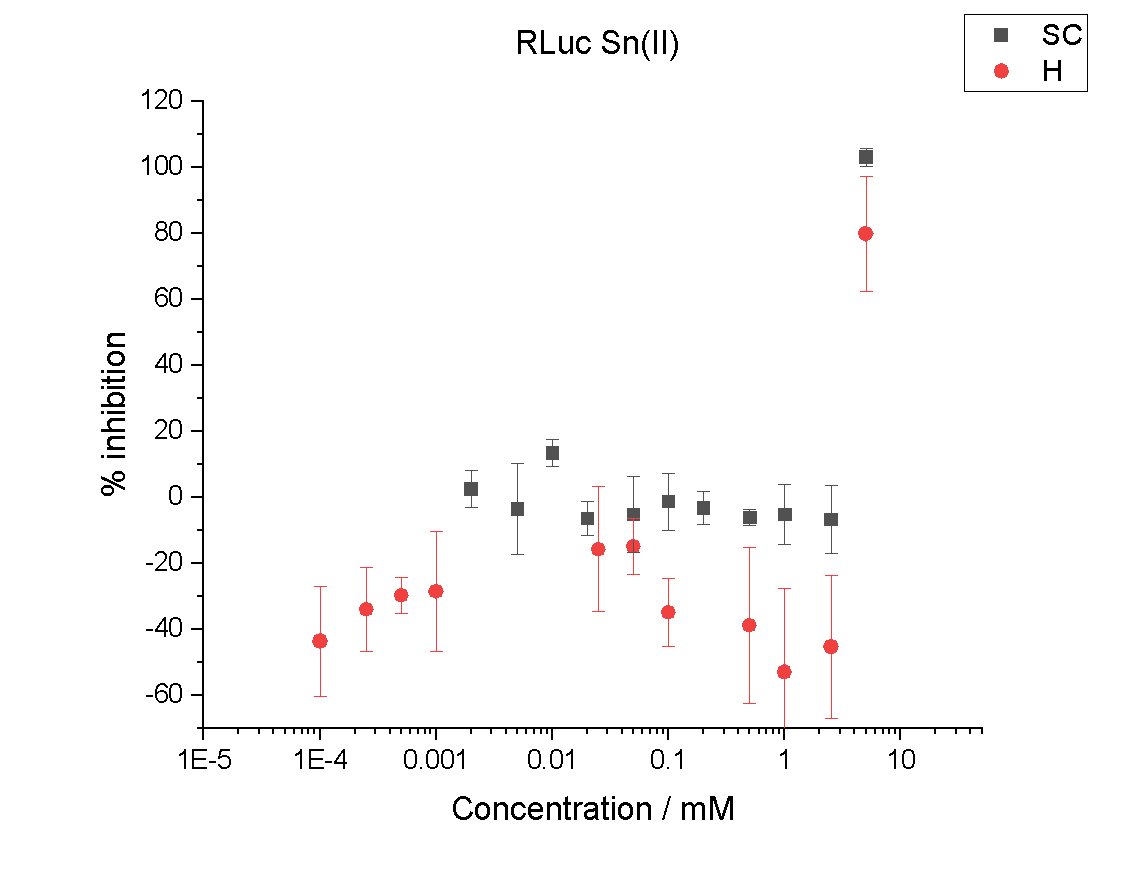 |
| Zn | 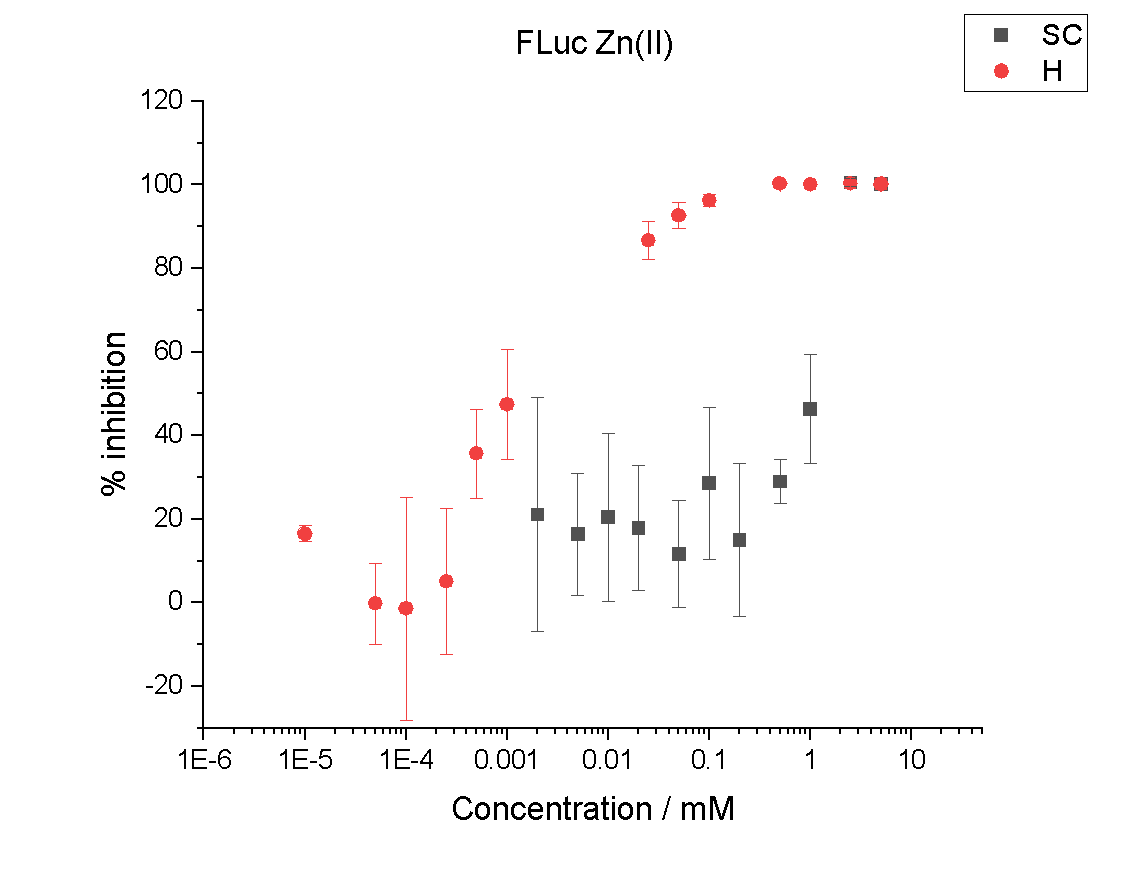 | 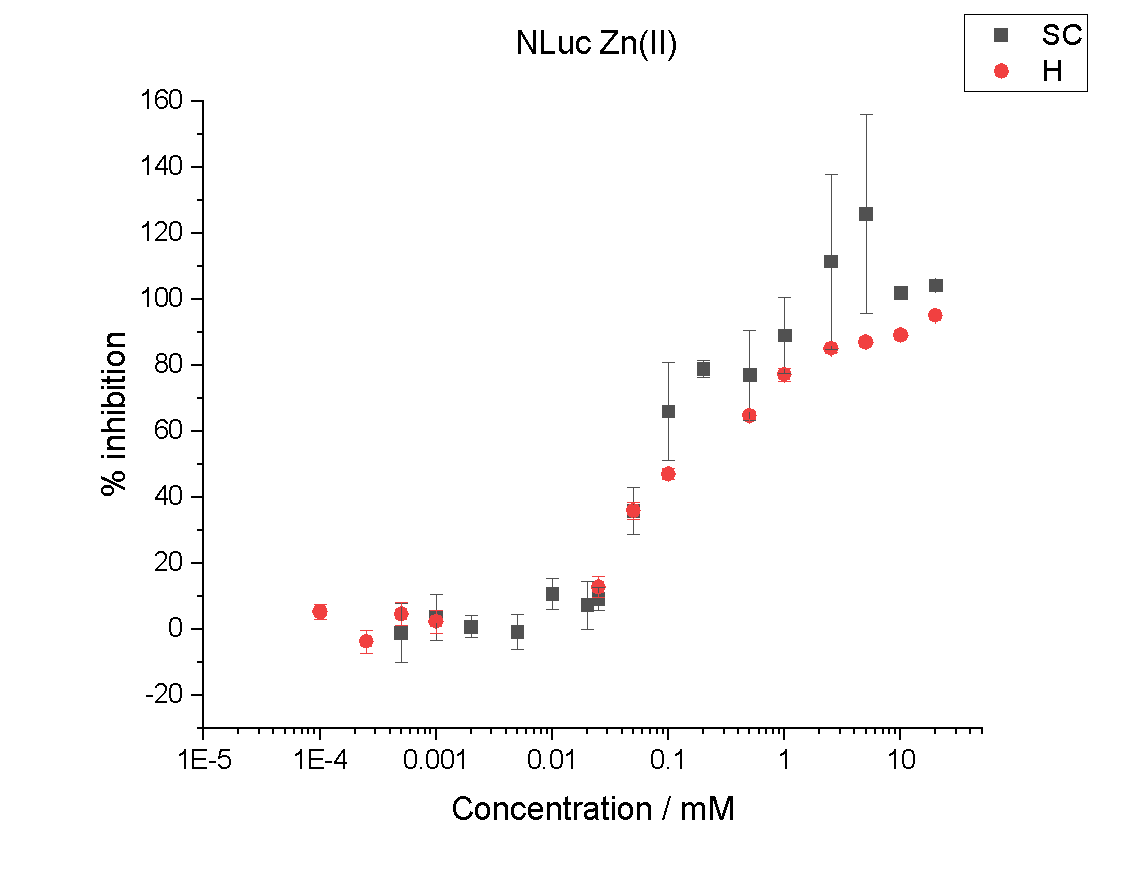 | 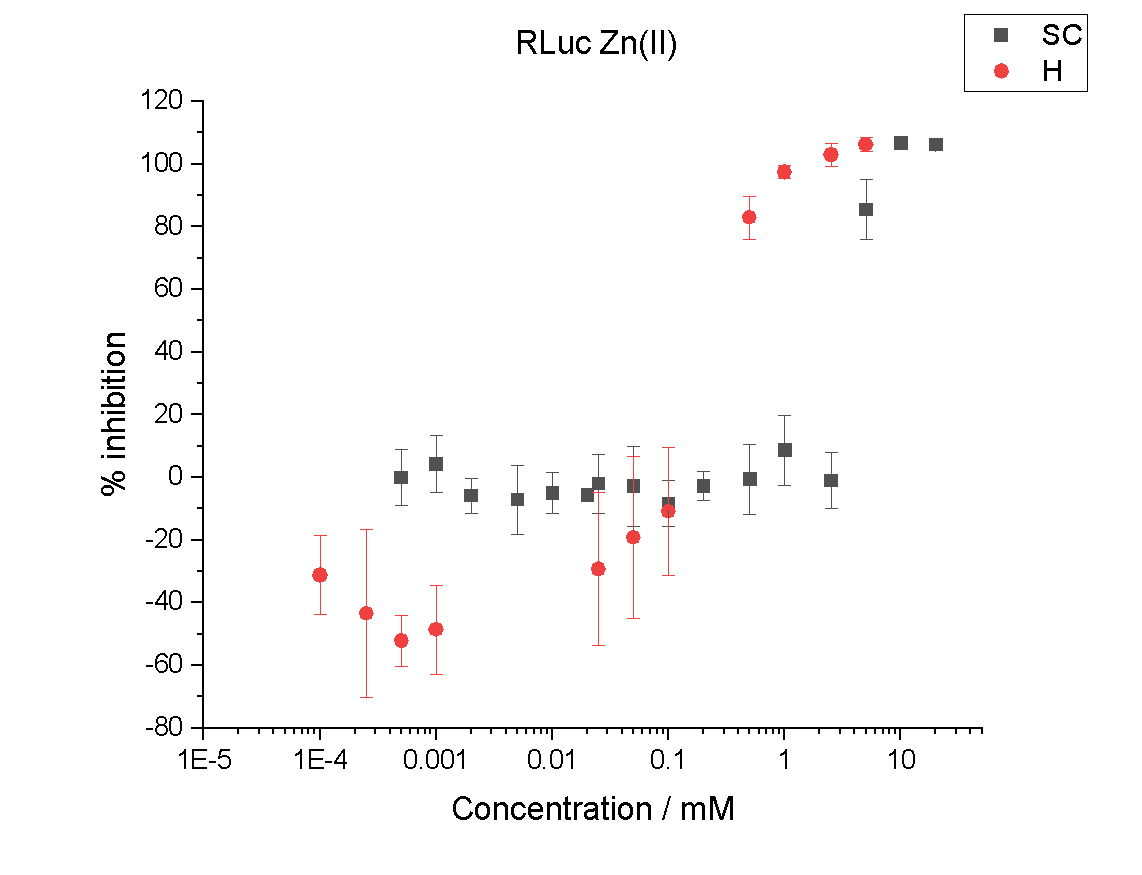 |

**Supplementary Figure S4**. IC50 curves for metal ion salt solution of Ag, Cd, Co(II), Cu(I), Cu(II), Fe(II)-1, Fe(II)-2, Fe(II)-3, Fe(III), Ga, Pb(II), Pt(II), Sn(II), Zn in screening buffer (black dots), and HEPES buffer (red dots).

## Supplementary Tables

**Supplementary Table S1**: IC50 values, as well as other parameters (slope, lower and upper limit) from the curve fitting for each luciferase activity assay in SC and in H. For samples marked in red and with “*” in their IC50 value the fitting is deemed unsuitable (R^2^ below 0.80 generally; and no fitting possible for FLuc H Cu(I)). RSE stands for residual standard error; LL and UL stand for lower and upper limit, respectively.

| Assay | Metal ion | SC | | | | | | H | | | | | |
| --- | --- | --- | --- | --- | --- | --- | --- | --- | --- | --- | --- | --- | --- |
|  |  | IC_50_ / µM | Slope | LL  (% inh) | UL  (% inh) | RSE | R^2^ | IC_50_ / µM | Slope | LL  (% inh) | UL  (% inh) | RSE | R^2^ |
| FLuc | Ag | 0.24 | 6.15 | -1.16 | 100.61 | 4.47 | 0.99 | <0.01* | 1.73 | 13.88 | 100.30 | 0.80 | 0.42 |
|  | Cd | 568.55 | 8.5 | -6.39 | 101.09 | 8.27 | 0.97 | 1.21 | 1.49 | -16.89 | 99.52 | 7.62 | 0.98 |
|  | Co(II) | 726.99 | 3.85 | 9.21 | 101.29 | 19.44 | 0.81 | 31.54 | 2.63 | -7.05 | 100.16 | 11.96 | 0.95 |
|  | Cu(I) | 408.12 | 1.94 | 9.34 | 103.08 | 12.85 | 0.92 | 0.016<IC_50_<0.063* | - | 31.61 | 100.49 | - | - |
|  | Cu(II) | 125.35 | 7.59 | 19.12 | 100.66 | 16.98 | 0.86 | 0.07 | 1.23 | -10.48 | 99.49 | 0.60 | 0.98 |
|  | Fe(II)-1 | 180.67 | 1.66 | 8.18 | 102.82 | 17.23 | 0.86 | 23.76 | 8.75 | -28.91 | 100.18 | 8.63 | 0.98 |
|  | Fe(II)-2 | 264.05 | 4.63 | 2.86 | 100.95 | 13.40 | 0.93 | 22.89 | 7.78 | -39.06 | 100.26 | 10.32 | 0.98 |
|  | Fe(II)-3 | 256.96 | 4.52 | 4.66 | 99.99 | 12.05 | 0.94 | 2.73 | 0.73 | -33.93 | 102.84 | 21.75 | 0.86 |
|  | Fe(III) | 23.91 | 2.31 | -7.48 | 100.57 | 9.75 | 0.87 | 22.32 | 3.19 | -10.40 | 102.06 | 10.24 | 0.98 |
|  | Ga | 14.94* | 1.02 | -0.47 | 102.98 | 24.02 | 0.74 | 10.54 | 3.03 | -34.51 | 99.49 | 6.75 | 0.99 |
|  | Pb(II) | 737.41 | 8.23 | 10.17 | 99.51 | 14.76 | 0.89 | 2.74 | 1.04 | -34.85 | 100.49 | 6.83 | 0.99 |
|  | Pt(II) | 42.97 | 1.78 | 25.15 | 101.05 | 7.55 | 0.95 | 39.93 | 1.22 | 2.88 | 102.20 | 10.08 | 0.95 |
|  | Sn(II) | 85.45 | 2.75 | -2.78 | 101.72 | 6.84 | 0.98 | >5000 | 0.48 | -37.68 | 146.21 | 10.66 | 0.95 |
|  | Zn | 1200.65 | 3.5 | 19.38 | 103.35 | 14.40 | 0.84 | 0.88 | 1.64 | 1.08 | 96.09 | 12.25 | 0.92 |
| NLuc | Ag | 4.50 | 1.31 | -4.56 | 123.54 | 19.53 | 0.86 | 46.63* | 1.92 | 1.09 | 104.33 | 26.20 | 0.75 |
|  | Cd | 1633.95 | 1.11 | 4.42 | 145.33 | 6.92 | 0.97 | 88.31 | 1.85 | -1.67 | 23.08 | 3.84 | 0.90 |
|  | Co(II) | 3996.84 | 0.8 | 2.60 | 183.60 | 5.43 | 0.98 | >5000 | 0.44 | -1.16 | 102.33 | 3.22 | 0.93 |
|  | Cu(I) | 46.64 | 4.14 | 1.82 | 160.88 | 6.38 | 0.99 | 56.45 | 4.05 | 2.39 | 101.82 | 4.83 | 0.99 |
|  | Cu(II) | 31.08 | 2.74 | -2.44 | 102.26 | 2.46 | 1.00 | 22.38 | 3.29 | 0.36 | 101.39 | 3.84 | 0.99 |
|  | Fe(II)-1 | 294.34 | 1.01 | 2.63 | 121.76 | 17.32 | 0.88 | 336.44 | 1.39 | 0.29 | 105.70 | 3.06 | 1.00 |
|  | Fe(II)-2 | 883.48 | 1.03 | -1.67 | 191.98 | 8.70 | 0.98 | 364.32 | 1.3 | -1.03 | 106.66 | 3.62 | 0.99 |
|  | Fe(II)-3 | 438.79 | 1.15 | 0.76 | 174.65 | 8.21 | 0.99 | 316.77 | 1.37 | 0.35 | 105.50 | 2.83 | 1.00 |
|  | Fe(III) | 816.32 | 1.24 | 5.60 | 158.19 | 8.80 | 0.97 | 130.01 | 1.97 | -1.09 | 103.38 | 3.88 | 0.99 |
|  | Ga | 1815.21 | 3.78 | 4.88 | 113.83 | 14.74 | 0.91 | 2125.75 | 2.59 | -0.50 | 102.14 | 3.20 | 0.99 |
|  | Pb(II) | >5000 | 1.23 | -2.79 | 10696.41 | 4.95 | 0.94 | 541.60 | 2.55 | 0.69 | 94.61 | 2.76 | 1.00 |
|  | Pt(II) | 258.81 | 1.11 | 0.94 | 139.67 | 4.95 | 0.99 | 246.10 | 1.77 | 1.59 | 96.23 | 3.21 | 1.00 |
|  | Sn(II) | 2099.27* | 2.6 | 2.43 | 151.38 | 4.63 | 0.99 | >5000* | 1.31 | -1.93 | 986.24 | 5.24 | 0.30 |
|  | Zn | 106.40 | 0.86 | -4.76 | 113.82 | 16.75 | 0.88 | 128.18 | 0.76 | 0.56 | 93.08 | 4.64 | 0.98 |
| RLuc | Ag | 68.44 | 2.72 | 10.51 | 106.17 | 5.37 | 0.99 | 26.98 | 3.32 | -4.31 | 106.54 | 18.46 | 0.89 |
|  | Cd | 536.75 | 2.36 | -6.88 | 87.79 | 9.15 | 0.95 | 294.56 | 0.94 | -34.43 | 119.81 | 17.58 | 0.92 |
|  | Co(II) | 688.50 | 2.86 | -0.83 | 99.26 | 8.46 | 0.96 | >5000 | 0.34 | -43.35 | 441.40 | 15.77 | 0.90 |
|  | Cu(I) | 363.64 | 17.14 | 9.93 | 106.59 | 5.96 | 0.99 | 18.51 | 3.15 | -3.74 | 106.64 | 12.61 | 0.95 |
|  | Cu(II) | 132.36 | 13.66 | -10.96 | 106.11 | 7.10 | 0.99 | 7.79 | 3.69 | -46.46 | 106.52 | 13.75 | 0.97 |
|  | Fe(II)-1 | 107.62 | 3.02 | 10.44 | 97.87 | 11.37 | 0.93 | 43.08 | 1.87 | -24.86 | 104.92 | 13.63 | 0.95 |
|  | Fe(II)-2 | 117.53 | 1.87 | -0.70 | 99.95 | 10.77 | 0.95 | 40.47 | 1.59 | -30.59 | 107.24 | 13.26 | 0.96 |
|  | Fe(II)-3 | 114.89 | 2.44 | 9.45 | 99.70 | 9.95 | 0.95 | 47.27 | 1.72 | -1.29 | 106.17 | 26.87 | 0.78 |
|  | Fe(III) | 535.61 | 0.93 | -7.14 | 119.17 | 8.58 | 0.96 | 62.54 | 1.57 | -21.88 | 106.10 | 12.48 | 0.96 |
|  | Ga | 4285.96 | 5.68 | -4.22 | 102.30 | 13.26 | 0.88 | 397.97 | 1 | -55.64 | 120.93 | 18.29 | 0.93 |
|  | Pb(II) | 2558.78 | 2.21 | -9.79 | 118.89 | 9.25 | 0.94 | 598.29 | 0.7 | -6.07 | 110.95 | 26.36 | 0.81 |
|  | Pt(II) | 350.43 | 1.29 | 7.75 | 111.89 | 6.75 | 0.97 | 723.68 | 1.5 | -0.69 | 112.49 | 10.70 | 0.94 |
|  | Sn(II) | >5000 | 11.15 | -2.22 | 2601.43 | 9.15 | 0.93 | >5000* | 4.73 | -34.27 | 25537.87 | 19.49 | 0.76 |
|  | Zn | 4268.64 | 9.01 | -1.87 | 106.49 | 9.30 | 0.95 | 196.40 | 1.57 | -41.89 | 107.57 | 9.15 | 0.95 |

**Supplementary Table S2**: Heatmap of normalised percentage of bioluminescent signal for NLuc and RLuc assays with the original EDTA concentration in screening buffer conditions (0 mM for NLuc, and 0.25 mM for RLuc) and the same EDTA concentration as in FLuc assay screening conditions (0.5 mM).

| NLuc | | | RLuc | | |
| --- | --- | --- | --- | --- | --- |
| Sample | EDTA 0 mM | EDTA 0.5 mM | Sample | EDTA 0.25 mM | EDTA 0.5 mM |
| ctrl+ | 5.07 | 5.10 | ctrl+ | 7.76 | 7.93 |
| ctrl - | 100.00 | 99.37 | ctrl - | 100.00 | 112.16 |
| Ag 0.001 mM | 94.37 | 90.76 | Ag 0.002 mM | 105.45 | 119.19 |
| Ag 0.0114 mM | 10.62 | 12.63 | Ag 0.0684 mM | 55.76 | 84.09 |
| Ag 0.05 mM | 2.31 | 4.81 | Ag 0.2 mM | 5.85 | 52.46 |
| - | - | - | Ag 1 mM | 3.07 | 0.73 |
| Cu(II) 0.0005 mM | 101.77 | 92.69 | Cu(II) 0.1 mM | 92.06 | 79.23 |
| Cu(II) 0.017 mM | 98.39 | 96.49 | Cu(II) 0.132 mM | 78.79 | 111.32 |
| Cu(II) 0.02 mM | 1.46 | 98.66 | Cu(II) 0.2 mM | 0.36 | 4.55 |
| Cu(II) 1 mM | 0.50 | 1.82 | Cu(II) 1 mM | 0.21 | 0.52 |
| Fe(II)-1 0.025 mM | 90.82 | 99.29 | Fe(II)-1 0.05 mM | 55.01 | 70.57 |
| Fe(II)-1 0.118 mM | 66.64 | 84.96 | Fe(II)-1 0.108 mM | 41.21 | 50.02 |
| Fe(II)-1 1 mM | 2.97 | 3.66 | Fe(II)-1 0.5 mM | 15.17 | 16.68 |
| Fe(II)-1 2.5 mM | 4.98 | 6.52 | Fe(II)-1 1 mM | 8.96 | 12.60 |
| Fe(II)-2 0.05 mM | 93.98 | 91.09 | Fe(II)-2 0.02 mM | 91.13 | 104.20 |
| Fe(II)-2 0.88 mM | 35.22 | 35.79 | Fe(II)-2 0.118 mM | 39.57 | 47.40 |
| Fe(II)-2 1 mM | 30.68 | 33.98 | Fe(II)-2 0.5 mM | 102.43 | 17.51 |
| Fe(II)-2 5 mM | 1.40 | 0.91 | Fe(II)-2 1 mM | 12.39 | 14.42 |
| Fe(III) 0.01 mM | 94.84 | 97.48 | Fe(III) 0.005 mM | 105.37 | 120.85 |
| Fe(III) 0.816 mM | 60.20 | 69.08 | Fe(III) 0.536 mM | 46.62 | 67.40 |
| Fe(III) 1 mM | 50.95 | 68.73 | Fe(III) 1 mM | 28.01 | 33.13 |
| Fe(III) 2.5 mM | 5.78 | 14.11 | Fe(III) 5 mM | 0.55 | 0.78 |
| Zn 0.001 mM | 101.16 | 104.19 | Zn 1 mM | 87.46 | 94.09 |
| Zn 0.174 mM | 48.56 | 102.04 | Zn 2.5 mM | 83.07 | 96.04 |
| Zn 1 mM | 2.04 | 2.94 | Zn 4.13 mM | 56.75 | 68.29 |
| Zn 5 mM | 7.26 | 9.35 | Zn 10 mM | 0.65 | 0.42 |

**Supplementary Table S3**: Heatmap of normalised percentage of bioluminescent signal for FLuc, NLuc and RLuc assays with 0 mM, 0.2 mM, and 2 mM GSH concentration in screening buffer conditions.

| FLuc | | | | NLuc | | | | RLuc | | | |
| --- | --- | --- | --- | --- | --- | --- | --- | --- | --- | --- | --- |
| Sample | GSH 0 mM | GSH 0.2 mM | GSH 2 mM | Sample | GSH 0 mM | GSH 0.2 mM | GSH 2 mM | Sample | GSH 0 mM | GSH 0.2 mM | GSH 2 mM |
| ctrl+ | 0.86 | 0.54 | 0.31 | ctrl+ | 5.79 | 5.50 | 5.41 | ctrl+ | 7.45 | 7.25 | 9.82 |
| ctrl - | 100.00 | 65.75 | 0.40 | ctrl - | 100.00 | 104.20 | 83.50 | ctrl - | 100.00 | 108.21 | 149.66 |
| Ag 0.0001 mM | 58.42 | 63.30 | 0.24 | Ag 0.001 mM | 98.89 | 102.07 | 90.91 | Ag 0.002 mM | 116.15 | 109.06 | 165.08 |
| Ag 0.000243 mM | 1.26 | 64.70 | 0.48 | Ag 0.0114 mM | 15.57 | 105.85 | 85.33 | Ag 0.0684 mM | 49.16 | 114.55 | 160.05 |
| Ag 0.0005 mM | 0.58 | 61.90 | 0.58 | Ag 0.05 mM | 2.78 | 106.96 | 85.70 | Ag 0.2 mM | 5.26 | 119.12 | 174.23 |
| Ag 1 mM | 0.43 | 0.92 | 0.53 | Ag 1 mM | 3.17 | 15.17 | 82.48 | Ag 1 mM | 2.52 | 101.75 | 171.48 |
| Cu(II) 0.05 mM | 84.51 | 54.94 | 0.48 | Cu(II) 0.0005 mM | 104.23 | 106.22 | 89.27 | Cu(II) 0.1 mM | 79.11 | 88.26 | 134.67 |
| Cu(II) 0.125 mM | 86.30 | 19.42 | 0.53 | Cu(II) 0.017 mM | 101.35 | 103.92 | 92.87 | Cu(II) 0.132 mM | 90.54 | 101.29 | 115.01 |
| Cu(II) 0.2 mM | 65.28 | 5.12 | 0.63 | Cu(II) 0.02 mM | 86.39 | 98.02 | 84.09 | Cu(II) 0.2 mM | 106.78 | 98.78 | 120.50 |
| Cu(II) 1 mM | 0.63 | 0.34 | 0.43 | Cu(II) 1 mM | 0.66 | 1.03 | 68.86 | Cu(II) 1 mM | 2.29 | 27.67 | 121.87 |
| Fe(II)-1 0.025 mM | 90.16 | 76.20 | 0.48 | Fe(II)-1 0.025 mM | 95.88 | 98.89 | 89.38 | Fe(II)-1 0.05 mM | 61.51 | 77.28 | 103.12 |
| Fe(II)-1 0.073 mM | 96.64 | 92.29 | 0.39 | Fe(II)-1 0.118 mM | 75.61 | 76.82 | 81.87 | Fe(II)-1 0.108 mM | 36.13 | 52.36 | 91.23 |
| Fe(II)-1 0.5 mM | 1.16 | 0.63 | 0.68 | Fe(II)-1 1 mM | 28.79 | 38.57 | 28.42 | Fe(II)-1 0.5 mM | 13.03 | 18.52 | 34.75 |
| Fe(II)-1 1 mM | 0.77 | 0.24 | 0.63 | Fe(II)-1 2.5 mM | 5.87 | 10.02 | 15.23 | Fe(II)-1 1 mM | 10.29 | 20.35 | 38.64 |
| Fe(II)-2 0.26 mM | 2.17 | 0.87 | 0.39 | Fe(II)-2 0.05 mM | 94.32 | 100.48 | 82.19 | Fe(II)-2 0.02 mM | 86.20 | 91.92 | 121.87 |
| Fe(II)-2 0.2 mM | 98.57 | 85.18 | 0.48 | Fe(II)-2 0.88 mM | 47.82 | 49.35 | 34.76 | Fe(II)-2 0.118 mM | 47.56 | 56.48 | 75.68 |
| Fe(II)-2 1 mM | 0.58 | 0.56 | 0.53 | Fe(II)-2 1 mM | 38.20 | 47.64 | 30.51 | Fe(II)-2 0.5 mM | 16.46 | 22.86 | 31.10 |
| - | - | - | - | Fe(II)-2 5 mM | 1.69 | 2.48 | 7.22 | Fe(II)-2 1 mM | 12.35 | 18.75 | 32.01 |
| Fe(III) 0.0001 mM | 89.68 | 66.34 | 0.34 | Fe(III) 0.01 mM | 95.59 | 100.53 | 82.66 | Fe(III) 0.005 mM | 114.55 | 116.38 | 160.74 |
| Fe(III) 0.0047 mM | 51.22 | 36.43 | 0.48 | Fe(III) 0.816 mM | 60.25 | 69.10 | 28.92 | Fe(III) 0.536 mM | 36.58 | 53.05 | 101.06 |
| Fe(III) 0.02 mM | 1.11 | 0.87 | 0.72 | Fe(III) 1 mM | 54.30 | 55.46 | 44.44 | Fe(III) 1 mM | 33.38 | 39.33 | 48.93 |
| Fe(III) 1 mM | 1.06 | 0.63 | 0.29 | Fe(III) 2.5 mM | 4.41 | 7.61 | 1.69 | Fe(III) 5 mM | 2.29 | 0.91 | 2.06 |
| Zn(II) 0.5 mM | 99.97 | 29.67 | 0.43 | Zn(II) 0.001 mM | 98.92 | 102.99 | 89.56 | Zn(II) 1 mM | 111.81 | 115.69 | 157.77 |
| Zn(II) 1 mM | 0.39 | 0.68 | 0.68 | Zn(II) 0.174 mM | 50.94 | 72.72 | 77.51 | Zn(II) 2.5 mM | 103.81 | 109.98 | 109.52 |
| Zn(II) 1.20 mM | 1.06 | 0.92 | 0.39 | Zn(II) 1 mM | 34.23 | 42.11 | 63.87 | Zn(II) 4.13 mM | 89.40 | 96.26 | 46.87 |
| Zn(II) 2.5 mM | 0.58 | 0.87 | 0.34 | Zn(II) 5 mM | 5.95 | 7.90 | 12.37 | Zn(II) 10 mM | 2.06 | 4.57 | 2.74 |

**Supplementary Table S4**: Quality control parameters (Z’, S/B, CV, for controls) for the results obtained from each of the screening plates (from sheets **3_QC, 4_QC, 5_QC** in **File_1** of SI).

| Assay | Z' | S/B | CV (CTRL_NEG) | CV (CTRL_POS) |
| --- | --- | --- | --- | --- |
| FLuc ATP+M then S then E | 0.79 | 250.40 | 6.93 | 24.04 |
| FLuc E+M then S | 0.55 | 203.59 | 14.55 | 60.68 |
| FLuc S+M then E | 0.85 | 194.26 | 4.21 | 150.19 |
| NLuc E+M then S | 0.81 | 19.49 | 5.18 | 14.71 |
| NLuc S+M then E | 0.77 | 23.78 | 6.35 | 20.46 |
| RLuc E+M then S | 0.65 | 20.79 | 9.43 | 36.95 |
| RLuc S+M then E | 0.69 | 17.93 | 7.02 | 47.10 |
| NLuc 0 mM EDTA | 0.83 | 19.74 | 0.04 | 0.27 |
| NLuc 0.5 mM EDTA | 0.71 | 19.10 | 0.07 | 0.43 |
| RLuc 0.25 mM EDTA | 0.72 | 2.82 | 27.31 | 5.98 |
| RLuc 0.5 mM EDTA | 0.60 | 3.08 | 17.29 | 3.77 |
| FLuc 0 mM GSH | 0.57 | 116.19 | 0.13 | 0.89 |
| FLuc 0.2 mM GSH | 0.58 | 120.95 | 0.13 | 0.60 |
| FLuc 2 mM GSH | -16.34 | 1.29 | 0.77 | 0.71 |
| NLuc 0 mM GSH | 0.82 | 17.26 | 0.04 | 0.22 |
| NLuc 0.2 mM GSH | 0.81 | 18.96 | 0.05 | 0.24 |
| NLuc 2 mM GSH | 0.29 | 15.43 | 0.21 | 0.16 |
| RLuc 0 mM GSH | 0.35 | 11.39 | 0.15 | 0.51 |
| RLuc 0.2 mM GSH | 0.30 | 14.93 | 0.19 | 0.41 |
| RLuc 2 mM GSH | 0.46 | 15.24 | 0.14 | 0.42 |

**2.3. List of other files attached**

**Descriptions for excel files:**

- **VALUE** – Bioluminescence readout
- **NPI** – Normalised percent inhibition
- **NSCh** – Normalised bioluminescence signal change
- **N_VALUE** - Normalised percentage of bioluminescent signal
- **Z’** – Z’-factor
- **S/B** – Signal to background ratio (mean control negative/ control positive)
- **CV** – Coefficient of Variation

**List of supplementary files with datasets**

**Table_1** Excel file with several sheets containing raw data, with different normalizations values (NPI, NSCh, N_VALUE) and t-testing for experiment.

Sheets starting with **1_** refer to those concerning the initial screenings performed for a population of 26 salts at three concentrations (0.01, 1, and 5 mM) against three luciferases (FLuc, NLuc, RLuc) and under two conditions (SC and H).

- **1_platemaps** – Excel sheet with well information for each screened plate, plus PNG attached with heatmaps.
- **1_raw data** – Excel sheet with raw data (VALUE) with different normalizations values (NPI, NSCh, N_VALUE) for all plates in the studied conditions.
- **1_FLuc SC, 1_NLuc SC, 1_RLuc SC** – Excel sheets with raw data (VALUE) with different normalizations values (NPI, NSCh, N_VALUE) and t-testing for each luciferase in screening buffer conditions.
- **1_FLuc H, 1_NLuc H, 1_RLuc H** – Excel sheets with raw data (VALUE) with different normalizations values (NPI, NSCh, N_VALUE) and t-testing for each luciferase in HEPES buffer conditions.
- **1_QC** – Excel sheet with calculated quality control parameters (Z’, S/B, CV, mean and standard deviations for controls) for the results obtained from each of the screening plates (the raw data for which are contained in sheets **1_raw data**).
- **1_Al raw data** – Excel sheet with raw data (VALUE) with different normalizations values (NPI, NSCh, N_VALUE) and t-testing for repeated sets of Al metal ion salt.
- **1_Al QC** – Excel sheet with calculated quality control parameters (Z’, S/B, CV, mean and standard deviations for controls) for the results obtained from each of the screening plates (the raw data for which are contained in sheet 1_Al raw data).

Sheets starting with **2_** refer to those concerning the testing of dose response of selected most affecting metal cations for 3 luciferases (FLuc, NLuc, RLuc) for two conditions (SC and H). The experiments were conducted in two main rounds: in the first round (experiment: 1st), all metal cations were tested within the same concentration range (sample versus luciferase/condition), and in the second round (experiment: 2nd), additional concentrations (3^rd^ round) were further tested to capture the assay response, allowing for the plotting of dose-response curves.

- **2_platemaps** – Excel sheet with well information for each screened plate.
- **2_1^st^+2^nd^** – Excel sheet with raw data (VALUE) and normalized percent inhibition (NPI) for every luciferase, condition, experiment rounds 1^st^ and 2^nd^.
- **2_3^rd^** – Excel sheet with raw data (VALUE) and normalized percent inhibition (NPI) for every luciferase, condition, 3^rd^ experiment round.
- **2_fitting** – Excel sheet with dose-response curves and fitting parameters generated in KNIME from combined data for all rounds of experiments. All curves were generated on a logarithmic scale of concentrations versus normalized percent inhibition (NPI).
- **2_QC** – Excel sheet with calculated quality control parameters (Z’, S/B, CV, mean and standard deviations for controls) for the results obtained from each of the screening plates (the raw data for which are contained in sheets **2_1^st^+2^nd^** and **2_3^rd^** ).

Sheets starting with **3_** refer to those data from experiments for all luciferases on different variants of metal cations (five metal cations in four concentrations) preincubation: first addition being enzyme (E) or substrate (S), or ATP in the case of FLuc (A).

- **3_platemaps** – Excel sheet with well information for each screened plate.
- **3_raw data** – Excel sheet with raw data (VALUE) with different normalizations values (NPI, NSCh, N_VALUE) for all plates in the studied conditions.
- **3_FLuc, 3_NLuc, 3_RLuc** – Excel sheet with raw data (VALUE) with different normalizations values (NPI, NSCh, N_VALUE) and t-testing for each luciferase in different preincubation conditions.
- **3_CTRLS** – Excel sheet with raw data (VALUE) with different normalizations values (NPI, NSCh, N_VALUE) and t-testing of only controls negative and positive for each luciferase in different preincubation conditions.
- **3_BLANKS** – Excel sheet with raw data (VALUE) with different normalizations values (NPI, NSCh, N_VALUE) and t-testing of only blanks for each luciferase in different preincubation conditions.
- **3_QC** – Excel sheet with calculated quality control parameters (Z’, S/B, CV, mean and standard deviations for controls) for the results obtained from each of the screening plates (the raw data for which are contained in sheet **3_raw data**)

Sheets starting with **4_** refer to those data from experiments for NLuc and RLuc on different variants of metal cations (five metal cations in four concentrations) in different experiments investigating the effect of EDTA concentration.

- **4_platemaps** – Excel sheet with well information for each screened plate.
- **4_raw data** – Excel sheet with raw data (VALUE) with different normalizations values (NPI, NSCh, N_VALUE) for all plates in the studied conditions.
- **4_ NLuc+RLuc** – Excel sheet with raw data (VALUE) with different normalizations values (NPI, NSCh, N_VALUE) and t-testing for each luciferase in different EDTA concentrations.
- **4_CTRLS+BLANKS** – Excel sheet with raw data (VALUE) with different normalizations values (NPI, NSCh, N_VALUE) and t-testing of only controls negative and positive, and blanks for each luciferase in different EDTA concentrations.
- **4_QC** – Excel sheet with calculated quality control parameters (Z’, S/B, CV, mean and standard deviations for controls) for the results obtained from each of the screening plates (the raw data for which are contained in sheet **4_raw data**)

Sheets starting with **5_** refer to those data from experiments for all luciferases on different variants of metal cations (five metal cations in four concentrations) in different experiments investigating the effect of glutathione (GSH) concentration.

- **5_platemaps** – Excel sheet with well information for each screened plate.
- **5_raw data** – Excel sheet with raw data (VALUE) with different normalizations values (NPI, NSCh, N_VALUE) for all plates in the studied conditions.
- **5_ FLuc+NLuc+RLuc** – Excel sheet with raw data (VALUE) with different normalizations values (NPI, NSCh, N_VALUE) and t-testing for each luciferase in different GSH concentrations.
- **5_metals** – Excel sheet with raw data (VALUE) with different normalizations values (NPI, NSCh, N_VALUE) and t-testing between only different concentrations of a given metal ion salt, and blanks for each luciferase in different GSH concentrations.
- **5_CTRLS+BLANKS** – Excel sheet with raw data (VALUE) with different normalizations values (NPI, NSCh, N_VALUE) and t-testing of only controls negative and positive, and blanks for each luciferase in different GSH concentrations.
- **5_QC** – Excel sheet with calculated quality control parameters (Z’, S/B, CV, mean and standard deviations for controls) for the results obtained from each of the screening plates (the raw data for which are contained in sheet **5_raw data**)

Sheet **6_raw data** refers to the data from experiment for all luciferases in screening buffer conditions to determine the plate effect.

**Table_2** Excel file with several sheets containing raw data, with different normalizations values (NPI, NSCh, N_VALUE) and t-testing across preincubation, EDTA, GSH for the selected metal ions and for each luciferase. The sheets names are in the format XLuc_M, where X is F,N,or R (for FLuc, NLuc, RLuc, respectively) and M is the corresponding metal ion code mentioned in methodology.
